# *s*-Triazine-Based Ligands Possessing Identical Heteroatom-Bridged Substituents—Unexpected Triazine-O Bond Cleavage

**DOI:** 10.3390/molecules30183811

**Published:** 2025-09-19

**Authors:** Vanya B. Kurteva, Rusi I. Rusew, Zhanina S. Petkova, Magdalena Angelova, Boris L. Shivachev

**Affiliations:** 1 Institute of Organic Chemistry with Centre of Phytochemistry, Bulgarian Academy of Sciences, Acad. G. Bonchev str., bl. 9, 1113 Sofia, Bulgaria; zhanina.petkova@orgchm.bas.bg; 2 Institute of Mineralogy and Crystallography “Acad. Ivan Kostov”, Bulgarian Academy of Sciences, Acad. G. Bonchev str., bl. 107, 1113 Sofia, Bulgaria; r.rusev93@gmail.com (R.I.R.); magi.caneva.13@gmail.com (M.A.); 3 Centre of Competence “Sustainable Utilization of Bio-Resources and Waste of Medicinal and Aromatic Plants for Innovative Bioactive Products” (BIORESOURCES BG), 1000 Sofia, Bulgaria; 4 National Centre of Excellence Mechatronics and Clean Technologies, 8 bul. Kliment Ohridski, 1756 Sofia, Bulgaria

**Keywords:** s-triazine based ligands, heteroatom-bridged, carboxylic acids, heterocycles, NMR, XRD, triazine-O bond cleavage

## Abstract

Metal–organic frameworks (MOFs) are materials with extremely valuable properties. The latter depend largely on the ligand used; therefore, the design of new organic linkers is a priority task today. A series of *s*-triazines possessing variable heteroatom-bridged identical substituents, useful ligands for the synthesis of MOFs, is obtained in good to excellent yields. The problem of obtaining free carboxyl groups without forming salts with nitrogen atoms is solved. The products are characterized by NMR spectra and single crystal XRD of selected samples. Unexpected O-triazine bond cleavage under basic hydrolysis conditions is observed.

## 1. Introduction

Metal–organic frameworks (MOFs) are a relatively new class of functional materials presenting hybrid crystalline two- or three-dimensional structures built from coordinately bonded metal nodes and polydentate organic linkers. Effective MOFs possess unprecedentedly high degree of porosity, significant internal surface area, crystallinity, chemical and structural flexibility, and ability for functional design and tuning [1,2,3,4,5]. These characteristics determine their extraordinary applications [6,7,8,9] in a range of key areas, such as medicine [10,11,12,13,14,15], technology [16,17,18], water and air purification [19,20,21,22,23,24,25], gas storage [26,27,28], heterogeneous catalysis [29,30,31,32,33], energy [34,35,36,37,38], etc.

The structure and properties of MOFs are strongly dependent on the ability of the ligand and the complexing agent to form stable bonds. Variable organic compounds are used in the synthesis of these remarkable materials. Generally, these are symmetrical molecules containing coordination centers, either donor or acceptor, that complex with metal salts. Among the broad variety of ligands, polycarboxylates are the most widely exploited. Compared to symmetrically substituted benzene derivatives, triazine-based ligands are less well examined [39,40,41]. The most part represent directly connected to triazine ring donor or acceptor aromatics, while the number of MOFs built from triazines with bridged substituents are quite limited. Among the latter, the materials generated from 2,4,6-tris-(4-carboxyphenoxy)-1,3,5-triazine (H_3_TCPT) [42,43,44,45,46,47,48], 4,4′,4″-((1,3,5-triazine-2,4,6-triyl)tris(azanediyl))tribenzoic acid (H_3_TATAB) [49,50,51,52,53,54], and 3,3′,3″-((1,3,5-triazine-2,4,6-triyl)tris(azanediyl))tribenzoic acid (H_3_TATMB) [55,56,57] have exhibited exceptional surface area for gas adsorption, catalysis, and sensing.

Compounds containing donor pyridine substituents constitute another prevalent class of ligands, which have been widely used for MOF synthesis, most often as linkers in combination with carboxylates [58,59,60,61,62]. To the best of our knowledge, there are no records in the literature on molecules possessing pyridine substituents attached to a s-triazine scaffold via heteroatom bridge.

Recently, we reported on the synthesis of a novel Zn-based MOF from H_3_TCPT [63]. Herein, we describe in detail the synthetic protocol and characterization of a series of s-triazine ligands bearing a variety of heteroatom-bridged substituents, either carboxylates or heterocycles. An unexpectedly observed Ar-O bond cleavage during basic hydrolysis is also mentioned.

## 2. Results and Discussion

**Scheme 1:**
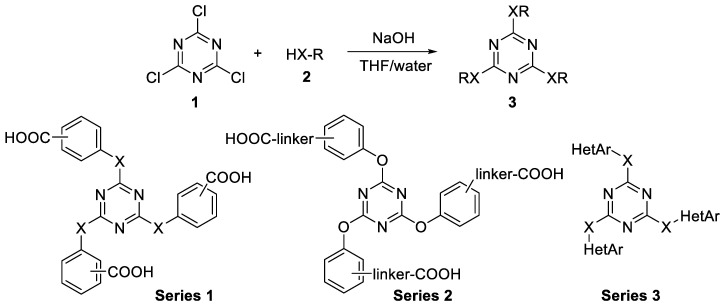
General synthetic procedure and structures of ligands **3**.

The subject of the present work is the synthesis of a series of symmetrically substituted s-triazine-based ligands designed to tune several parameters that would be important in the synthesis of MOFs, such as the type and flexibility of the bridging group, length, position and strength of the coordinating substituent, and type of the coordinating center. These ligands can be divided into three types depending on the substituents, as shown in Scheme 1. The first group includes derivatives having carboxyl groups in the substituents, which are directly linked to an aromatic ring. The position of the carboxyl group, the type of bridging heteroatom, and the length of the bridging unit are varied. In the second series, the carboxyl group is connected to an aromatic ring via a bridging chain of diverse length and type. The goal is to obtain ligands with increased mobility in the region carrying the coordinating carboxyl function, allowing for variations in orientation. The third group contains pyridine or other nitrogen containing heterocyclic substituents, differing in the location and number of the nitrogen atoms, the type of bridging heteroatom, and the length of the linker.

A modified well-known and widely applied protocol was used—a reaction between cyanuric chloride (**1**) and various aromatic compounds (HX-R, **2**) in basic conditions (Scheme 1), followed by acidification in case of carboxylic groups containing products. The conditions were optimized on the example of the known ligand H_3_TCPT (**3a**) as already published by us [63]. The main attempts were focused on solving two key points: (1) to achieve complete conversion, i.e., to avoid contamination from either starting agents or bis-substituted triazines; and (2) to obtain the compounds containing COOH groups in neutral form, not as sodium salts or hydrochlorides. The first problem was overridden by using slight excess of the reagents **2**, followed by washing with appropriate solvent, while the second one was solved by adjusted the pH to 2–3 by nitric acid instead of hydrochloric acid. The best results were obtained when the reaction was performed in aqueous solution in the presence of sodium hydroxide at room temperature and the isolated yield of 87% pure neutral ligand **3a** was achieved. This optimized protocol was applied to all examples. The results are summarized in Table 1.

All compounds from the first series were isolated in good to excellent yields except for **3g** and **3h**, which were obtained as very fine precipitates leading to serious losses during their isolation. On the contrary, the ligands of the second group **3j**–**3n** formed coarsely crystalline precipitates and were isolated in excellent yields. The third series was the most problematic. Initial attempts were focused on various pyridine derivatives. The reaction was performed by using 4-hydroxy, amino or mercapto pyridine, 3-hydroxy or amino pyridine, 2-mercaptopyridine, 4-hydroxymethyl or aminomethylpyridine, and 3-hydroxymethylpyridine, but the solid masses formed show complete insolubility in any solvent or solvent system in all cases (Table S1). Even those with methylene-bridged heteroatom and pyridine, designed in order to tune both the ligand’s flexibility and solubility, followed the same pattern. Based on these disadvantageous results the series was extended towards 2-hydroxy, amino or mercaptopyrimidinyl, and 2-hydroxy or aminopyrazinyl derivatives. The only soluble product obtained was **3o** possessing S-bridged pyrimidinyl substituents. For that reason, variable mercapto heterocycles available on the market, namely 5-methyl-1,3,4-thiadiazole-2-thiol, 4-methyl-4H-1,2,4-triazole-3-thiol, and 4-methyl-4H-1,2,4-triazole-3-thiol, were examined and ligands **3p**–**3r** were isolated in moderate to good yields.

Although the ligands were designed as potential MOF linkers, numerous experiments have been conducted to obtain frameworks from a part of the compounds, resulting in mostly amorphous materials. The only crystalline product obtained to date was synthesized from the well-known ligand **3a** [63]. The lack of MOF formation with the synthesized ligands may be attributed to several factors but is mainly related to the poor solubility in common solvents. The limited solubility hindered controlled crystallization and prevented the establishment of extended structural networks. In addition, bulky substituents or highly rigid geometries likely introduced steric and conformational constraints that reduced the accessibility of the donor groups for metal coordination. For some heteroatom-bridged systems, mismatched coordination geometry and excessive flexibility may have favored dense or amorphous phases over porous frameworks. Finally, electronic effects related to the nature of the heteroatom bridge may have reduced the binding strength of some donor sites. Taken together, these factors illustrate the challenges of translating ligand design into a successful MOF assembly. These results provoked us to increase the distance between the carboxyl group and the triazine moiety, but to preserve the geometry of the ligand by extending the aromatic system. Thus, compounds **3s** and **3t** possessing O- or NH-bridges biphenyl carboxylic acids, respectively, were also obtained. While ligand **3s** can be integrated with series 2, **3t** does not fit into any group and thus, we designated these two similar products as additional.

**Figure 1:**
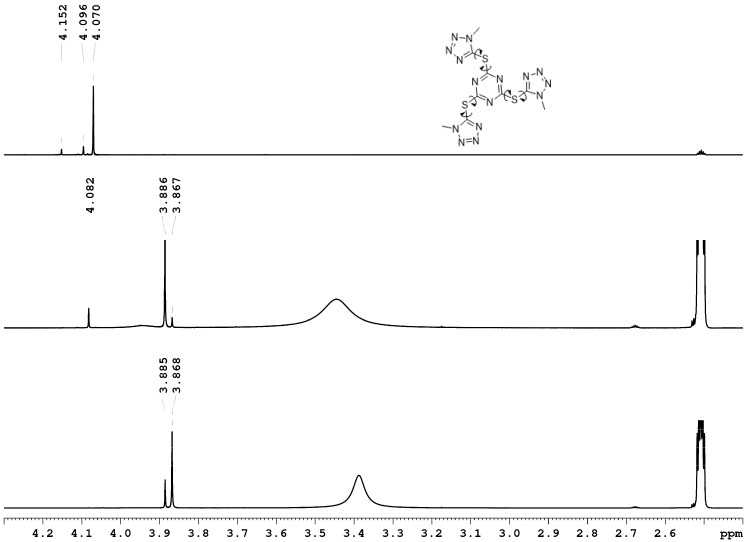
^1^H NMR spectra of **3q** in DMSO-d_6_: freshly dissolved sample at 300 K (**bottom**); the same sample at 300 K after 24 h (**middle**); the same sample at 353 K (**top**).

The structures of the ligands were assigned by 1D and 2D NMR spectra and were confirmed by a single crystal XRD of selected samples. The NMR spectra of some of the ligands, mainly N-bridged, show broad signals due to a slow exchange between two states at room temperature, which is why they are recorded at higher temperatures (Supporting Information). Compounds from group 3, possessing a *N*-methyl adjacent to the sulfur bridge, **3q** and **3r**, show an interesting pattern of behavior. The spectra of **3r** correspond to two forms in 7:3 ratio, which do not change after staying at room temperature. On the contrary, a change is observed in the spectra of ligand **3q**. In the proton spectrum of a freshly dissolved sample in DMSO at room temperature, two signals for methyl groups at 3.868 and 3.886 ppm in a ratio of 3:1 are observed, which after 1 day in the tube change their ratio to 1:12, and a third signal at 4.082 ppm appears with an intensity commensurable with that of the smaller signal. At 353 K the latter is the main one along with two new low-intensity signals at 4.096 and 4.152 ppm (Figure 1).

Apparently, the signal for the N-methyl group in the second position of the substituent is significantly affected by rotation around the six C–S bonds (three triazine-S and three tetrazole-S), falling into different shielding zones of the neighboring substituents. It can be assumed that the rotational barriers in **3q** are lower than those in **3r**, since changes are observed even at room temperature. The shifting of the methyl group signal with time or at elevated temperature to a lower field suggests that upon rotation it falls into the deshielding zone of another substituent.

**Figure 2:**
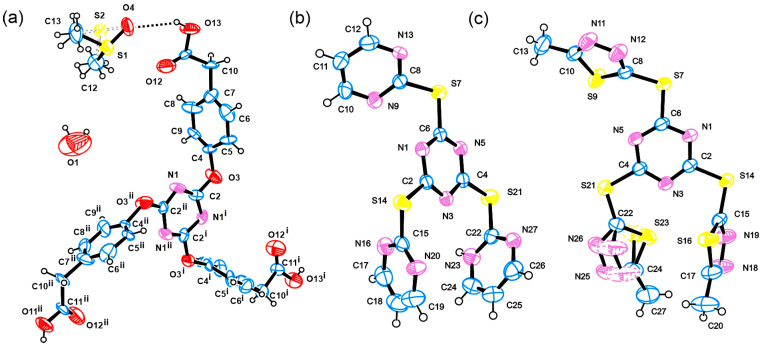
Depiction of the molecules of compounds (**a**) **3j**, (**b**) **3o,** and (**c**) **3p**; thermal ellipsoids are displayed at 50% probability, hydrogen atoms are represented as spheres with arbitrary radii while minor disorder component (in **3j** and **3p**) is shown as dashed lines; color scheme: C = blue, H = white, O = red, N = purple, and S = yellow.

Single-crystal X-ray diffraction analyses were carried out for ligands **3j**, **3o**, and **3p** (Figure 2 and Table S2). Compound **3j** crystallizes from DMSO in the trigonal space group *R*-3 with one-third molecule in the asymmetric unit. The remaining parts of the molecule are achieved through symmetry operations. The unit cell is quite large (V = 5797(5) Å^3^) and contains significant solvent-accessible voids, partially filled with solvent-disordered molecules of water and DMSO. The presence of such solvent along with the possibility to dissolve **3j** only in DMF or DMSO (hindering crystal growth of higher quality) are the main reasons for the relatively high refinement indicators (R1 = 0.240, wR2 = 0.547). On the other hand, such “weak” solubility is suitable and may be exploited in the construction of stable MOF frameworks. In the molecule of **3j** the triazine core and aromatic cresol adopts a nearly planar conformation. The peripheral substituents (C-COOH) display a certain degree of rotational freedom. The crystal packing is stabilized by weak hydrogen-bonding interactions, mainly O–H···O and C–H···S contacts. (Table S3). These non-covalent contacts interconnect the molecules into a loose supramolecular network, consistent with the limited solubility of **3j** in strongly coordinating solvents such as DMSO or DMF.

Compound **3o** crystallizes from isopropanol in the monoclinic space group *P*2_1_*/n* with four molecules in the unit cell. The parameters are identical with those of the crystals obtained from chloroform-methanol [64]. The asymmetric unit contains one ligand molecule with a nearly planar triazine fragment. The S-bridged pyrimidine substituents are oriented to maximize intermolecular interactions. The crystal packing is sustained by strong C–H···S contacts (C10–H10···S213, D···A = 3.654(2) Å) supplemented by additional weak C–H···N interactions (Table S4). These interactions organize the molecules into a three-dimensional framework.

Compound **3p** crystallizes from benzene in the triclinic space group P-1 with two molecules in the unit cell. The triazine core is essentially planar and the substituents adopt orientations that allow efficient intermolecular hydrogen bonding. The supramolecular architecture is dominated by N–H···N and C–H···S contacts, including C13–H13C···N11 (D···A = 3.570(3) Å) and C27–H27B···S92 (D···A = 3.929(4) Å), complemented by shorter C–H···S interactions (Table S5). These hydrogen bonds generate extended chains and layers, producing a compact three-dimensional network. In summary, all three structures demonstrate that the triazine core with heteroatom-bridged substituents engages in multiple non-covalent interactions (C–H···S, C–H···N, N–H···S), which play a key role in stabilizing the supramolecular packing. While **3j** is complicated by solvent disorder, **3o** and **3p** provide well-resolved models with clear supramolecular features. Importantly, the abundance of donor and acceptor sites and their demonstrated ability to generate extended hydrogen-bonded architectures underscore the suitability of these ligands as versatile linkers for the construction of stable and functional MOFs.

**Scheme 2:**
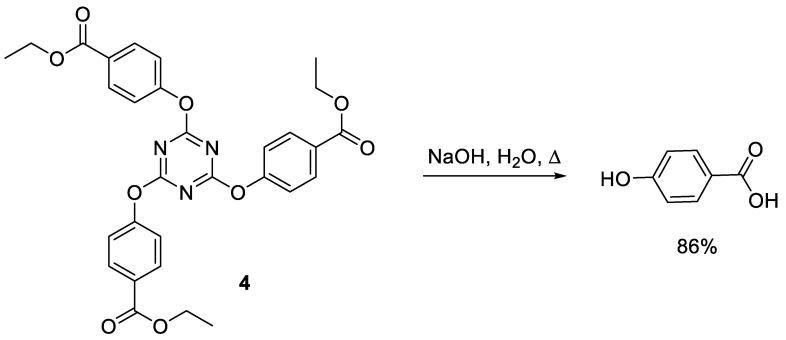
Alkaline hydrolysis of compound **4**.

Finally, it is useful to mention an unexpected result obtained during the optimization of the synthetic protocol on the example of ligand **3a**. Several parameters were varied to obtain efficiently the product in pure form, such as reagents’ proportions, dilution, the organic solvent, and the reaction duration. A two-step procedure passing through the corresponding ester was also tested with the idea of easy purification due to the solubility of the intermediate in organic solvents. The initial reaction between cyanuric chloride and ethyl 4-hydroxybenzoate led to pure ester **4** in almost quantitate yield, which was further submitted to alkaline hydrolysis (Scheme 2). Surprisingly, instead of the desired product, 4-hydroxybenzoic acid was isolated in high yield.

A literature survey shows that such a cleavage of the C–O bond is quite unexpected under these specific conditions. The cleavage of the C–O bond in diaryl ethers, which are widely distributed fragments in nature, has been extensively studied and it has been shown that the break of this strong C–O bond usually requires a metal catalyst, most often in combination with hydrogen, as summarized in a number of review articles [65,66,67,68,69]. Metal catalysis cannot be assumed in this case, as deionized water was used, indicating that triazine-based ligands should be treated with caution.

## 3. Materials and Methods

### 3.1. General

All reagents are purchased from Aldrich, Merck, and Fluka and are used without any further purification. The deuterated solvents are purchased from Deutero GmbH. The melting points are determined in capillary tubes on SRS MPA100 OptiMelt (Sunnyvale, CA, USA) automated melting point system with a heating rate of 1 °C per min. The NMR spectra are recorded on Bruker Avance NEO 400 spectrometer (Rheinstetten, Germany) in DMSO-d_6_ or CDCl_3_; the chemical shifts are quoted in ppm in δ-values against solvent peak (DMSO) or TMS as internal standard (chloroform) and the coupling constants are calculated in Hz. The assignment of the signals is confirmed by applying two-dimensional HSQC and HMBC techniques. The spectra are processed with Topspin 3.6.3 program. The mass spectra are recorded using a Q Exactive Plus Hybrid Quadrupole-Orbitrap Mass Spectrometer Thermo Scientific (HESI HRMS) in positive and/or negative mode. The spectra are processed by Thermo Scientific FreeStyle program version 1.8 SP1 (Thermo Fisher Scientific Inc., Waltham, MA, USA).

### 3.2. Synthesis of Ligands **3**

The yields are given in Table 1. The melting points of the majority of the ligands possessing carboxyl groups are above 380 °C and therefore cannot be determined by the equipment used. The proton NMR spectra of some of the ligands show broad signals due to slow exchange between two states at room temperature. At the same time, some signals in the carbon spectra are not detected and are therefore extracted from 2D experiments. For some compounds, carbon and 2D spectra are recorded by using impure samples in order to achieve higher concentrations. For simplicity, the nuclei of the aromatic substituents are indicated as Ar and the triazine unit as Tr.

Method 1. Concerning ligands possessing COOH groups (Series 1 and 2). A mixture of the acid **2a**–**2n**, **2s,** or **2t** (31 mmol), and NaOH (60 mmol) in deionized water (100 mL) was stirred until full dissolution and then a solution of cyanuric chloride (**1**, 10 mmol) in THF (30 mL) was added. The mixture was stirred at room temperature for 20 h. The solution was acidified with nitric acid to pH 2–3. The solid phase formed was filtered off and washed successively with water, methanol, and THF to give the pure ligand.

Ligand **3a**: colorless solid [70]; ^1^H NMR (DMSO-d_6_) 7.32 (AA’BB’, 6H, *J*_AB_ = 8.8, C*H*-2+6 Ar), 7.96 (AA’BB’, 6H, *J*_AB_ = 8.8, C*H*-3+5 Ar), 13.07 (bs, 3H, COO*H*); ^13^C NMR (DMSO-d_6_) 122.1 (*C*H-2+6 Ar), 129.1 (*C*_q_-4 Ar), 131.4 (*C*H-3+5 Ar), 155.0 (*C*_q_-1 Ar), 164.7 (*C*_q_ Tr), 167.3 (*C*=O); HRMS (HESI^+^) *m*/*z* calcd. for C_24_H_16_N_3_O_9_^+^ [M + H]^+^ 490.0881, found 490.0882, Δ = 0.1 mDa; HRMS (HESI^−^) *m*/*z* calcd. for C_24_H_14_N_3_O_9_^−^ [M − H]^−^ 488.0736, found 488.0736, Δ = 0 mDa.

Ligand **3b**: colorless solid; ^1^H NMR (DMSO-d_6_) 7.51–7.53 (m, 6H, C*H*-5+6 Ar), 7.73–7.74 (m, 3H, C*H*-2 Ar), 7.82–7.84 (m, 3H, C*H*-4 Ar); ^13^C NMR (DMSO-d_6_) 122.6 (*C*H-2 Ar), 126.5 (*C*H-6 Ar), 127.4 (*C*H-4 Ar), 130.4 (*C*H-5 Ar), 129.1 (*C*_q_-3 Ar), 151.7 (*C*_q_-1 Ar), 166.8 (*C*=O), 173.4 (*C*_q_ Tr); HRMS (HESI^+^) *m*/*z* calcd. for C_24_H_16_N_3_O_9_^+^ [M + H]^+^ 490.0881, found 490.0874, Δ = −0.7 mDa; HRMS (HESI^−^) *m*/*z* calcd. for C_24_H_14_N_3_O_9_^−^ [M − H]^−^ 488.0736, found 488.0736, Δ = 0 mDa.

Ligand **3c**: colorless solid [49]; ^1^H NMR (DMSO-d_6_; 373 K) 7.77–7.81 (m, 6H, C*H*-2+6 Ar), 7.89–7.93 (m, 6H, C*H*-3+5 Ar); ^13^C NMR (DMSO-d_6_; 373 K) 120.9 (*C*H-2+6 Ar), 126.3 (*C*_q_-4 Ar), 130.4 (*C*H-3+5 Ar), 142.9 (*C*_q_-1 Ar), 166.9 (*C*=O), 173.2 (*C*_q_ Tr); HRMS (HESI^+^) *m*/*z* calcd. for C_24_H_19_N_6_O_6_^+^ [M + H]^+^ 487.1361, found 487.1356, Δ = −0.5 mDa; HRMS (HESI^−^) *m*/*z* calcd. for C_24_H_17_N_6_O_6_^−^ [M − H]^−^ 485.1215, found 485.1216, Δ = 0.1 mDa.

Ligand **3d**: colorless solid [55]; ^1^H NMR (DMSO-d_6_; 373 K) 7.42 (t, 3H, *J* = 7.9, C*H*-5 Ar), 7.67 (dd, 3H, *J* = 7.7, 1.1, C*H*-6 Ar), 7.97 (dd, 3H, *J* = 8.0, 1.2, C*H*-4 Ar), 8.12 (s, 3H, C*H*-2 Ar), 10.00 (s, 3H, N*H*); ^13^C NMR (DMSO-d_6_; 373 K) 122.6 (*C*H-2 Ar), 124.9 (*C*H-6 Ar), 125.9 (*C*H-4 Ar), 129.2 (*C*H-5 Ar), 132.1 (*C*_q_-3 Ar), 139.0 (*C*_q_-1 Ar), 164.8 (*C*_q_ Tr), 167.4 (*C*=O); HRMS (HESI^+^) *m*/*z* calcd. for C_24_H_19_N_6_O_6_^+^ [M + H]^+^ 487.1361, found 487.1353, Δ = −0.8 mDa; HRMS (HESI^−^) *m*/*z* calcd. for C_24_H_17_N_6_O_6_^−^ [M − H]^−^ 485.1215, found 485.1215, Δ = 0 mDa.

Ligand **3e**: colorless solid; ^1^H NMR (DMSO-d_6_; 373 K) 3.38 (s, 9H, C*H*_3_-N), 7.45 (AA’BB’, 6H, *J*_AB_ = 8.7, C*H*-2+6 Ar), 7.93 (AA’BB’, 6H, *J*_AB_ = 8.7, C*H*-3+5 Ar); ^13^C NMR (DMSO-d_6_; 373 K) 38.6 (*C*H_3_-N), 126.9 (*C*H-2+6 Ar), 129.7 (*C*_q_-4 Ar), 130.5 (*C*H-3+5 Ar), 146.9 (*C*_q_-1 Ar), 160.6 (*C*_q_ Tr), 167.0 (*C*=O); HRMS (HESI^+^) *m*/*z* calcd. for C_27_H_25_N_6_O_6_^+^ [M + H]^+^ 529.1830, found 529.1820, Δ = −1.0 mDa; HRMS (HESI^−^) *m*/*z* calcd. for C_27_H_23_N_6_O_6_^−^ [M − H]^−^ 527.1685, found 527.1685, Δ = 0 mDa.

Ligand **3f**: colorless solid; ^1^H NMR (DMSO-d_6_; 373 K) 3.41 (s, 9H, C*H*_3_-N), 7.46 (bt, 3H, *J* = 7.8, C*H*-5 Ar), 7.55 (bd, 3H, *J* = 7.9, C*H*-6 Ar), 7.81 (bd, 3H, *J* = 7.2, C*H*-4 Ar), 7.85 (bs, 3H, C*H*-2 Ar); ^13^C NMR (DMSO-d_6_; 373 K) 38.0 (*C*H_3_-N), 127.46 (*C*H-4 Ar), 127.53 (*C*H-2 Ar), 129.3 (*C*H-5 Ar), 131.2 (*C*H-6 Ar), 132.3 (*C*_q_-3 Ar), 144.0 (*C*_q_-1 Ar), 165.3 (*C*_q_ Tr), 167.1 (*C*=O); HRMS (HESI^+^) *m*/*z* calcd. for C_27_H_25_N_6_O_6_^+^ [M + H]^+^ 529.1830, found 529.1819, Δ = −1.1 mDa; HRMS (HESI^−^) *m*/*z* calcd. for C_27_H_23_N_6_O_6_^−^ [M − H]^−^ 527.1685, found 527.1680, Δ = −0.5 mDa.

Ligand **3g**: colorless solid; ^1^H NMR (DMSO-d_6_; 373 K) 1.12 (t, 9H, *J* = 7.1, C*H*_3_- CH_2_-N), 3.89 (q, 6H, *J* = 7.1, C*H*_2_-N), 7.44 (AA’BB’, 6H, *J*_AB_ = 8.6, C*H*-2+6 Ar), 8.01 (AA’BB’, 6H, *J*_AB_ = 8.6, C*H*-3+5 Ar); ^13^C NMR (DMSO-d_6_; 373 K) 13.0 (*C*H_3_-CH_2_-N), 46.6 (*C*H_2_-N), 128.2 (*C*H-2+6 Ar), 130.3 (*C*_q_-4 Ar), 131.0 (*C*H-3+5 Ar), 144.2 (*C*_q_-1 Ar), 157.9 (*C*_q_ Tr), 167.0 (*C*=O); HRMS (HESI^+^) *m*/*z* calcd. for C_30_H_31_N_6_O_6_^+^ [M + H]^+^ 571.2300, found 571.2347, Δ = 4.7 mDa; HRMS (HESI^−^) *m*/*z* calcd. for C_30_H_29_N_6_O_6_^−^ [M − H]^−^ 569.2154, found 569.2197, Δ = 4.3 mDa.

Ligand **3h**: colorless solid; m. p. 339.1 °C (decomp. with intense gas evolution); ^1^H NMR (DMSO-d_6_; 373 K) 4.51 (bs, 6H, C*H*_2_-N), 7.36 (bd, 6H, *J* = 8.4, C*H*-2+6 Ar), 7.86 (bd, 6H, *J* = 8.4, C*H*-3+5 Ar); ^13^C NMR (DMSO-d_6_; 373 K) 44.2 (*C*H_2_-N), 127.68 (*C*H-2+6 Ar), 129.72 (*C*H-3+5 Ar), 130.2 (*C*_q_-4 Ar), 144.6 (*C*_q_-1 Ar), 165.9 (*C*_q_ Tr), 167.7 (*C*=O); HRMS (HESI^+^) *m*/*z* calcd. for C_27_H_25_N_6_O_6_^+^ [M + H]^+^ 529.1830, found 529.1819, Δ = −1.1 mDa; HRMS (HESI^−^) *m*/*z* calcd. for C_27_H_23_N_6_O_6_^−^ [M − H]^−^ 527.1685, found 527.1685, Δ = 0 mDa.

Ligand **3i**: colorless solid; m. p. 332.1 °C (decomp. with intense gas evolution); ^1^H NMR (DMSO-d_6_) 7.57 (AA’BB’, 6H, *J*_AB_ = 8.6, C*H*-2+6 Ar), 7.85 (AA’BB’, 6H, *J*_AB_ = 8.6, C*H*-3+5 Ar), 13.08 (bs, 3H, COO*H*); ^13^C NMR (DMSO-d_6_) 129.8 (*C*H-3+5 Ar), 131.4 (*C*_q_-1 Ar), 131.8 (*C*_q_-4 Ar), 134.4 (*C*H-2+6 Ar), 166.4 (*C*=O), 179.0 (*C*_q_ Tr); HRMS (HESI^+^) *m*/*z* calcd. for C_24_H_16_N_3_O_3_S_3_^+^ [M + H]^+^ 538.0196, found 538.0195, Δ = −0.1 mDa; HRMS (HESI^−^) *m*/*z* calcd. for C_24_H_14_N_3_O_3_S_3_^−^ [M − H]^−^ 536.0050, found 536.0049, Δ = −0.1 mDa.

Ligand **3j**: colorless solid; m. p. 249.6–249.8 °C (decomp.); ^1^H NMR (DMSO-d_6_) 3.60 (s, 6H, C*H*_2_-N), 7.18 (AA’BB’, 6H, *J*_AB_ = 8.7, C*H*-2+6 Ar), 7.30 (AA’BB’, 6H, *J*_AB_ = 8.7, C*H*-3+5 Ar), 12.39 (bs, 3H, COO*H*); ^13^C NMR 40.4 (*C*H_2_-N), 121.7 (*C*H-2+6 Ar), 131.0 (*C*H-3+5 Ar), 133.3 (*C*_q_-4 Ar), 150.5 (*C*_q_-1 Ar), 173.0 (*C*=O), 173.6 (*C*_q_ Tr); HRMS (HESI^+^) *m*/*z* calcd. for C_27_H_22_N_3_O_9_^+^ [M + H]^+^ 532.1351, found 532.1346, Δ = −0.5 mDa; HRMS (HESI^−^) *m*/*z* calcd. for C_27_H_20_N_3_O_9_^−^ [M − H]^−^ 530.1205, found 530.1205, Δ = 0 mDa.

Ligand **3k**: colorless solid; ^1^H NMR (DMSO-d_6_) 6.52 (d, 3H, *J* = 16.0, =C*H*-COOH), 7.29 (AA’BB’, 6H, *J*_AB_ = 8.7, C*H*-2+6 Ar), 7.59 (d, 3H, *J* = 16.0, =C*H*-C_q_-4), 7.74 (AA’BB’, 6H, *J*_AB_ = 8.7, C*H*-3+5 Ar); ^13^C NMR (DMSO-d_6_) 119.9 (=*C*H-COOH), 122.4 (*C*H-2+6 Ar), 129.9 (*C*H-3+5 Ar), 132.7 (*C*_q_-4 Ar), 143.3 (=*C*H-C_q_-4), 153.0 (*C*_q_-1 Ar), 168.0 (*C*=O), 173.4 (*C*_q_ Tr); HRMS (HESI^+^) *m*/*z* calcd. for C_30_H_22_N_3_O_9_^+^ [M + H]^+^ 568.1351, found 568.1343, Δ = −0.8 mDa; HRMS (HESI^−^) *m*/*z* calcd. for C_30_H_20_N_3_O_9_^−^ [M-H]^−^ 566.1205, found 566.1205, Δ = 0 mDa.

Ligand **3l**: colorless solid; m. p. 248.1–248.2 °C (decomp.); ^1^H NMR (DMSO-d_6_) 3.80 (s, 9H, OC*H*_3_), 6.58 (d, 3H, *J* = 16.0, =C*H*-COOH), 7.24 (d, 3H, *J* = 8.2, C*H*-6 Ar), 7.28 (dd, 3H, *J* = 8.2, 1.8, C*H*-5 Ar), 7.48 (d, 3H, *J* = 1.7, C*H*-3 Ar), 7.57 (d, 3H, *J* = 16.0, =C*H*-C_q_-4); ^13^C NMR (DMSO-d_6_) 56.5 (O*C*H_3_), 112.6 (*C*H-3 Ar), 120.2 (=*C*H-COOH), 121.8 (*C*H-5 Ar), 123.2 (*C*H-6 Ar), 134.1 (*C*_q_-4 Ar), 141.7 (*C*_q_-1 Ar), 143.6 (=*C*H-C_q_-4), 151.3 (*C*_q_-2 Ar), 168.0 (*C*=O), 173.4 (*C*_q_ Tr); HRMS (HESI^+^) *m*/*z* calcd. for C_33_H_28_N_3_O_12_^+^ [M + H]^+^ 658.1668, found 658.1671, Δ = 0.3 mDa; HRMS (HESI^−^) *m*/*z* calcd. for C_33_H_26_N_3_O_12_^−^ [M − H]^−^ 656.1522, found 656.1523, Δ = 0.1 mDa.

Ligand **3m**: colorless solid; ^1^H NMR (DMSO-d_6_) 6.56 (d, 3H, *J* = 16.1, =C*H*-COOH), 7.31 (ddd, 3H, *J* = 8.1, 2.3, 0.8, C*H*-6 Ar), 7.42 (t, 3H, *J* = 8.0, C*H*-5 Ar), 7.56 (d, 3H, *J* = 16.0, =C*H*-C_q_-4), 7.58 (dd, 3H, *J* = 8.0, 0.8, C*H*-4 Ar), 7.61 (t, 3H, *J* = 1.9, C*H*-2 Ar); ^13^C NMR (DMSO-d_6_) 120.9 (=*C*H-COOH), 121.1 (*C*H-2 Ar), 123.6 (*C*H-6 Ar), 126.5 (*C*H-4 Ar), 130.4 (*C*H-5 Ar), 136.4 (*C*_q_-3 Ar), 143.2 (=*C*H-C_q_-4), 152.2 (*C*_q_-1 Ar), 167.8 (*C*=O), 173.5 (*C*_q_ Tr); HRMS (HESI^+^) *m*/*z* calcd. for C_30_H_22_N_3_O_9_^+^ [M + H]^+^ 568.1351, found 568.1345, Δ = −0.6 mDa; HRMS (HESI^−^) *m*/*z* calcd. for C_30_H_20_N_3_O_9_^−^ [M − H]^−^ 566.1205, found 566.1205, Δ = 0 mDa.

Ligand **3n**: colorless solid; ^1^H NMR (DMSO-d_6_) 6.56 (d, 3H, *J* = 16.1, =C*H*-COOH), 7.31 (ddd, 3H, *J* = 8.4, 7.4, 0.8, C*H*-4 Ar), 7.36 (dd, 3H, *J* = 8.3, 1.2, C*H*-6 Ar), 7.46 (ddd, 3H, *J* = 8.3, 7.3, 1.6, C*H*-5 Ar), 7.61 (d, 3H, *J* = 16.1, =C*H*-C_q_-4), 7.86 (dd, 3H, *J* = 7.9, 1.5, C*H*-3 Ar); ^13^C NMR (DMSO-d_6_) 120.1 (=*C*H-COOH), 123.5 (*C*H-6 Ar), 126.6 (*C*_q_-2 Ar), 127.0 (*C*H-4 Ar), 128.2 (*C*H-3 Ar), 131.6 (*C*H-5 Ar), 136.9 (=*C*H-C_q_-4), 150.2 (*C*_q_-1 Ar), 167.7 (*C*=O), 173.6 (*C*_q_ Tr); HRMS (HESI^+^) *m*/*z* calcd. for C_30_H_22_N_3_O_9_^+^ [M + H]^+^ 568.1351, found 568.1339, Δ = −1.2 mDa; HRMS (HESI^−^) *m*/*z* calcd. for C_30_H_20_N_3_O_9_^−^ [M − H]^−^ 566.1205, found 566.1205, Δ = 0 mDa.

Ligand **3s**: colorless solid; ^1^H NMR (DMSO-d_6_; 353 K) 7.36 (bd, 6H, *J* = 8.6, C*H*-2+6 Ar), 7.72 (bd, 6H, *J* = 8.2, C*H*-2′+6′ Ar), 7.74 (bd, 6H, *J* = 8.6, C*H*-3+5 Ar), 7.98 (bd, 6H, *J* = 8.2, C*H*-3′+5′ Ar); ^13^C NMR (DMSO-d_6_; 353 K) 122.5 (*C*H-2+6 Ar), 127.1 (*C*H-2′+6′ Ar), 128.5 (*C*H-3+5 Ar), 130.3 (*C*H-3′+5′ Ar), 130.5 (*C*_q_-4′ Ar), 137.5 (*C*_q_-4 Ar), 143.8 (*C*_q_-1′ Ar), 152.1 (*C*_q_-1 Ar), 167.4 (*C*=O), 173.7 (*C*_q_ Tr); HRMS (HESI^+^) *m*/*z* calcd. for C_42_H_28_N_3_O_9_^+^ [M + H]^+^ 718.1820, found 718.1821, Δ = 0.1 mDa; HRMS (HESI^−^) *m*/*z* calcd. for C_42_H_26_N_3_O_9_^−^ [M − H]^−^ 716.1674, found 716.1681, Δ = 0.7 mDa.

Ligand **3t**: colorless solid; ^1^H NMR (DMSO-d_6_; 353 K) 7.71–7.75 (m, 8H, C*H*-3+5 + ½ C*H*-2+6), 7.77–7.80 (m, 8H, C*H*-2′+6′ + ½ C*H*-2+6), 8.02 (bd, 6H, *J* = 8.2, C*H*-3′+5′); ^13^C NMR (DMSO-d_6_; 353 K) 122.3 (*C*H-2′+6′), 126.8 (*C*H-2+6), 127.6 (*C*H-3+3), 130.1 (*C*_q_-4′), 130.4 (*C*H-3′+3′), 135.3 (*C*_q_-4), 138.3 (*C*_q_-1), 144.3 (*C*_q_-1′), 167.5 (*C*=O), 171.8 (*C*_q_ Tr); HRMS (HESI^+^) *m*/*z* calcd. for C_42_H_31_N_6_O_6_^+^ [M + H]^+^ 715.2300, found 715.2297, Δ = 0.3 mDa; HRMS (HESI^−^) *m*/*z* calcd. for C_42_H_29_N_6_O_69_^−^ [M − H]^−^ 713.2154, found 713.2171, Δ = 1.7 mDa.

Method 2. Concerning ligands possessing heterocyclic substituents (Series 3). A mixture of the heterocyclic thiol **2o**–**2r** (31 mmol) and NaOH (31 mmol) in deionized water (100 mL) was stirred until full dissolution and then a solution of cyanuric chloride (**1**, 10 mmol) in THF (30 mL) was added. The mixture was stirred at room temperature for 20 h. The solid phase formed was filtered off and washed with water (for **3o** and **3p**) or successively with water, methanol, and THF (for **3q** and **3r**) to give the pure ligand.

Ligand **3o**: colorless solid; m. p. 161.8–161.9 °C (decomp.; lit. 180–181 °C [64]); ^1^H NMR (CDCl_3_) 7.20 (t, 3H, *J* = 4.9, C*H*-4 Ar), 8.66 (d, 6H, *J* = 4.9, C*H*-3+5 Ar); ^13^C NMR (CDCl_3_) 119.9 (*C*H-4 Ar), 158.2 (*C*H-3+5 Ar), 165.0 (*C*_q_-1 Ar), 179.2 (*C*_q_ Tr); HRMS (HESI^+^) *m*/*z* calcd. for C_15_H_10_N_9_S_3_^+^ [M + H]^+^ 412.0216, found 412.0207, Δ = −0.9 mDa.

Ligand **3p**: pale yellow solid; m. p. 179.9–180.1 °C (decomp.); ^1^H NMR (CDCl_3_) 2.88 (s, 9H, C*H*_3_); ^13^C NMR (CDCl_3_) 16.0 (*C*H_3_), 154.6 (*C*_q_-1 Ar), 171.0 (*C*_q_-3 Ar), 178.1 (*C*_q_ Tr); HRMS (HESI^+^) *m*/*z* calcd. for C_12_H_10_N_9_S_6_^−^ [M + H]^+^ 471.9378, found 471.9372, Δ = −0.6 mDa.

Ligand **3q**: colorless solid; ^1^H NMR (DMSO-d_6_) m. p. 226.1 °C (decomp. with intense gas evolution); ^1^H NMR (DMSO-d_6_; predominant form at 300 K) 3.89 (s, 9H, C*H*_3_-N); ^1^H NMR (DMSO-d_6_; predominant form at 353 K) 4.0790 (s, 9H, C*H*_3_-N);^13^C NMR (DMSO-d_6_; predominant form at 353 K) 34.4 (*C*H*_3_*-N), 149.8 (*C*_q_-1 Ar), 173.8 (*C*_q_ Tr); HRMS (HESI^+^) *m*/*z* calcd. for C_9_H_10_N_15_S_3_^+^ [M + H]^+^ 424.0400, found 424.0396, Δ = −0.4 mDa.

Ligand **3r**: pale yellow solid; m. p. 269.7 °C (decomp. with intense gas evolution; 175.3 °C—sharp shrinkage with color change to orange); ^1^H NMR (DMSO-d_6_; predominant form) 3.48 (s, 9H, C*H*_3_-N), 8.76 (s, 3H, C*H*-4 Ar); ^13^C NMR (DMSO-d_6_) 32.9 (*C*H*_3_*-N), 144.4 (*C*H-4 Ar), 162.9 (*C*_q_ Tr), 168.3 (*C*_q_-1 Ar); HRMS (HESI^+^) *m*/*z* calcd. for C_12_H_13_N_12_S_3_^+^ [M + H]^+^ 421.0543, found 421.0534, Δ = −0.9 mDa; HRMS (HESI^−^) *m*/*z* calcd. for C_12_H_11_N_12_S_3_^−^ [M − H]^−^ 419.0397, found 419.0396, Δ = −0.1 mDa.

Method 3. Two-step protocol going via ester **4**. Step 1. A mixture of ethyl 4-hydroxybenzoate (31 mmol) and NaOH (30 mmol) in water (100 mL) was stirred until full dissolution and then a solution of cyanuric chloride (**1**, 10 mmol) in THF (30 mL) was added. The mixture was stirred at room temperature for 6 h. The solid phase formed was filtered off, washed with water, and dried on air. The product was purified by flash chromatography on silica gel by using DCM as a mobile phase to give the pure ester **4** in 96% yield: colorless solid; m. p. 113.3–113.4 °C; ^1^H NMR (CDCl_3_) 1.39 (t, 9H, *J* = 7.1, C*H*_3_), 4.38 (q, 6H, *J* = 7.1, C*H*_2_), 7.20 (AA’BB’, 6H, *J*_AB_ = 8.9, C*H*-2+6 Ar), 8.07 (AA’BB’, 6H, *J*_AB_ = 8.9, C*H*-3+5 Ar); ^13^C NMR (CDCl_3_) 14.3 (*C*H_3_), 61.2 (*C*H_2_), 121.4 (*C*H-2+6 Ar), 128.6 (*C*_q_-4 Ar), 131.3 (*C*H-3+5 Ar), 154.7 (*C*_q_-1 Ar), 165.6 (*C*=O), 173.3 (*C*_q_ Tr); HRMS (HESI^−^) *m*/*z* calcd. for C_30_H_26_N_3_O_9_^−^ [M − H]^−^ 572.1674, found 572.1685, Δ = 1.1 mDa.

Step 2. A solution of ester **4** (5 mmol) and NaOH (50 mmol) in water (50 mL) was refluxed with stirring for 20 h. The solution was acidified with nitric acid to pH 2–3. The solid phase formed was filtered off and washed with water to give 4-hydroxybenzoic acid in 86% yield.

### 3.3. Crystallography

A single crystal of each compound was fixed on the top of a magnetic mount using paraton N, transferred and centered on the Kappa goniometer of a Bruker D8 Venture diffractometer. Data collection was performed at ambient conditions (room temperature, 290 K) using micro-focus Mo-Kα radiation (λ = 0.71013 Å). The data reduction, e.g., unit cell parameter determination, data integration, scaling, and absorption corrections were conducted using Apex6 [71]. The structures were solved via intrinsic methods with ShelxT-2018 [72]. The structural refinement was carried out through multiple cycles of full-matrix least-squares refinement on *F*^2^ using the ShelxL-2018 package [73]. Nitrogen-bound hydrogen atoms were located from different Fourier maps, while all other hydrogen atoms were placed in idealized positions. Non-hydrogen atoms were refined anisotropically, and hydrogen atoms were refined using a riding model [73]. The most important data collection and refinement parameters for **3j**, **3o**, and **3p** are presented in Table S1.

The crystallographic data (including atomic coordinates and structure factors) in the Crystallographic Information File (CIF) were validated using the IUCr checkCIF/PLATON tool [74]. Crystallographic data from the structural analysis were deposited at the Cambridge Crystallographic Data Centre (CCDC) as follows: 2482023, 2482024 and 2482025. A copy of this data can be obtained free of charge from The Director, CCDC, 12 Union Road, Cambridge, CB2 1EZ, UK. Fax: +44 1223 336 033, e-mail: deposit@ccdc.cam.ac.uk, or www.ccdc.cam.ac.uk, accessed on 22 August 2025.

Molecular visualizations of the asymmetric units of **3j**, **3o**, and **3p** were generated using Oak Ridge Thermal-Ellipsoid Plot Program-ORTEP [75], while Mercury [76] was employed to depict the three-dimensional packing and hydrogen bonding interactions.

## 4. Conclusions

Three series of identically trisubstituted ligands based on s-triazine are obtained in good to excellent yields. The compounds possess variable heteroatom-bridged substituents, namely aromatic acids, which are directly connected to the heteroatom or distant via a chain of diverse length and type, and heterocyclic units linked through a sulfur atom. The synthetic protocol is an eco-friendly and cost-effective procedure, stirring at room temperature in water, that finishes with an easy work-up, just filtration and washing. Two important key points are solved. To avoid contamination from bis-substituted triazines, the reagents are used in slight excess and are eliminated from the final products by washing with appropriate solvents. The problem associated with releasing the sodium carboxylates without forming salts at the nitrogen atoms is solved by acidification with nitric acid. Unexpected cleavage of O-triazine bond is observed during alkaline hydrolysis of the ethyl ester of H_3_TCPT leading to almost quantitative yield of 4-hydroxybenzoic acid. Current attempts are focused on the synthesis of novel crystalline MOF materials from the ligands reported herein, both alone and in combination with a second linker.

## Figures and Tables

**Table 1:** Synthesis of ligands **3a**–**3t**.

Series 1			Series 2			Series 3		
Substituent XR		Yield, %	Substituent OAr		Yield, %	Substituent XAr		Yield, %
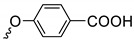	**3a**	87 ^1^	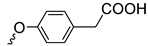	**3j**	99	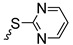	**3o**	84
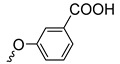	**3b**	89	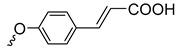	**3k**	96	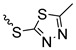	**3p**	87
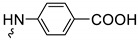	**3c**	91	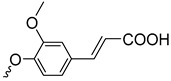	**3l**	91	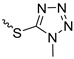	**3q**	88
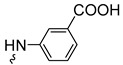	**3d**	93						
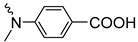	**3e**	65	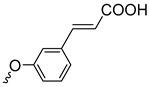	**3m**	96	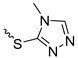	**3r**	67
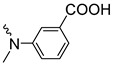	**3f**	66						
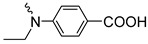	**3g**	48	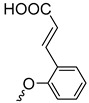	**3n**	88	**Additional**		
						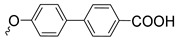	**3s**	89
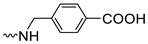	**3h**	55				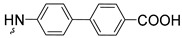	**3t**	69
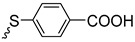	**3i**	90						

^1^ Published in ref. [63].

## Data Availability

Data are contained within the article and Supplementary Materials.

## Supplementary Materials

The following supporting information can be downloaded at https://www.mdpi.com/article/10.3390/molecules30183811/s1, Table S1: Reagents leading to insoluble solid masses; Table S2: Crystal data and structure refinement for **3j**, **3o,** and **3p**.; Table S3: Hydrogen bonds for **3j**; Table S4: Hydrogen bonds for **3o**; Table S5: Hydrogen bonds for **3p**; Figures S1–S67: NMR spectra; Figures S68–S105: HRMS spectra.

## Abbreviations

The following abbreviations are used in this manuscript:

MOF(s)	Metal–organic framework(s)
H_3_TCPT	2,4,6-tris-(4-carboxyphenoxy)-1,3,5-triazine
H_3_TATAB	4,4′,4″-((1,3,5-triazine-2,4,6-triyl)tris(azanediyl))tribenzoic acid
H_3_TATMB	3,3′,3″-((1,3,5-triazine-2,4,6-triyl)tris(azanediyl))tribenzoic acid
NMR	Nuclear magnetic resonance
XRD	X-Ray diffraction
HESI HRMS	Heated electrospray ionization high resolution mass spectrometry
DMSO-d_6_	Deuterated dimethyl sulfoxide
CDCl_3_	Deuterochloroform
TMS	Tetramethyl silane
HSQC	Heteronuclear single quantum coherence
HMBC	Heteronuclear multiple bond correlation
ROESY	Rotating-frame nuclear overhauser effect spectroscopy
THF	Tetrahydrofuran
DCM	Dichloromethane
COST	Cooperation in Science and Technology
