# Supplementary material for: s-Triazine-Based Ligands Possessing Identical Heteroatom-Bridged Substituents—Unexpected Triazine-O Bond Cleavage
Source: Molecules. 2025 Sep 19;30(18):3811. doi: 10.3390/molecules30183811 (PMC12472477; doi:10.3390/molecules30183811)
Supplement: Supplementary file 1 [file molecules-30-03811-s001.zip › molecules-3867340-supplementary.pdf]

Supporting Information

s-Triazine-based ligands possessing identical heteroatom-bridged substituents. Unexpected triazine-O bond cleavage.

Vanya B. Kurteva [1,*], Rusi I. Rusew [1], Zhanina S. Petkova [1,3], Magdalena Angelova [2] and Boris L. Shivachev [2,4,*]

[1] Institute of Organic Chemistry with Centre of Phytochemistry, Bulgarian Academy of Sciences, Acad. G. Bonchev str., bl. 9, 1113 Sofia, Bulgaria

[2] Institute of Mineralogy and Crystallography "Acad. Ivan Kostov", Bulgarian Academy of Sciences, Acad. G. Bonchev str., bl. 107, 1113 Sofia, Bulgaria

[3] Centre of Competence "Sustainable Utilization of Bio-resources and Waste of Medicinal and Aromatic Plants for Innovative Bioactive Products" (BIORESOURCES BG), Sofia 1000, Bulgaria

[4] National Centre of Excellence Mechatronics and Clean Technologies, 8 bul. Kliment Ohridski, P.C 1756, Sofia, Bulgaria

* Correspondence: vanya.kurteva@orgchm.bas.bg; blshivachev@gmail.com

Table of content

**Content**	**Page**
Table S1	2
Table S2. Crystal data and structure refinement for **3j**, **3o** and **3p**.	3
Table S3. Hydrogen Bonds for **3j**.	4
Table S4. Hydrogen Bonds for **3o**.	4
Table S5. Hydrogen Bonds for **3p**.	4
NMR spectra of compound **3a**	5
NMR spectra of compound **3b**	6
NMR spectra of compound **3c**	8
NMR spectra of compound **3d**	9
NMR spectra of compound **3e**	11
NMR spectra of compound **3f**	12
NMR spectra of compound **3g**	14
NMR spectra of compound **3h**	15
NMR spectra of compound **3i**	17
NMR spectra of compound **3j**	18
NMR spectra of compound **3k**	20
NMR spectra of compound **3l**	21
NMR spectra of compound **3m**	23
NMR spectra of compound **3n**	24
NMR spectra of compound **3o**	26
NMR spectra of compound **3p**	27
NMR spectra of compound **3q**	29
NMR spectra of compound **3r**	30
NMR spectra of compound **3s**	32

NMR spectra of compound **3t**	34
NMR spectra of ester **4**	36
NMR spectra of 4-hydroxybenzoic acid	37
HRMS spectra of ligand **3a**	38
HRMS spectra of ligand **3b**	39
HRMS spectra of ligand **3c**	39
HRMS spectra of ligand **3d**	40
HRMS spectra of ligand **3e**	40
HRMS spectra of ligand **3f**	41
HRMS spectra of ligand **3g**	41
HRMS spectra of ligand **3h**	42
HRMS spectra of ligand **3i**	42
HRMS spectra of ligand **3j**	43
HRMS spectra of ligand **3k**	43
HRMS spectra of ligand **3l**	44
HRMS spectra of ligand **3m**	44
HRMS spectra of ligand **3n**	45
HRMS spectra of ligand **3o**	45
HRMS spectra of ligand **3p**	45
HRMS spectra of ligand **3q**	46
HRMS spectra of ligand **3r**	46
HRMS spectra of ligand **3s**	46
HRMS spectra of ligand **3t**	47
HRMS spectra of ester **4**	47

Table S1. Reagents leading to insoluble solid masses.

Pyridines			Pyrimidines	Pyrazines
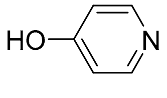	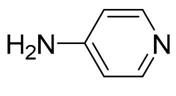	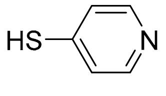	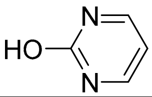	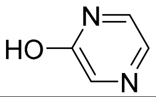
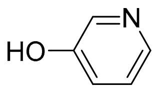	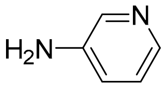	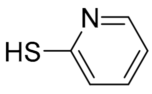	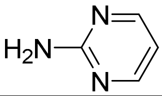	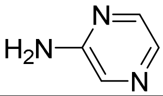
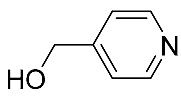	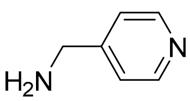	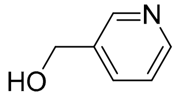		

Table S2. Crystal data and structure refinement for **3j**, **3o** and **3p**.

Identification code	**3j**	**3o**	**3p**
Empirical formula	$C_{33}H_{42}N_3O_{13.5}S_3$	$C_{15}H_9N_9S_3$	$C_{12}H_9N_9S_6$
Formula weight	792.87	411.49	471.64
Temperature/K	290	290.00	273.15
Crystal system	trigonal	monoclinic	triclinic
Space group	*R*-3	$P2_1/n$	*P*-1
a/Å	24.022(10)	9.051(3)	8.6214(3)
b/Å	24.022(10)	18.226(7)	10.4479(3)
c/Å	11.807(9)	11.228(4)	11.9290(4)
α/°	90	90	107.2420(10)
β/°	90	108.064(13)	96.8380(10)
γ/°	120	90	104.0160(10)
Volume/$Å^3$	5900(6)	1761.0(11)	974.32(6)
Z	6	4	2
$\rho_{calc}g/cm^3$	1.339	1.552	1.608
μ/mm^{-1}	0.254	0.443	0.721
$F_{(000)}$	2502.0	840.0	480.0
Crystal size/mm^3	0.5 × 0.1 × 0.1	0.1 × 0.1 × 0.05	0.2 × 0.15 × 0.12
Radiation	Mo*K*α (λ = 0.71073)	Mo*K*α (λ = 0.71073)	Mo*K*α (λ = 0.71073)
2Θ range for data collection/°	3.966 to 50.016	4.422 to 52.808	4.268 to 52.798
Reflections collected	35005	42968	54572
Independent reflections	2302	3599	3980
Data/restraints/parameters	2302/0/178	3599/0/244	3980/0/275
GOFon $F^2/R_{int}/R_{sigma}$	1.093/0.1014/0.0381	1.060/0.0387/0.0200	1.026/0.0254/0.0108
Final *R* indexes [$I>=2\sigma (I)$]	R_1 = 0.0723, wR_2 = 0.1629	R_1 = 0.0322, wR_2 = 0.0774	R_1 = 0.0335, wR_2 = 0.0922
Final *R* indexes [all data]	R_1 = 0.0923, wR_2 = 0.1742	R_1 = 0.0343, wR_2 = 0.0789	R_1 = 0.0356, wR_2 = 0.0943
Largest diff. peak/hole/e $Å^{-3}$	0.34/-0.70	0.26/-0.38	0.50/-0.38

Table S3. Hydrogen Bonds for **3j**.

D	H	A	d(D-H)/Å	d(H-A)/Å	d(D-A)/Å	D-H-A/°
O13	H13	S2	0.82	2.70	3.391(4)	143.3
O13	H13	O4	0.82	1.84	2.619(5)	158.1
C12	H12F	O12[1]	0.96	2.44	3.377(6)	163.9
C13	H13C	O4[2]	0.96	2.65	3.322(6)	127.0

[1] *4/3-y,2/3+x-y,-1/3+z;* [2] *+y,1-x+y,1-z*

Table S4. Hydrogen Bonds for **3o**.

D	H	A	d(D-H)/Å	d(H-A)/Å	d(D-A)/Å	D-H-A/°
C10	H10	S21[3]	0.93	3.01	3.654(2)	127.5

[1] *1/2+x,1/2-y,-1/2+z*

Table S5. Hydrogen Bonds for **3p**.

D	H	A	d(D-H)/Å	d(H-A)/Å	d(D-A)/Å	D-H-A/°
C13	H13C	S7[1]	0.96	2.90	3.434(3)	116.0
C13	H13C	N1[1]	0.96	2.62	3.570(3)	170.1
C27	H27B	S9[2]	0.96	2.98	3.929(4)	171.8

[1] *1+x,+y,+z;* [2] *+x,1+y,1+z*

NMR spectra of compound **3a**

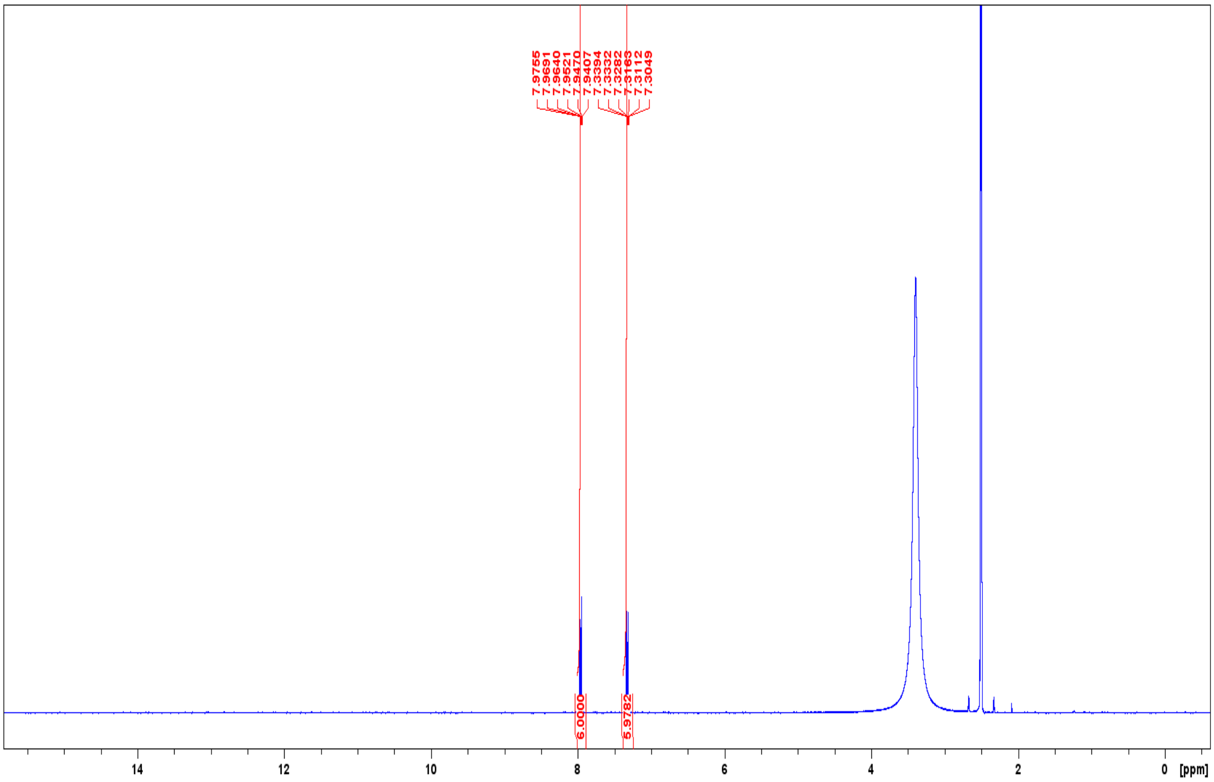

Figure S1. ^{1}H NMR spectrum of ligand **3a** in DMSO-d6.

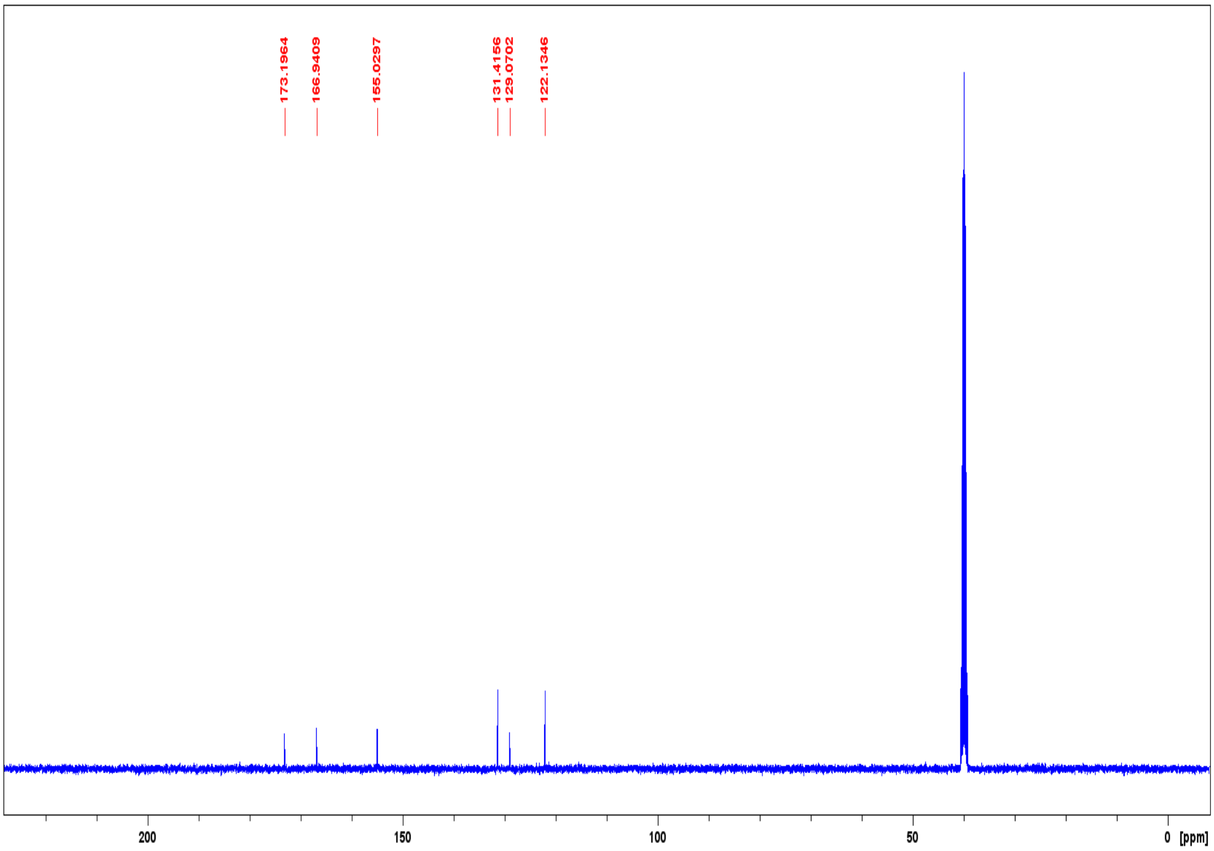

Figure S2. ^{13}C NMR spectrum of ligand **3a** in DMSO-d6.

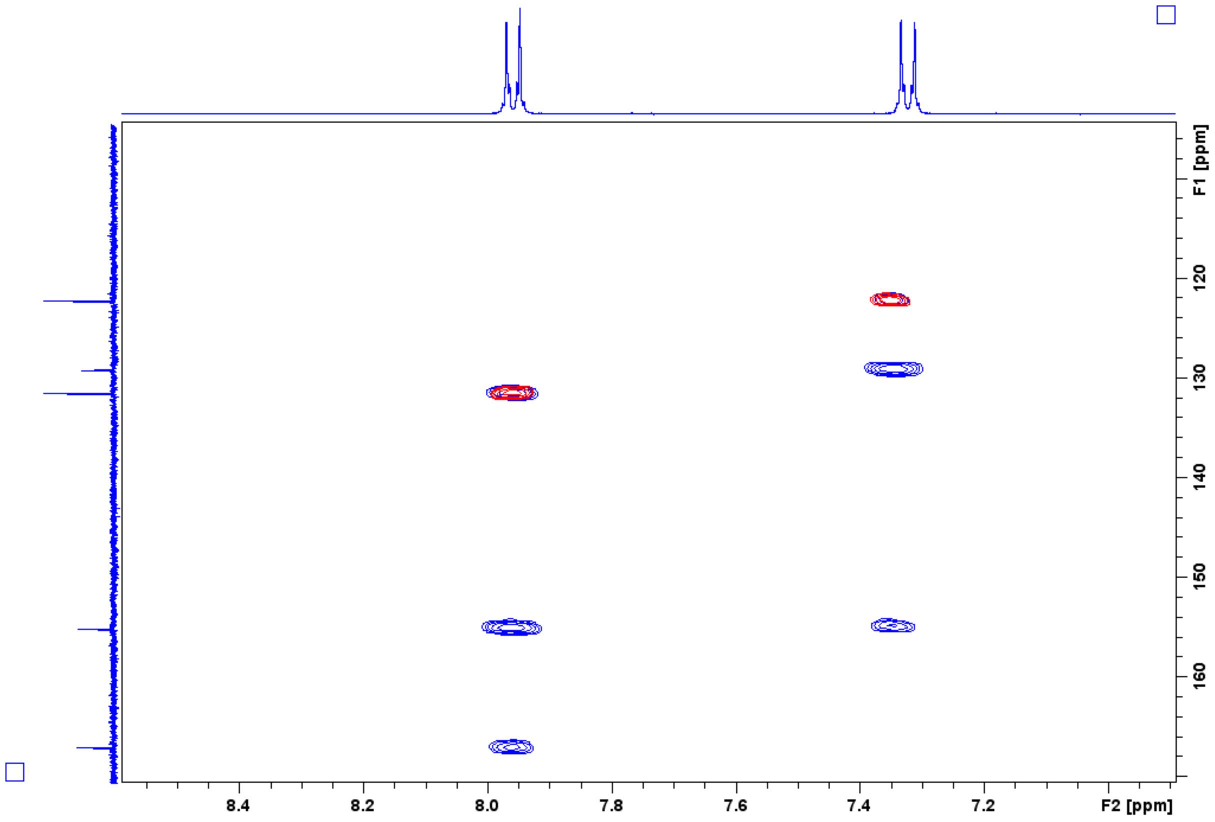

Figure S3. ^{1}H-^{13}C NMR experiments HSQC (red) and HMBC (blue) of ligand **3a** in DMSO-d_6.

<u>NMR spectra of compound **3b**</u>

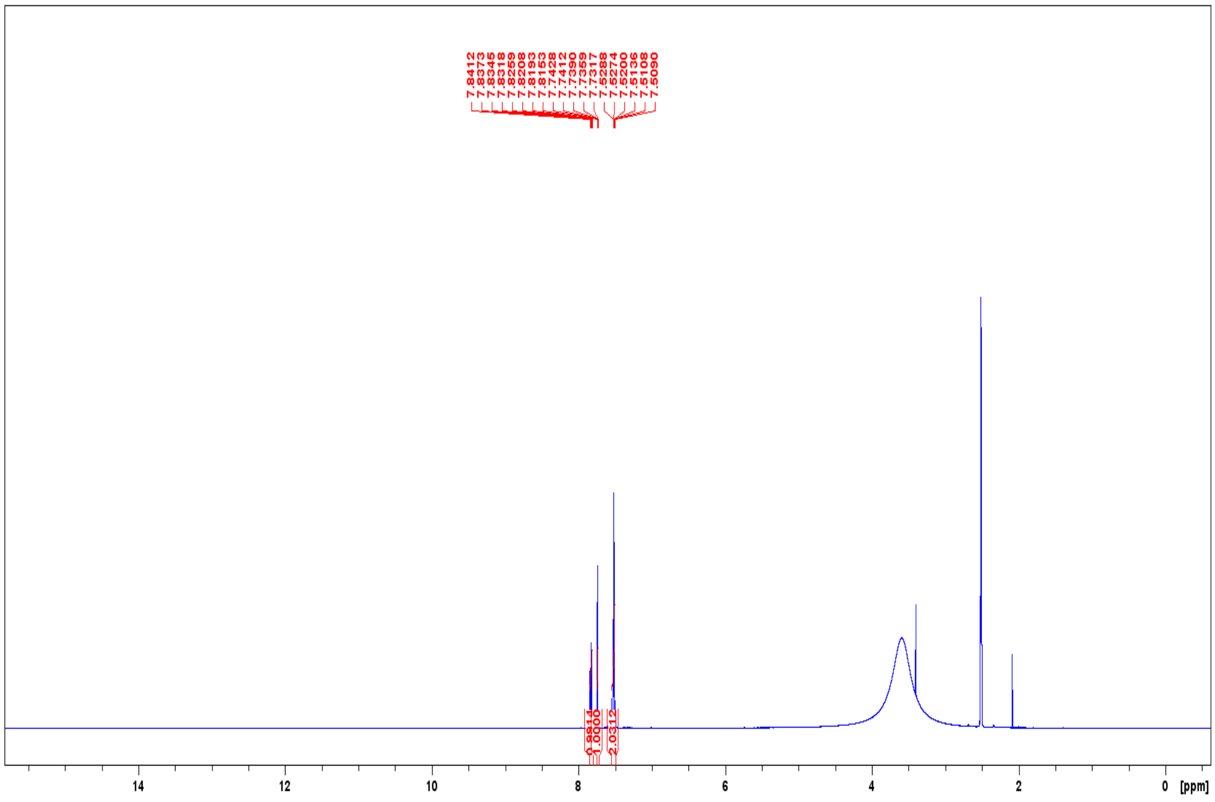

Figure S4. ^{1}H NMR spectrum of ligand **3b** in DMSO-d_6.

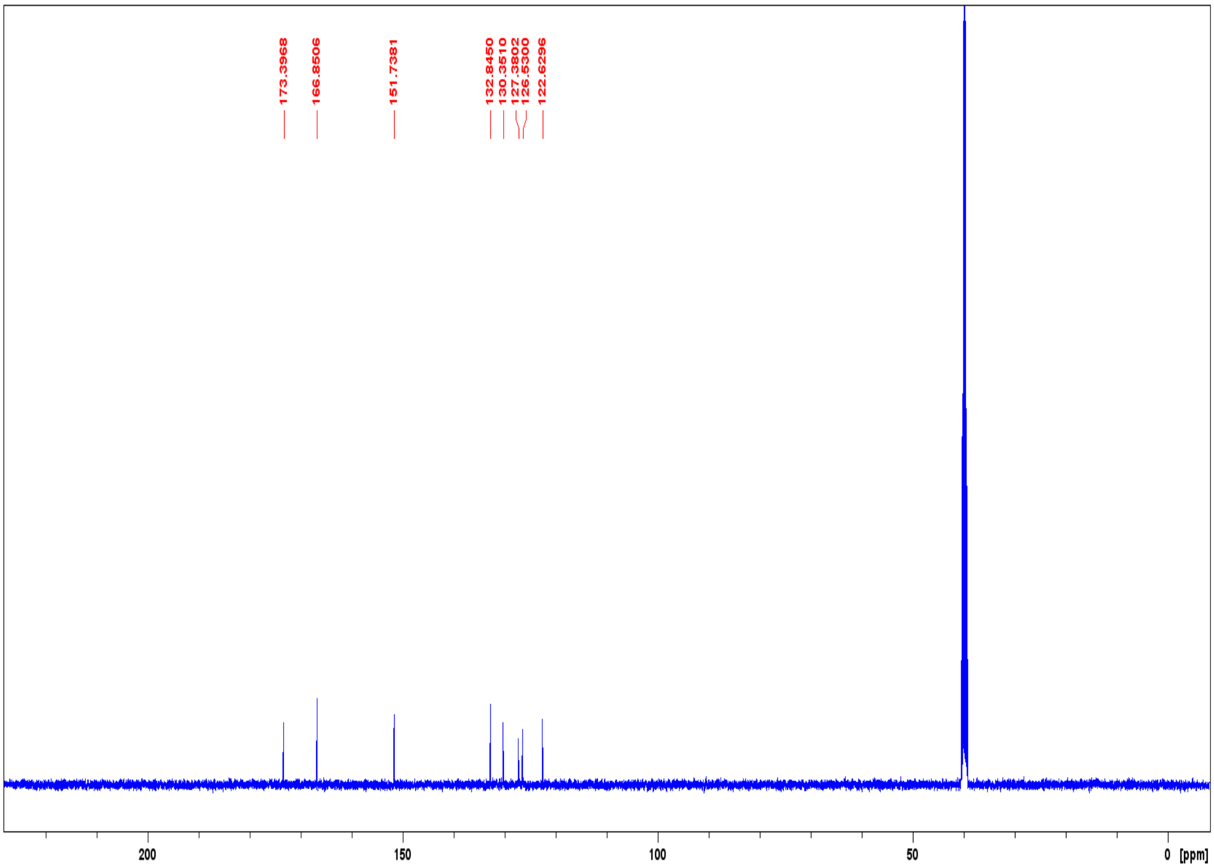

Figure S5. ^{13}C NMR spectrum of ligand **3b** in DMSO-d6.

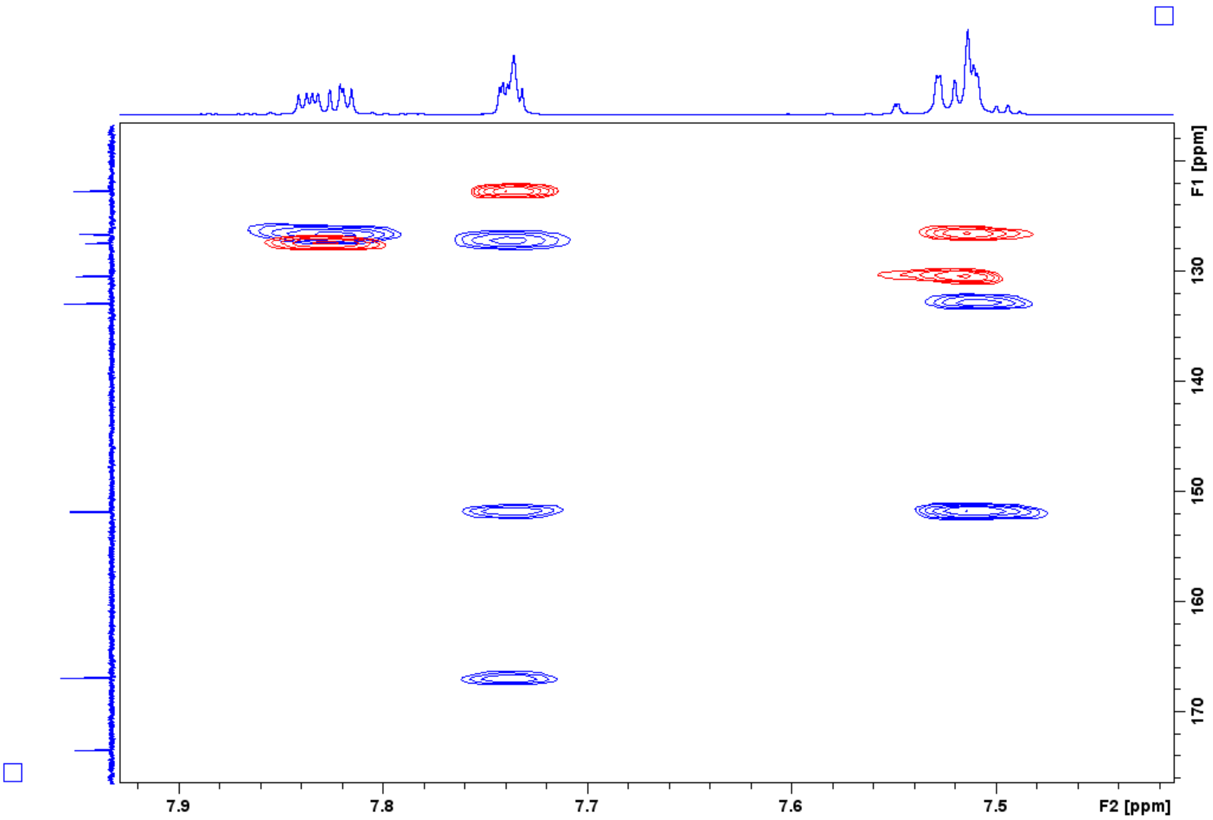

Figure S6. ^{1}H-^{13}C NMR experiments HSQC (red) and HMBC (blue) of ligand **3b** in DMSO-d6.

NMR spectra of compound **3c**

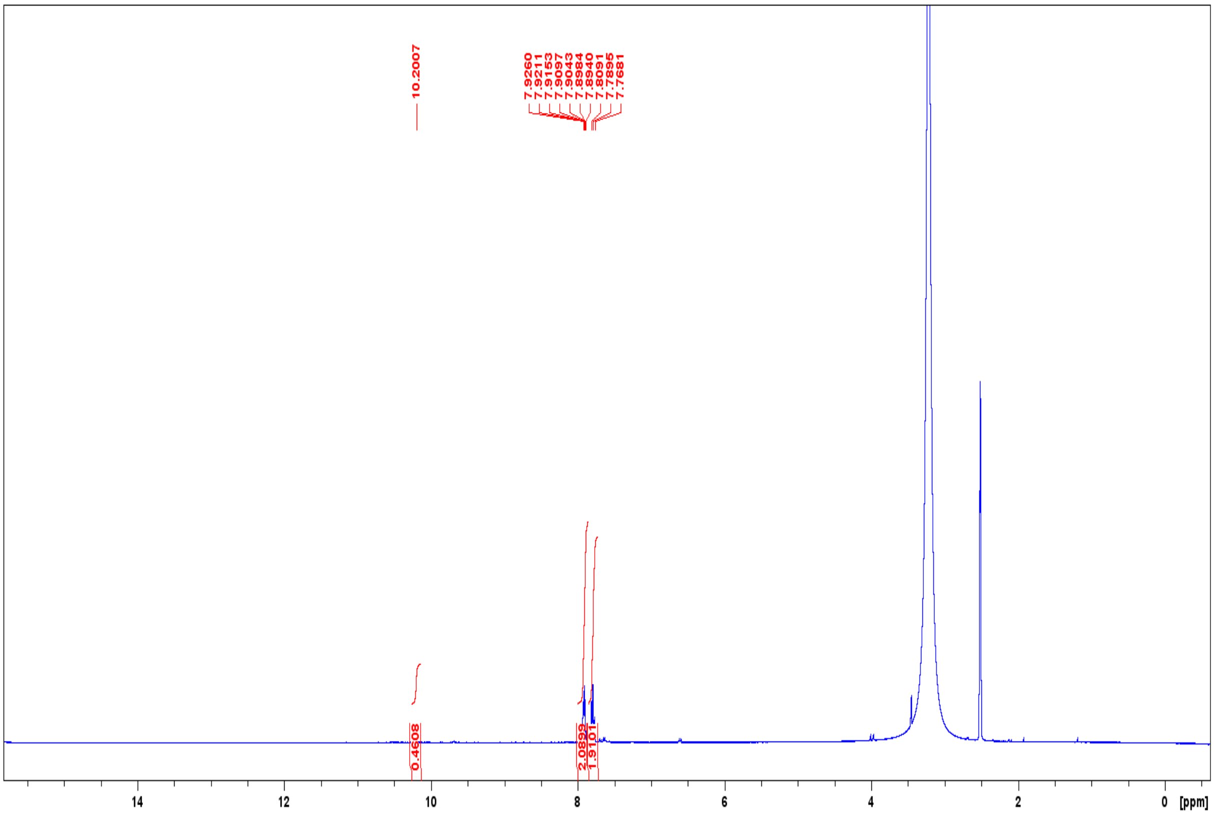

Figure S7. ^{1}H NMR spectrum of ligand **3c** in DMSO-d_6 at 373 K.

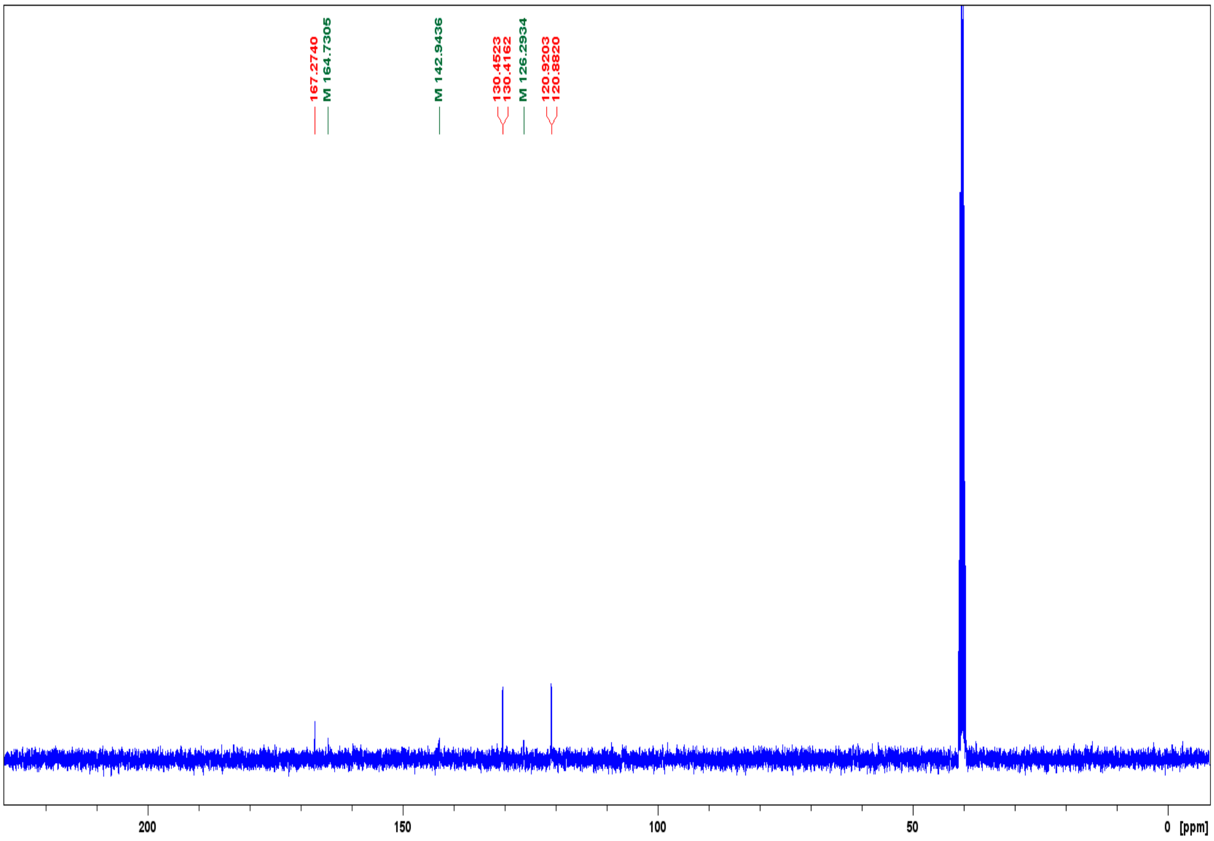

Figure S8. ^{13}C NMR spectrum of ligand **3c** in DMSO-d_6. at 373 K.

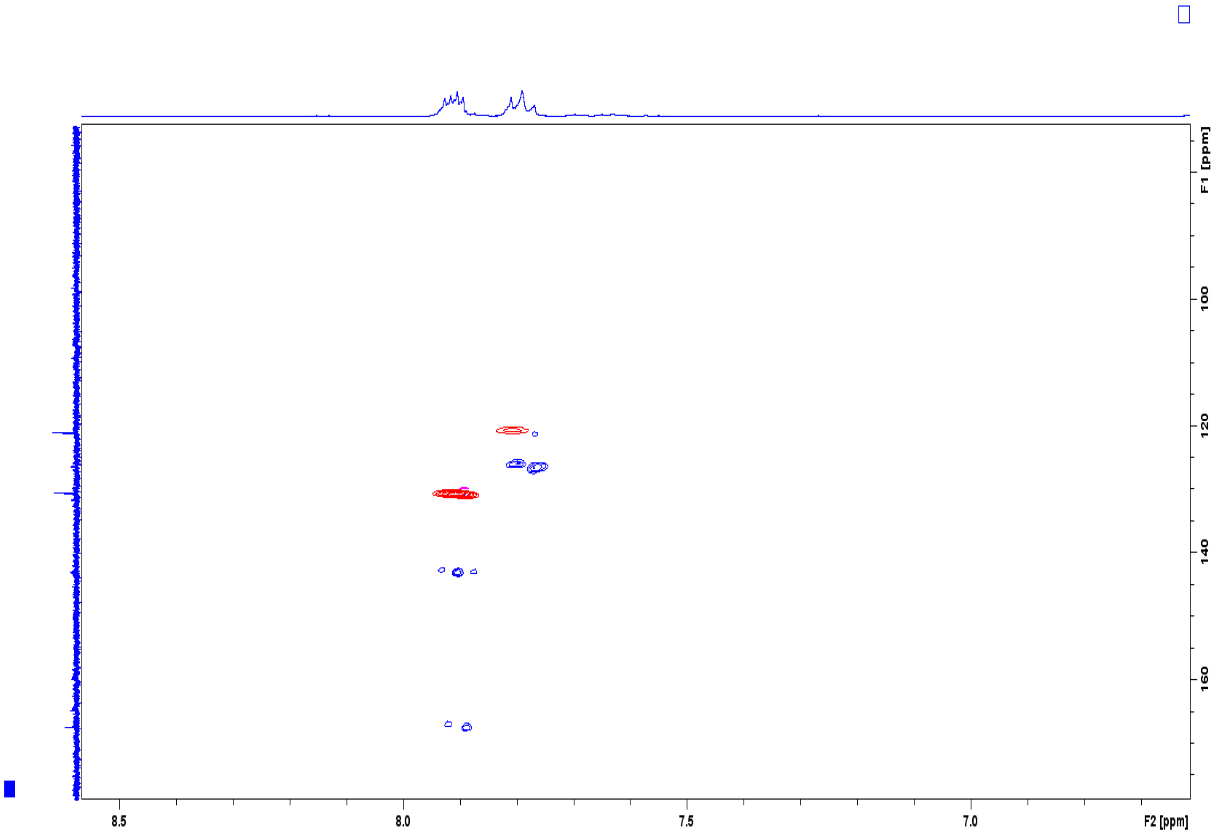

Figure S9. ^1H-^{13}C NMR experiments HSQC (red) and HMBC (blue) of ligand **3c** in DMSO-d_6. at 373 K

<u>NMR spectra of compound **3d**</u>

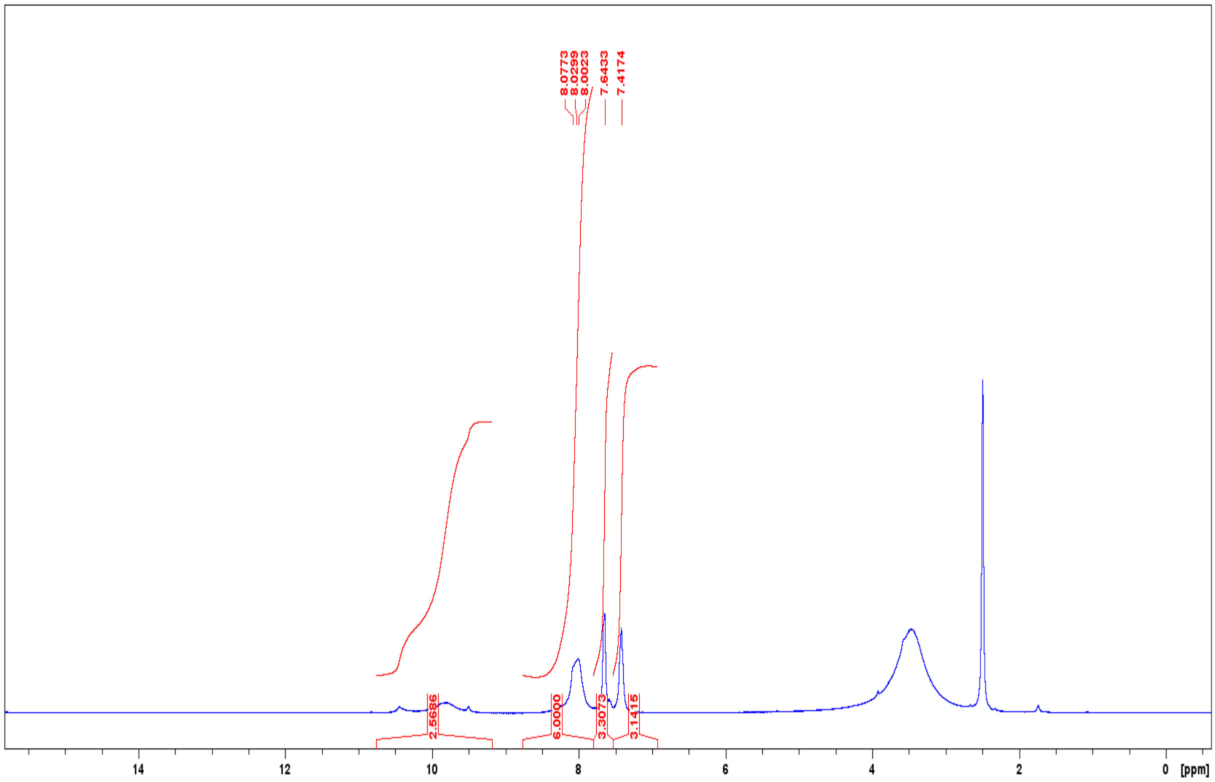

Figure S10. ^1H NMR spectrum of ligand **3d** in DMSO-d_6 at 300 K.

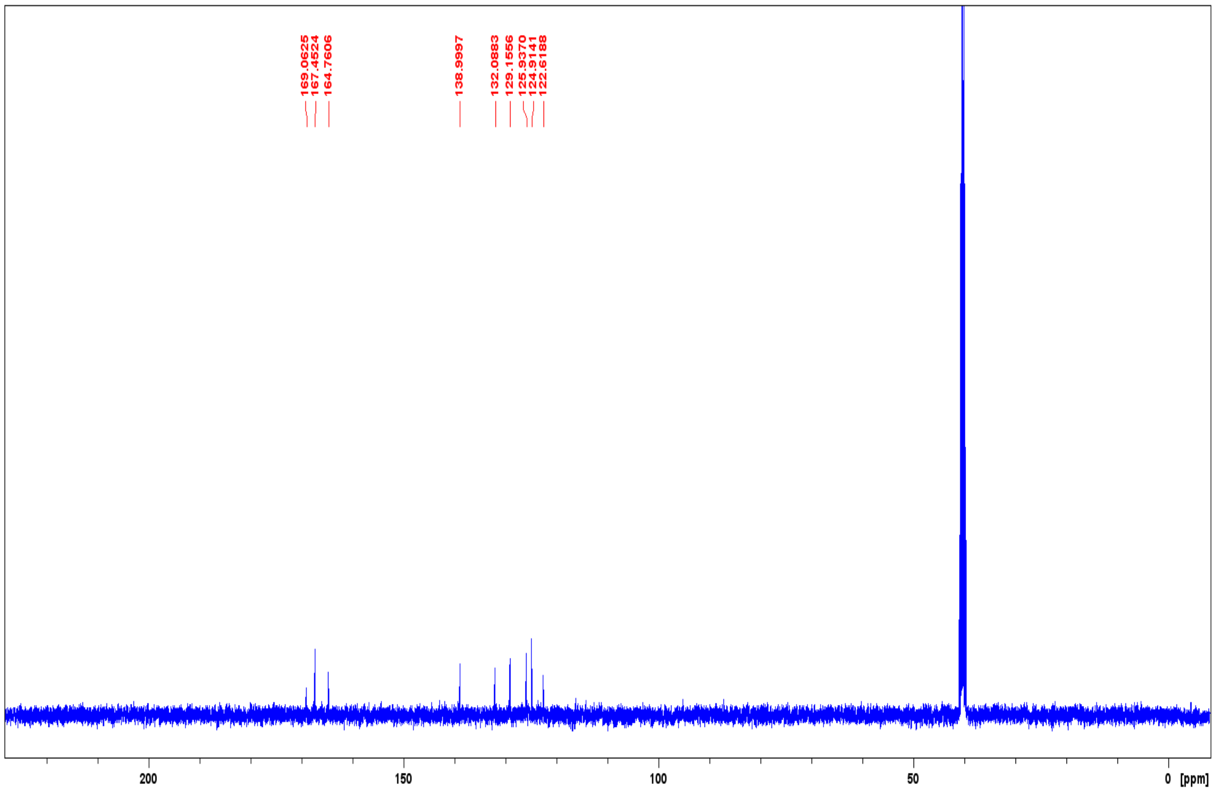

Figure S11. ^{13}C NMR spectrum of ligand **3d** in DMSO-d₆ at 373 K.

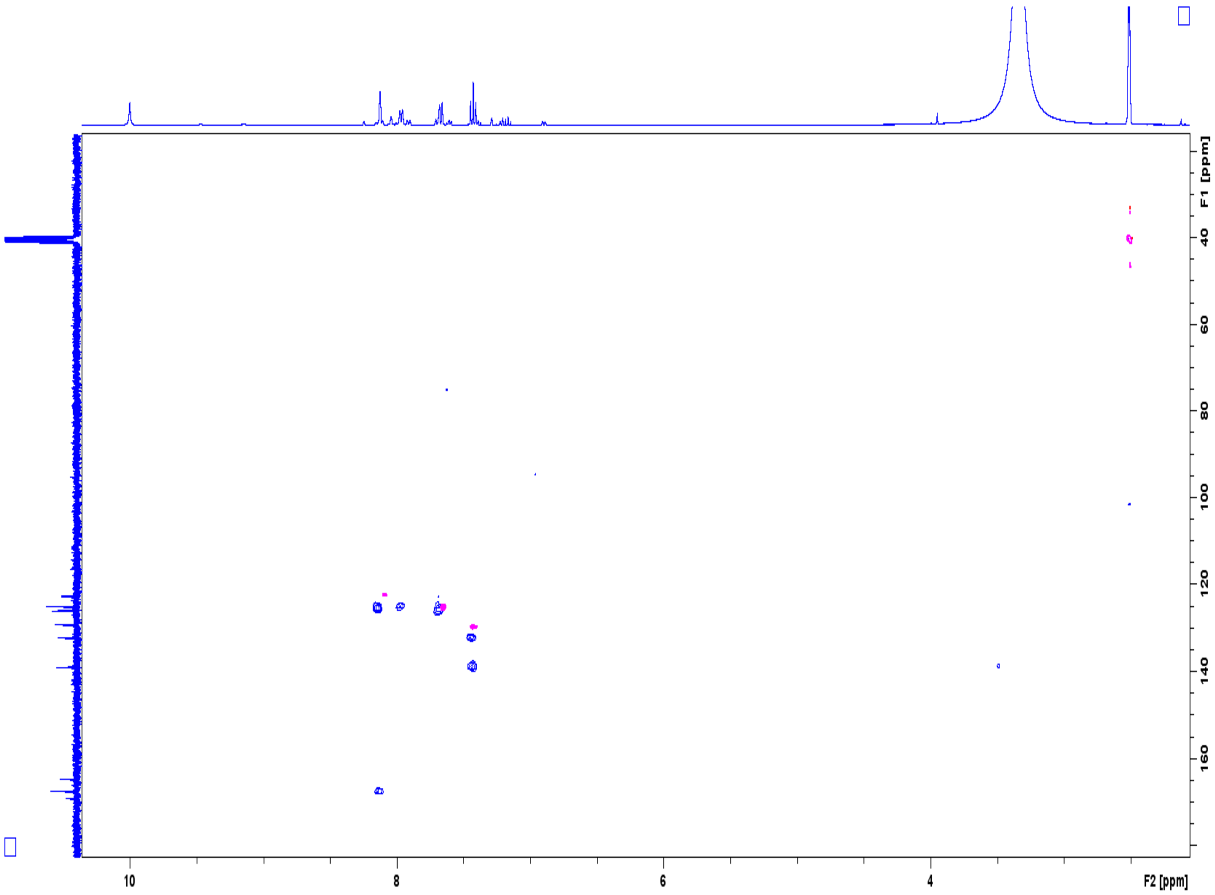

Figure S12. ^{1}H-^{13}C NMR experiments HSQC (red) and HMBC (blue) of ligand **3d** in DMSO-d₆ at 373 K.

NMR spectra of compound **3e**

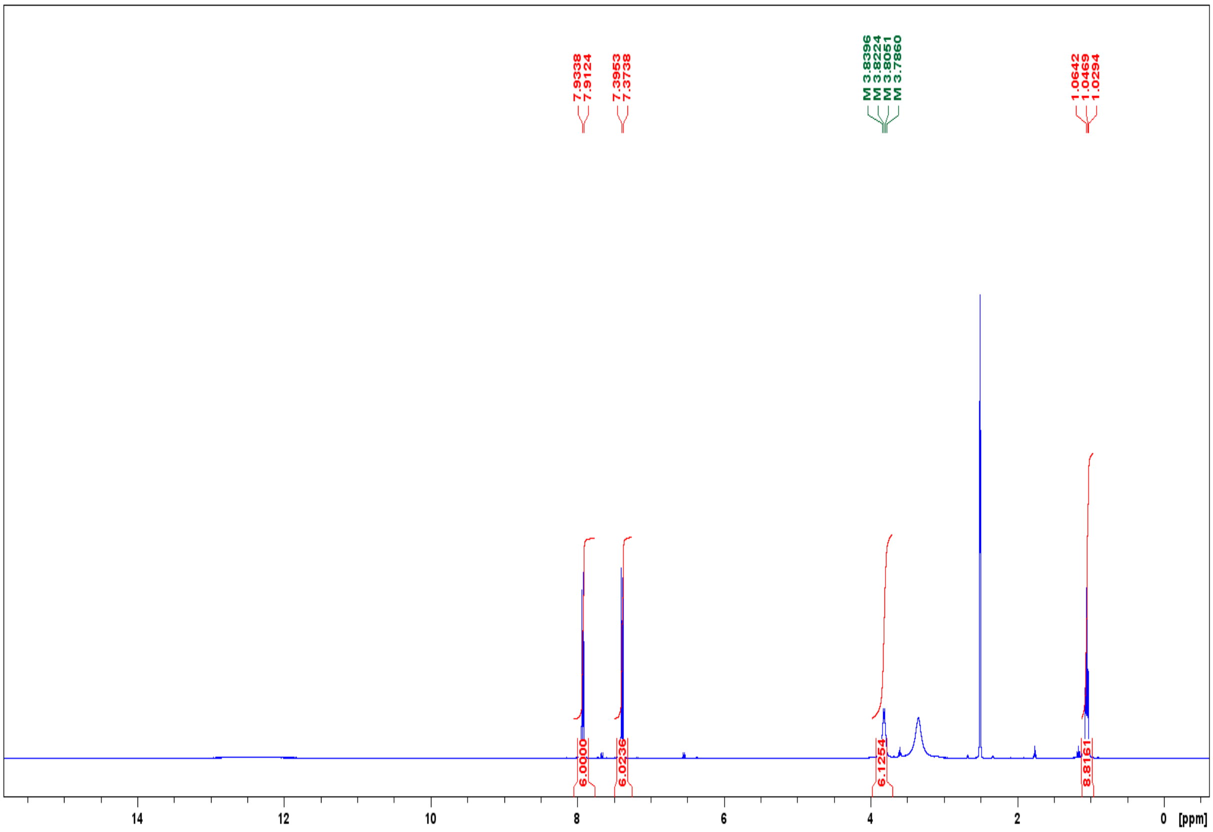

Figure S13. ^{1}H NMR spectrum of ligand **3e** in DMSO-d_6 at 373 K.

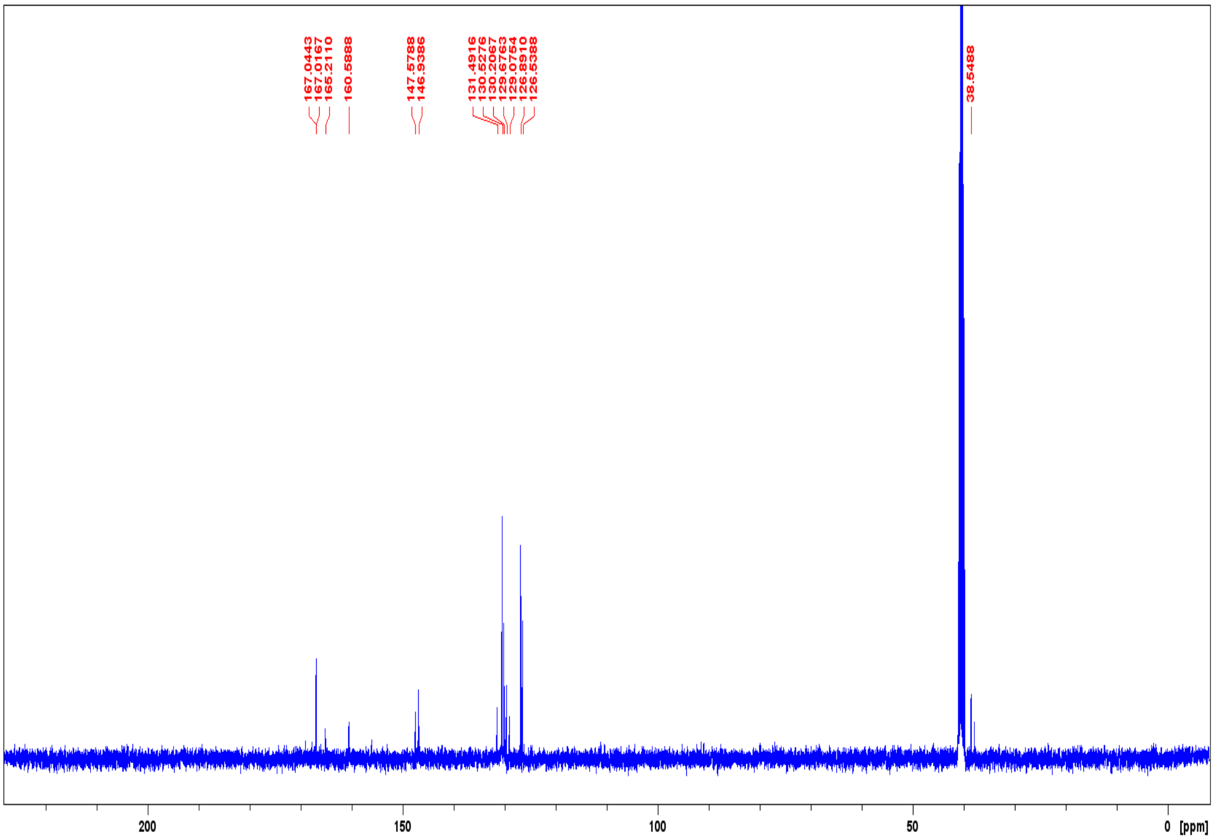

Figure S14. ^{13}C NMR spectrum of ligand **3e** in DMSO-d_6 at 373 K.

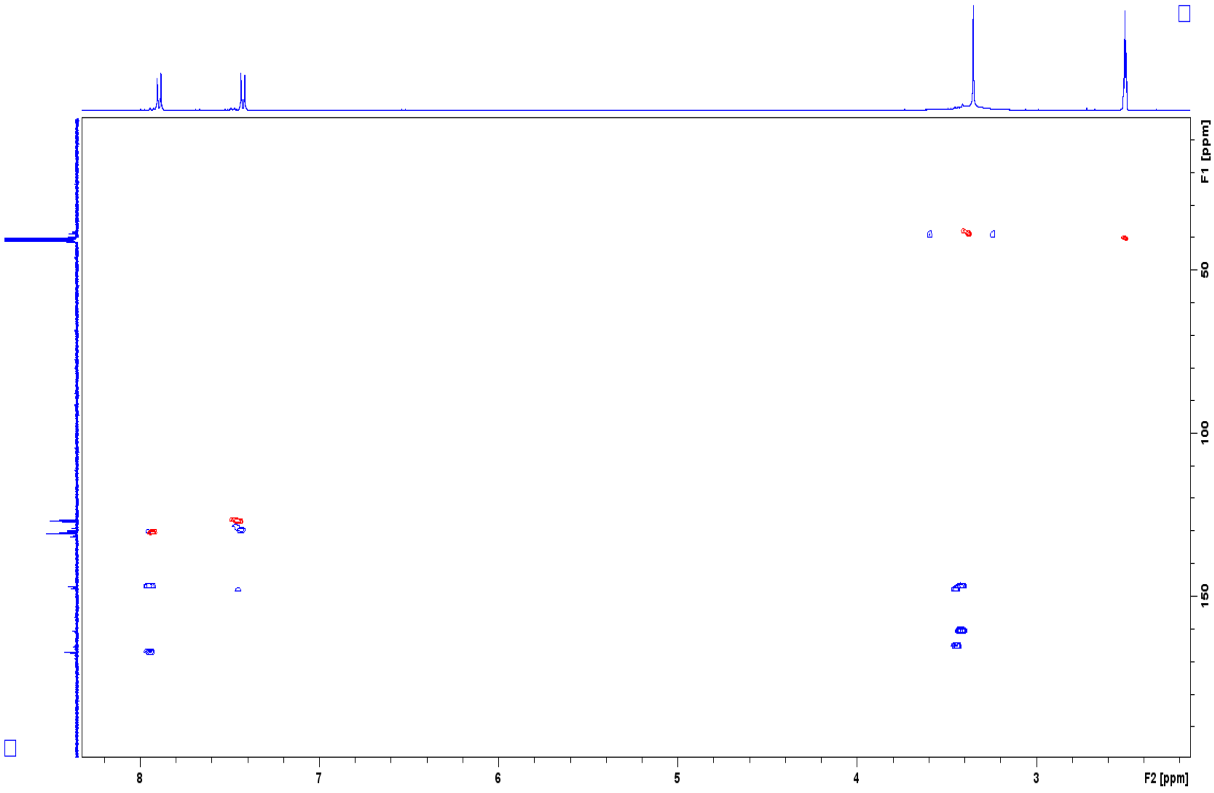

Figure S15. ^{1}H-^{13}C NMR experiments HSQC (red) and HMBC (blue) of ligand **3e** in DMSO-d_6 at 373 K.

<u>NMR spectra of compound **3f**</u>

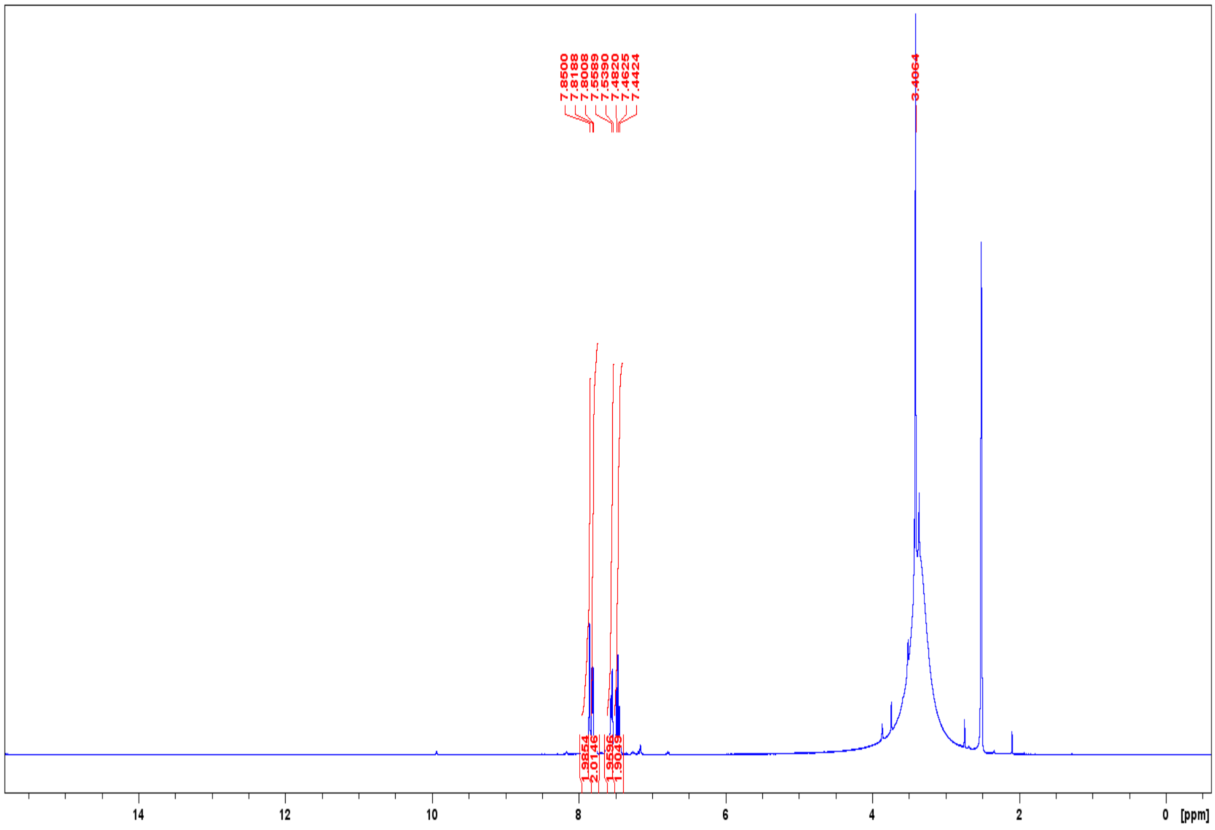

Figure S16. ^{1}H NMR spectrum of ligand **3f** in DMSO-d_6 at 373 K.

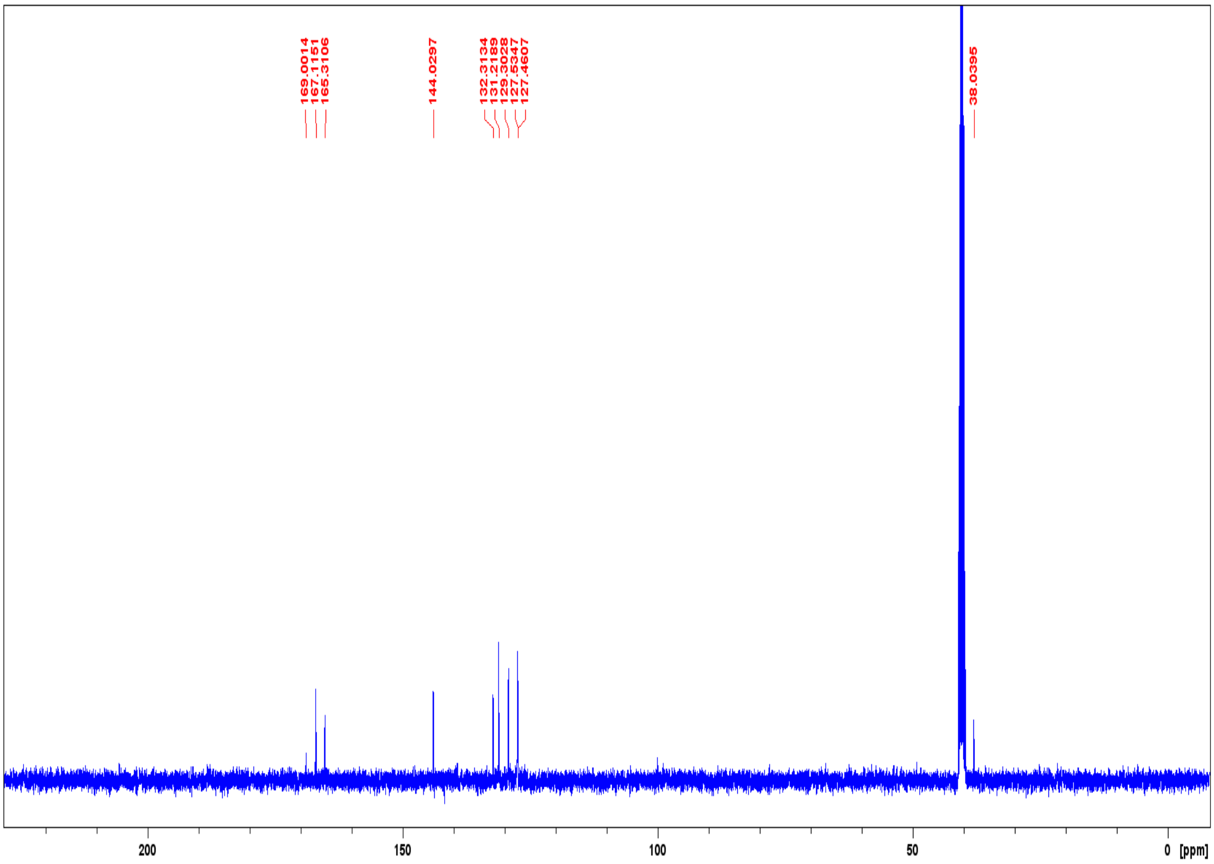

Figure S17. ^{13}C NMR spectrum of ligand **3f** in DMSO-d$_6$ at 373 K.

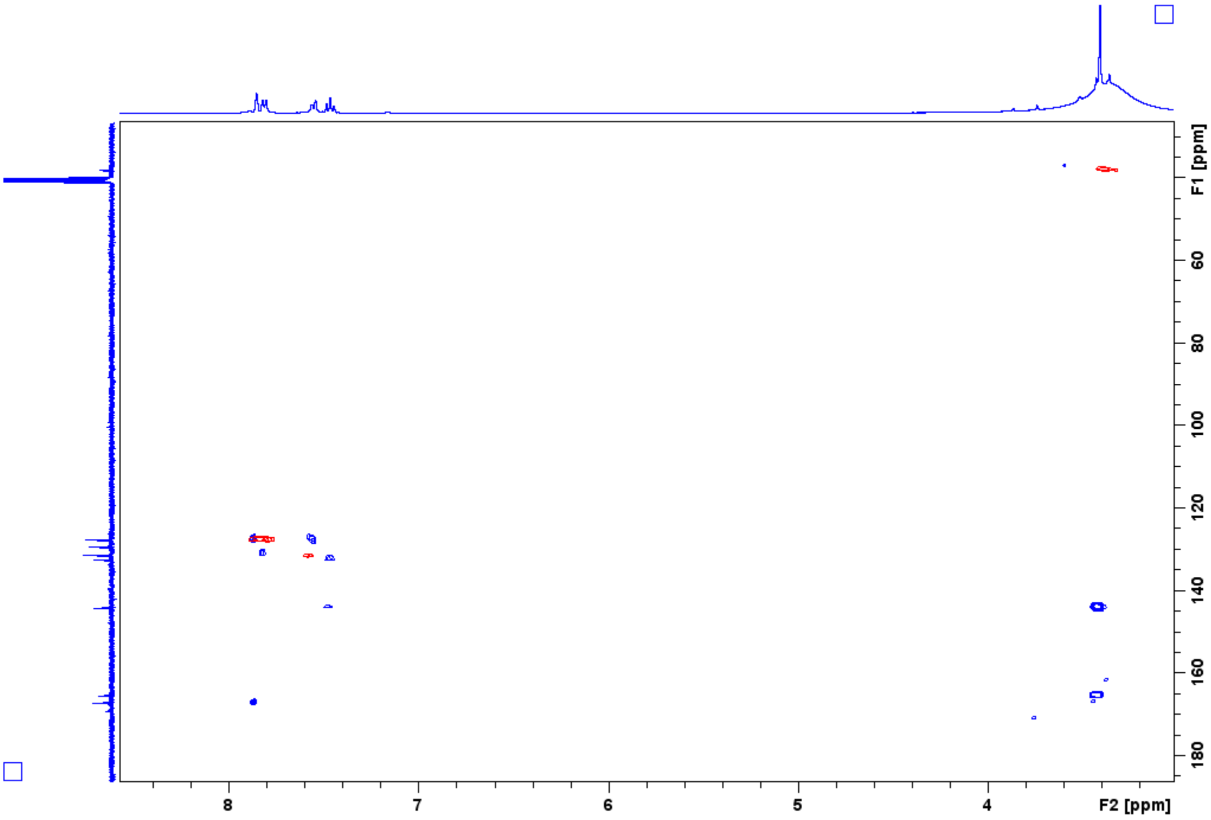

Figure S18. ^{1}H-^{13}C NMR experiments HSQC (red) and HMBC (blue) of ligand **3f** in DMSO-d$_6$ at 373 K.

NMR spectra of compound **3g**

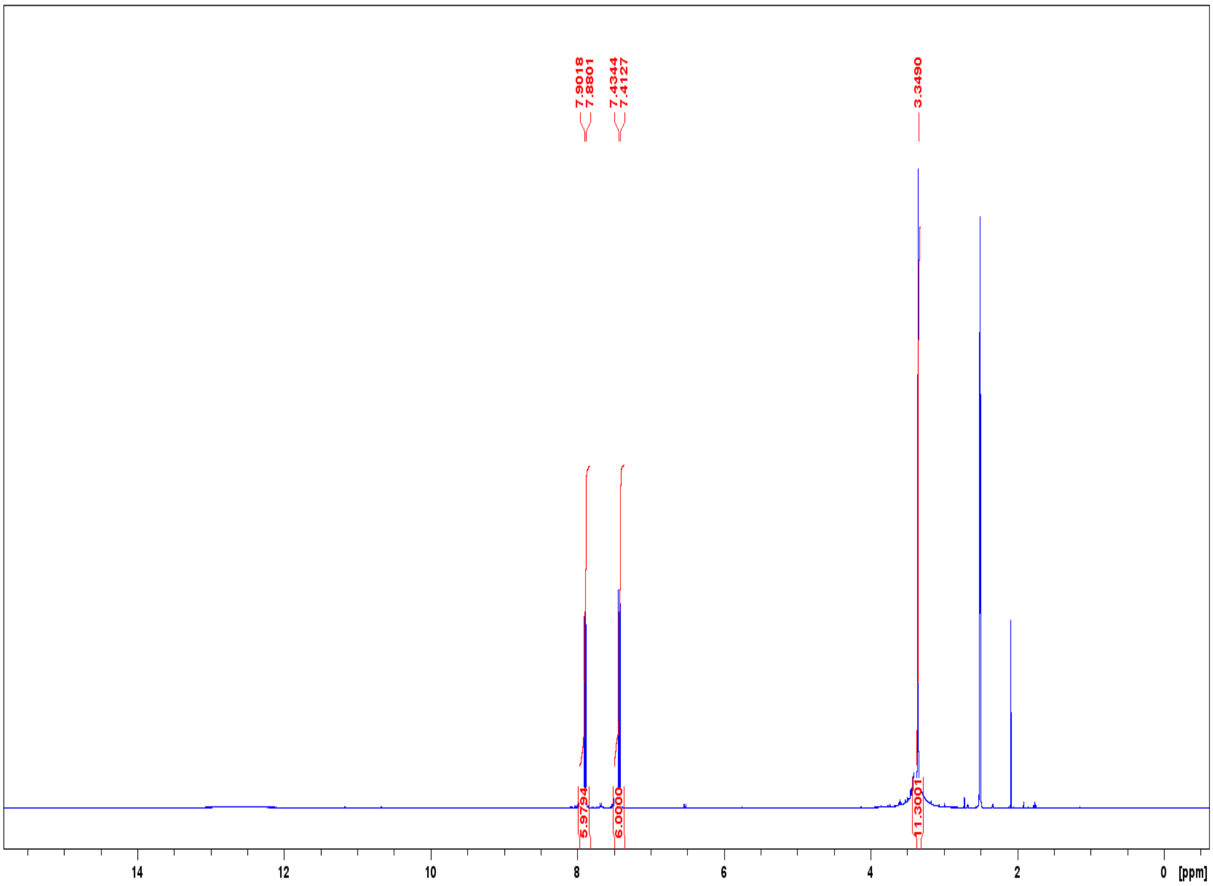

Figure S19. ^{1}H NMR spectrum of ligand **3g** in DMSO-d$_6$ at 300 K.

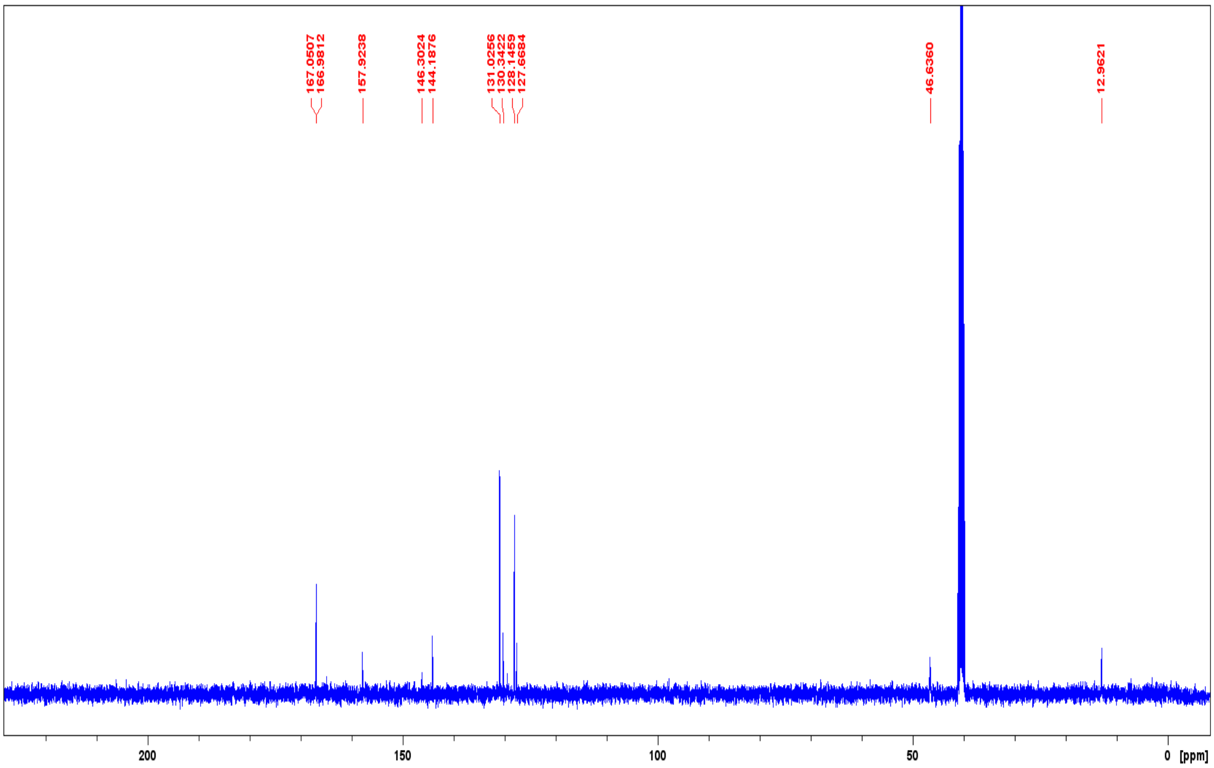

Figure S20. ^{13}C NMR spectrum of ligand **3g** in DMSO-d$_6$ at 373 K.

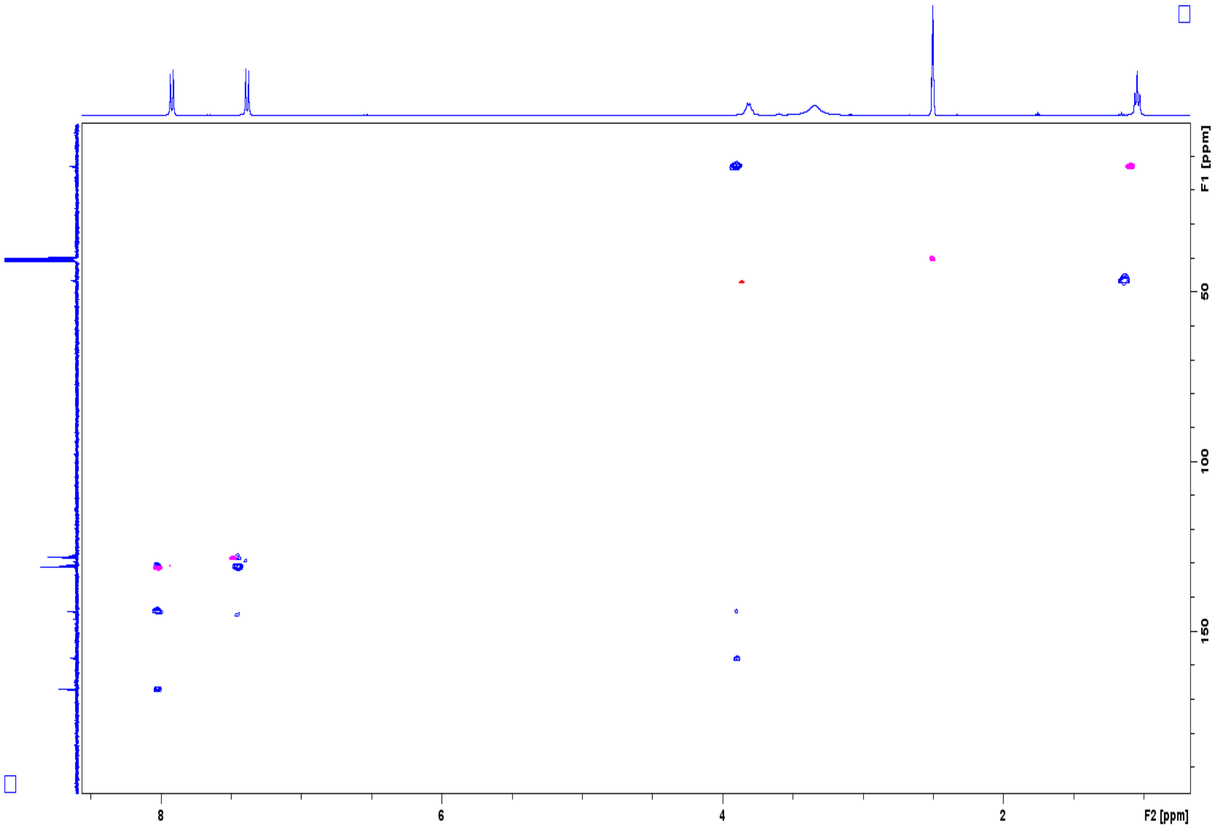

Figure S21. ^{1}H-^{13}C NMR experiments HSQC (red) and HMBC (blue) of ligand **3g** in DMSO-d$_6$ at 373 K.

<u>NMR spectra of compound **3h**</u>

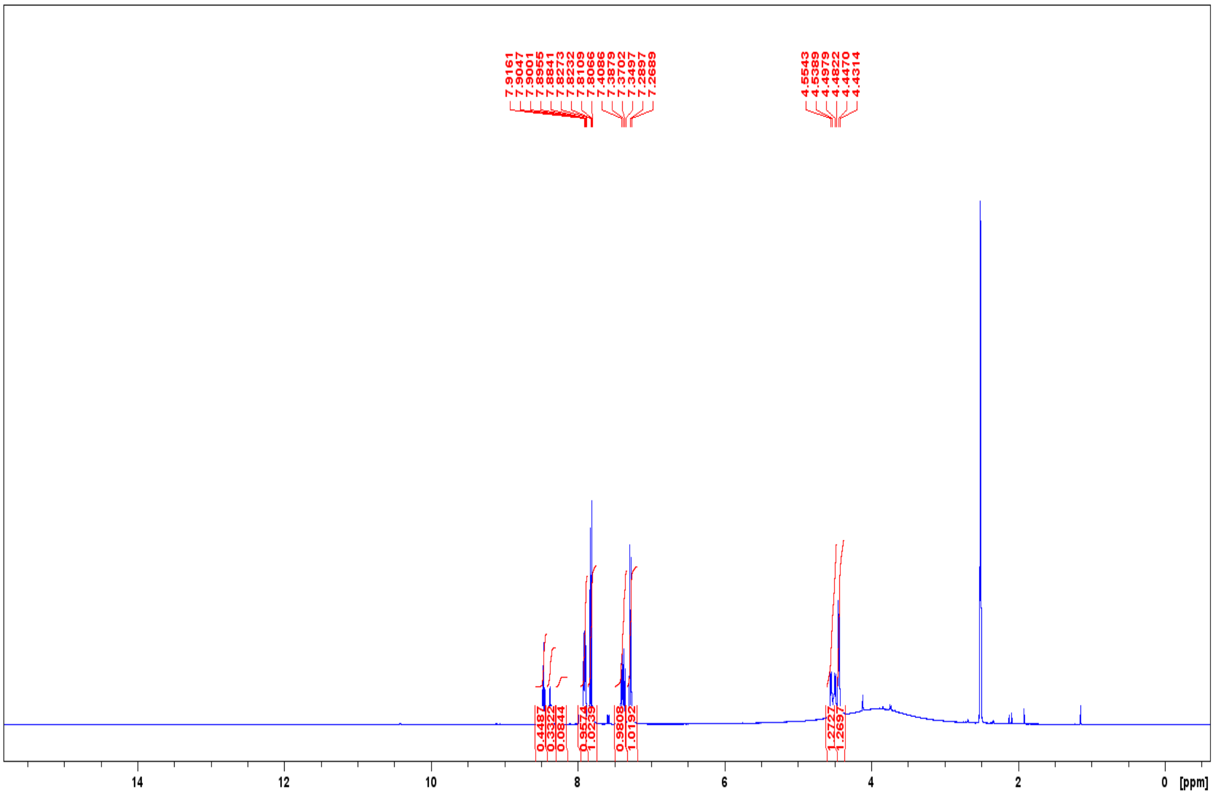

Figure S22. ^{1}H NMR spectrum of ligand **3h** in DMSO-d$_6$. at 300 K

c

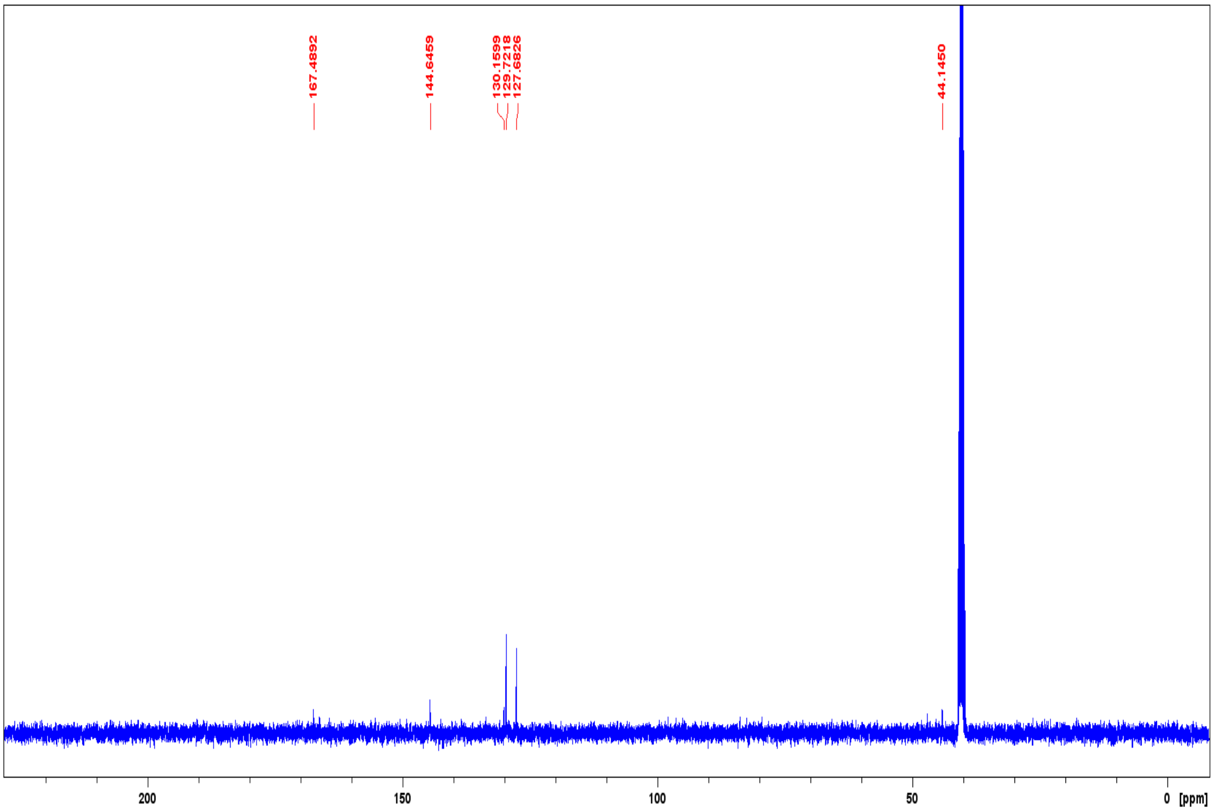

Figure S23. ^{13}C NMR spectrum of ligand **3h** in DMSO-d6 at 373 K.

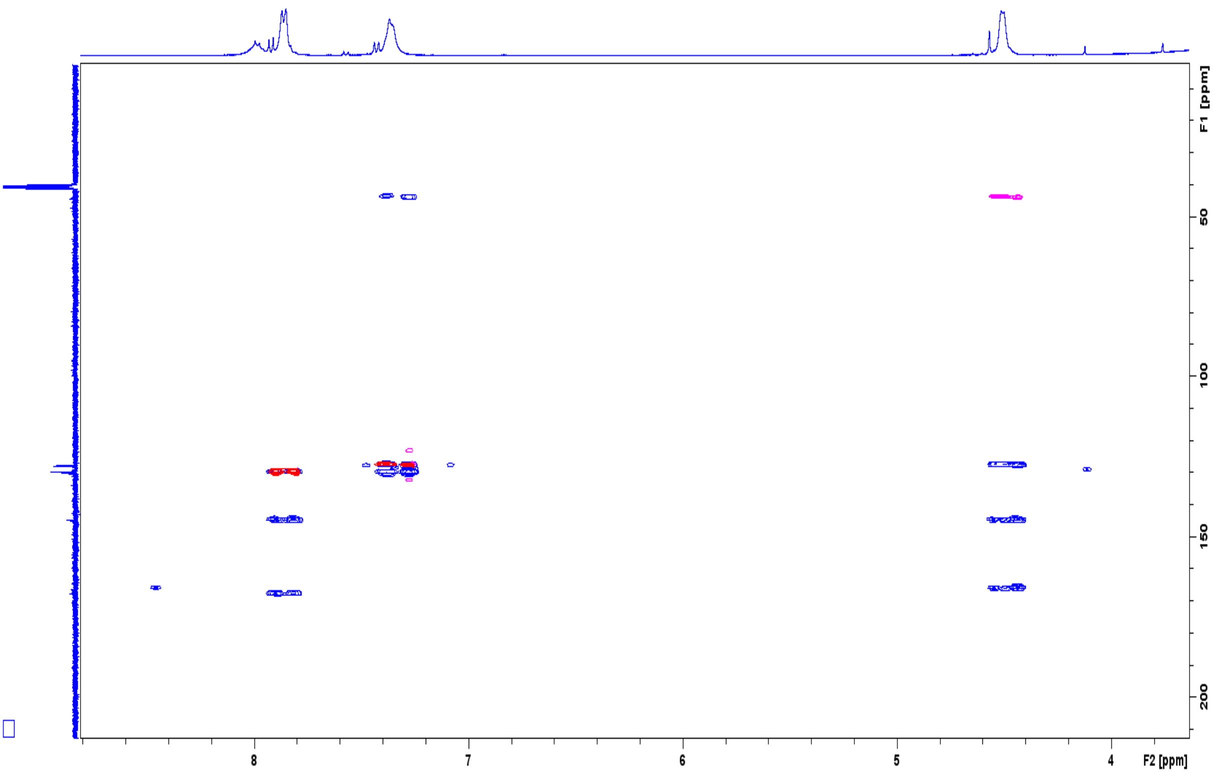

Figure S24. ^{1}H-^{13}C NMR experiments HSQC (red) and HMBC (blue) of ligand **3h** in DMSO-d6 at 373 K.

NMR spectra of compound **3i**

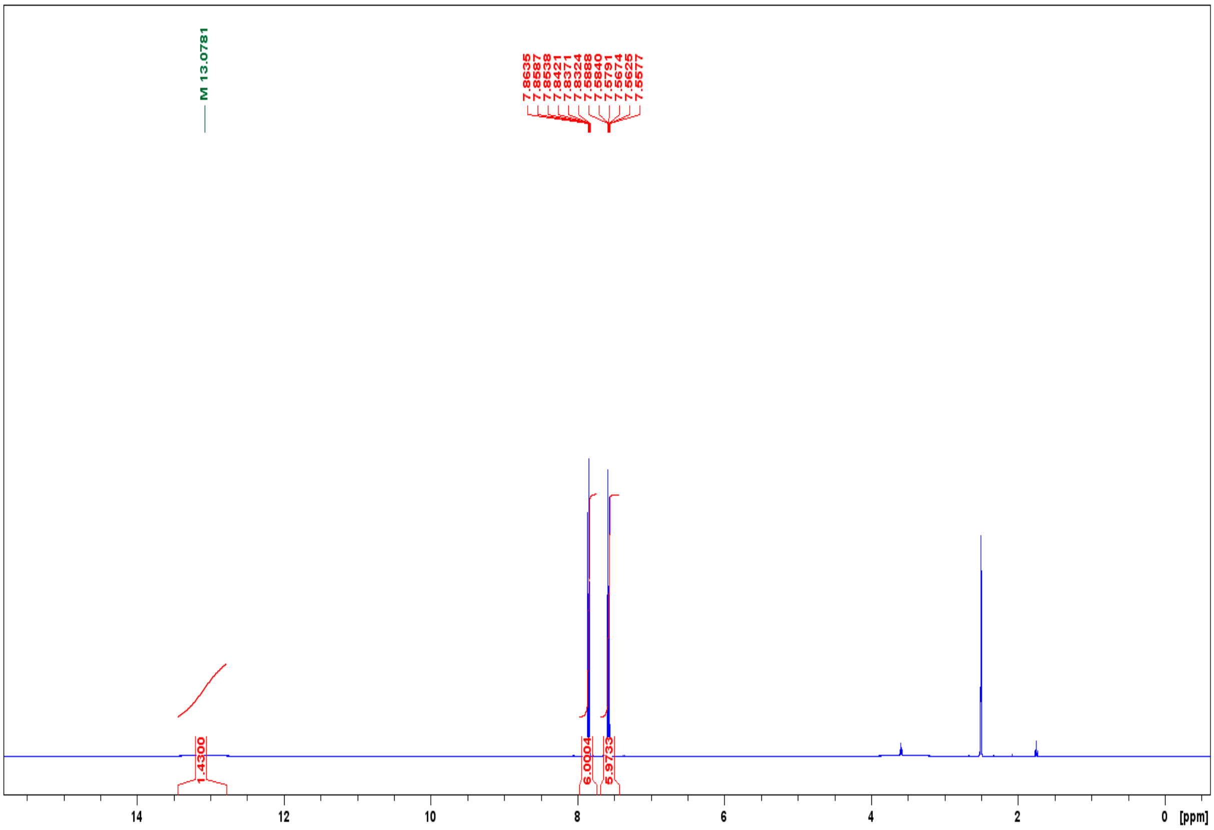

Figure S25. ^{1}H NMR spectrum of ligand **3i** in DMSO-d6.

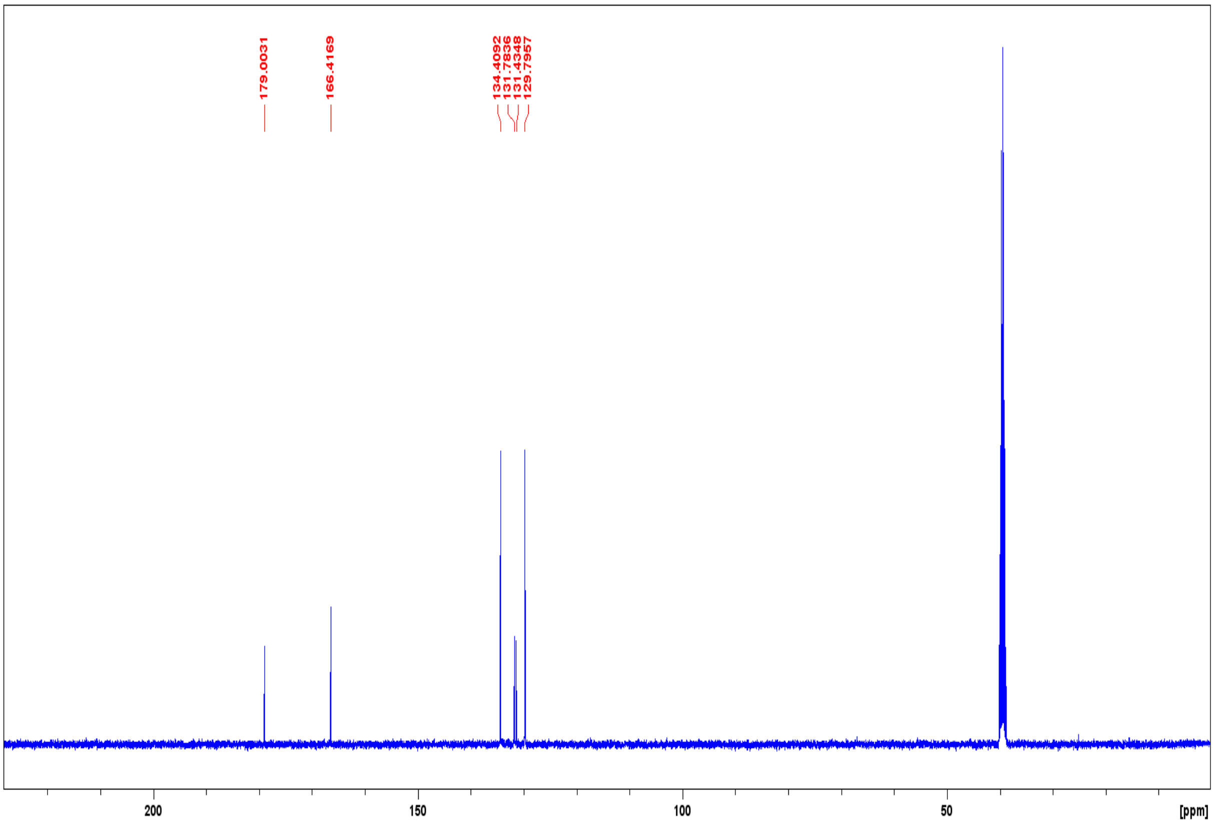

Figure S26. ^{13}C NMR spectrum of ligand **3i** in DMSO-d6.

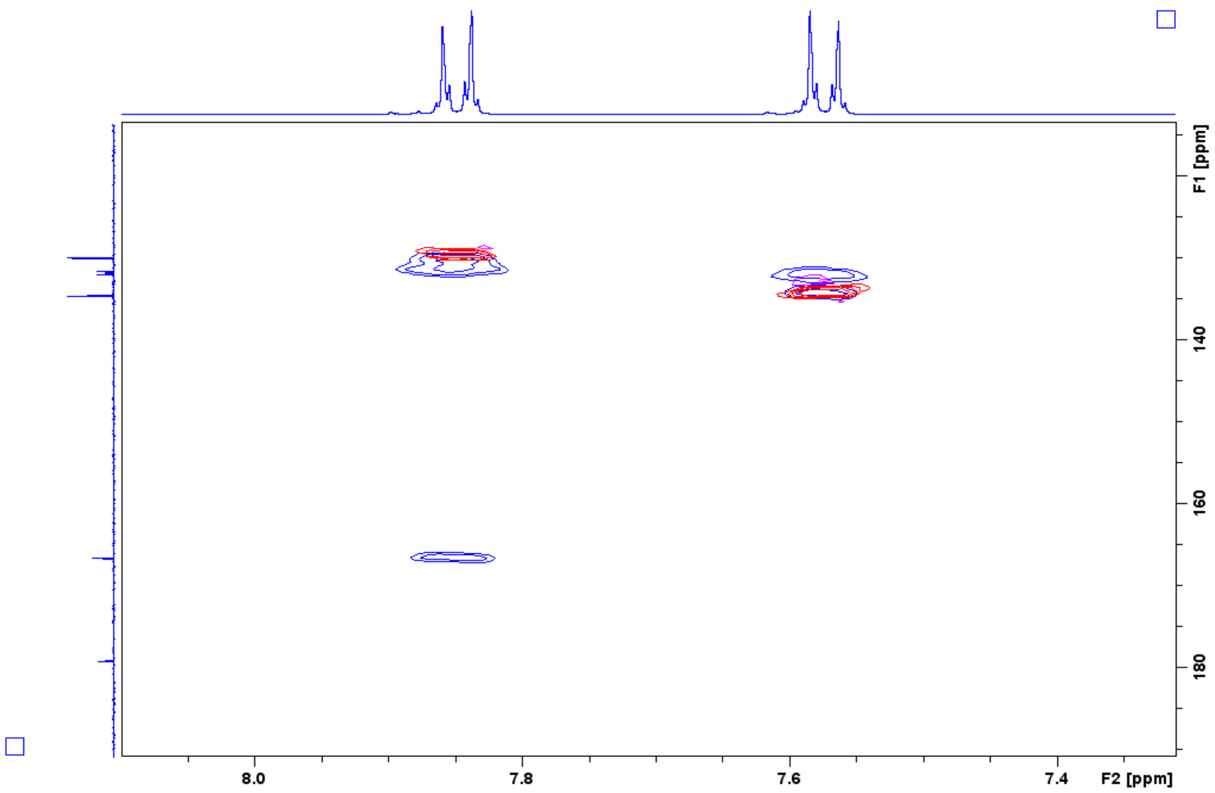

Figure S27. ^{1}H-^{13}C NMR experiments HSQC (red) and HMBC (blue) of ligand **3i** in DMSO-d_6.

NMR spectra of compound **3j**

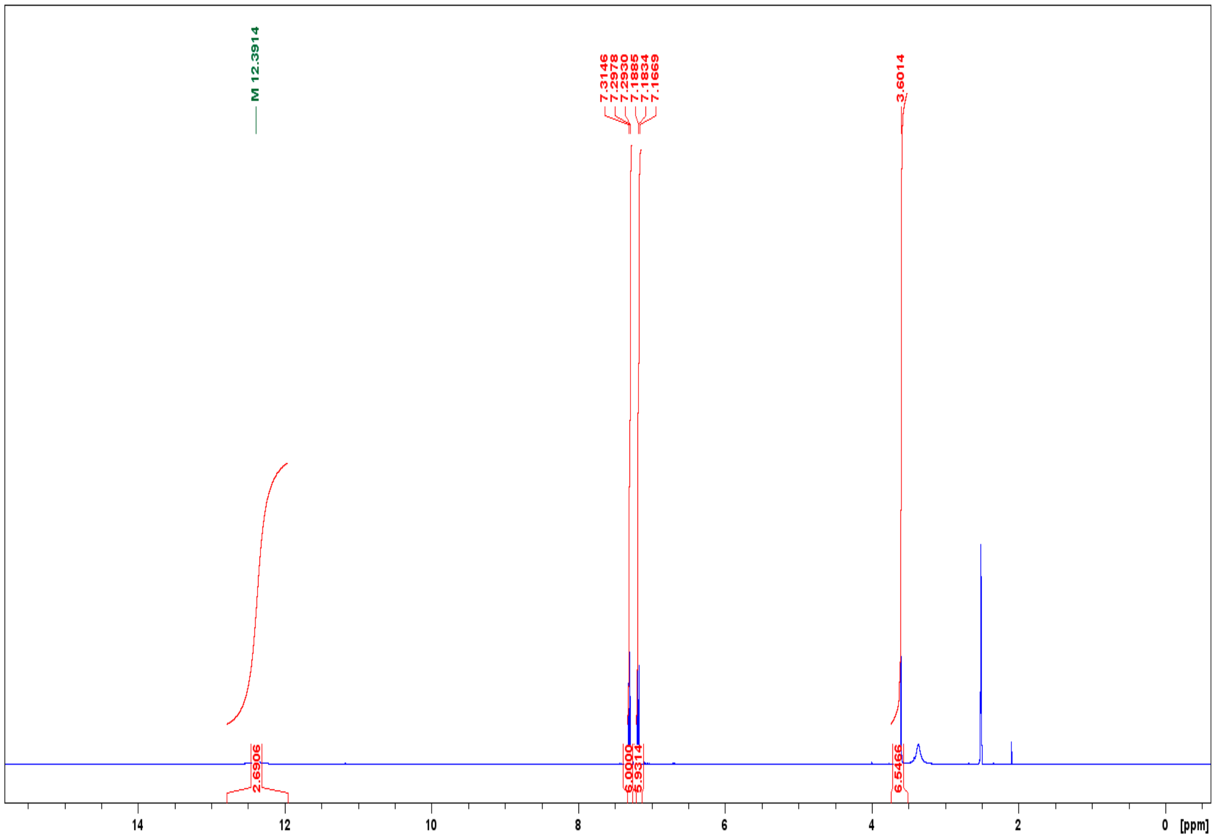

Figure S28. ^{1}H NMR spectrum of ligand **3j** in DMSO-d_6.

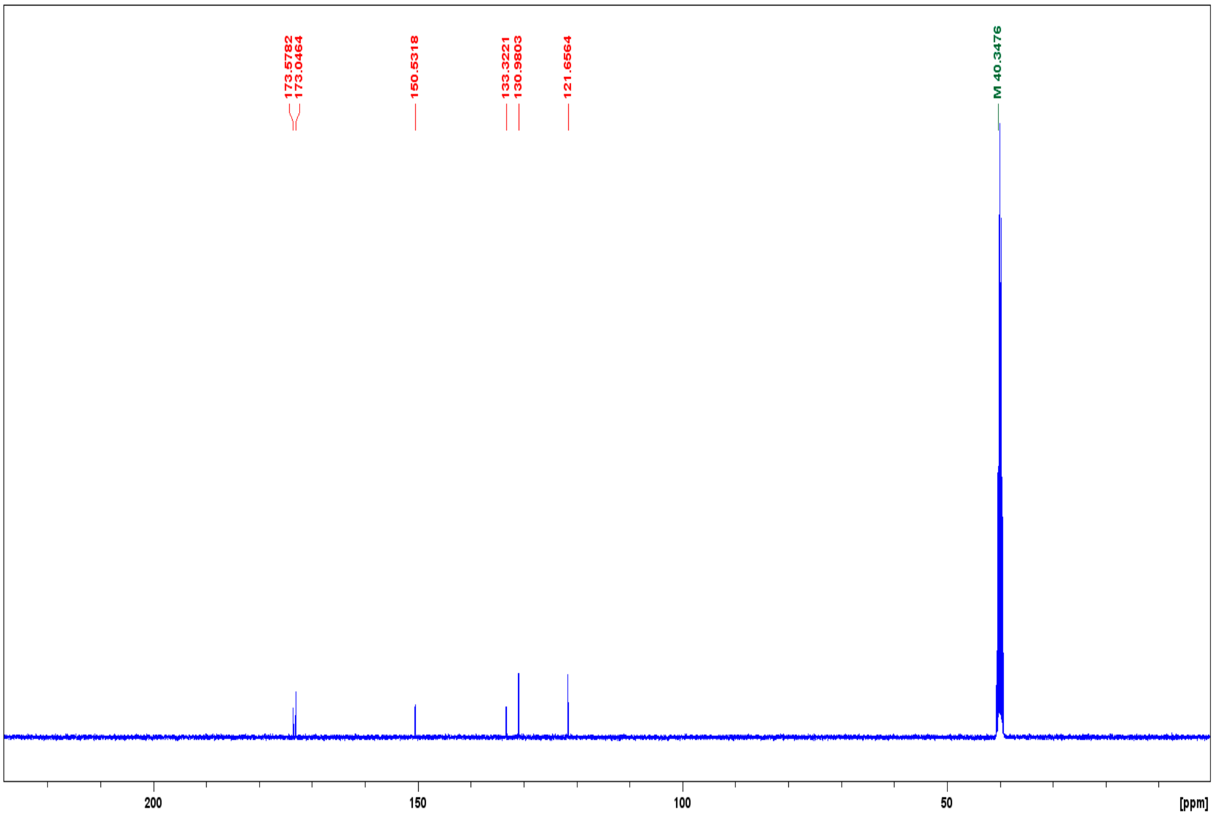

Figure S29. ^{13}C NMR spectrum of ligand **3j** in DMSO-d6.

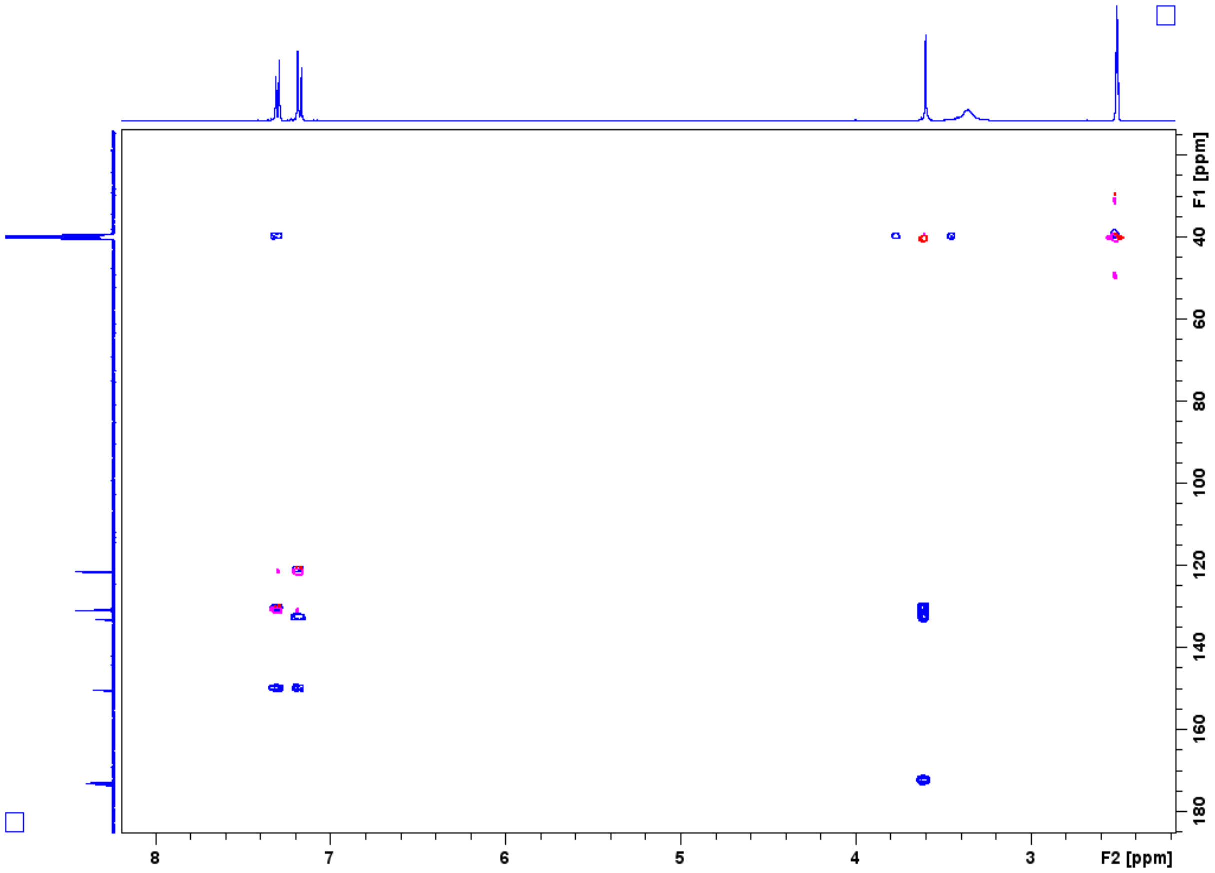

Figure S30. ^{1}H-^{13}C NMR experiments HSQC (red) and HMBC (blue) of ligand **3j** in DMSO-d6.

NMR spectra of compound **3k**

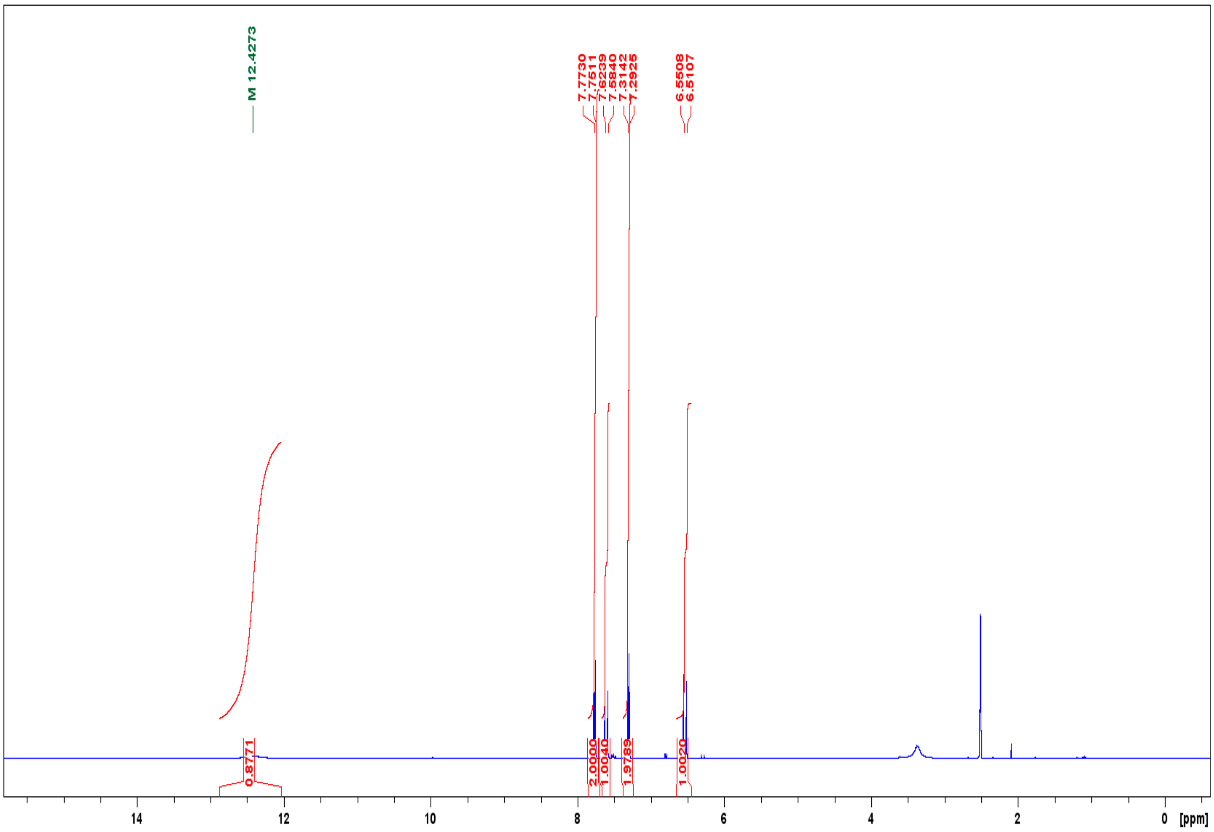

Figure S31. ^{1}H NMR spectrum of ligand **3k** in DMSO-d_6.

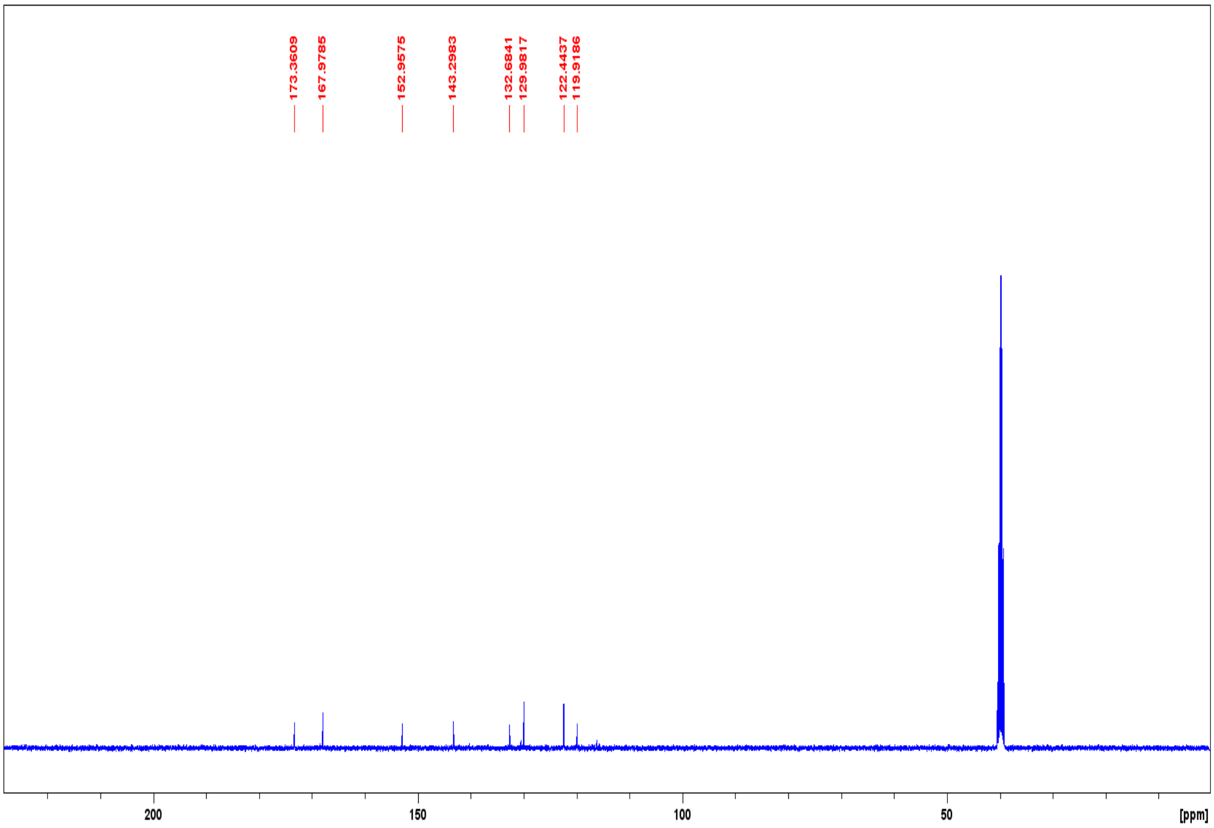

Figure S32. ^{13}C NMR spectrum of ligand **3k** in DMSO-d_6.

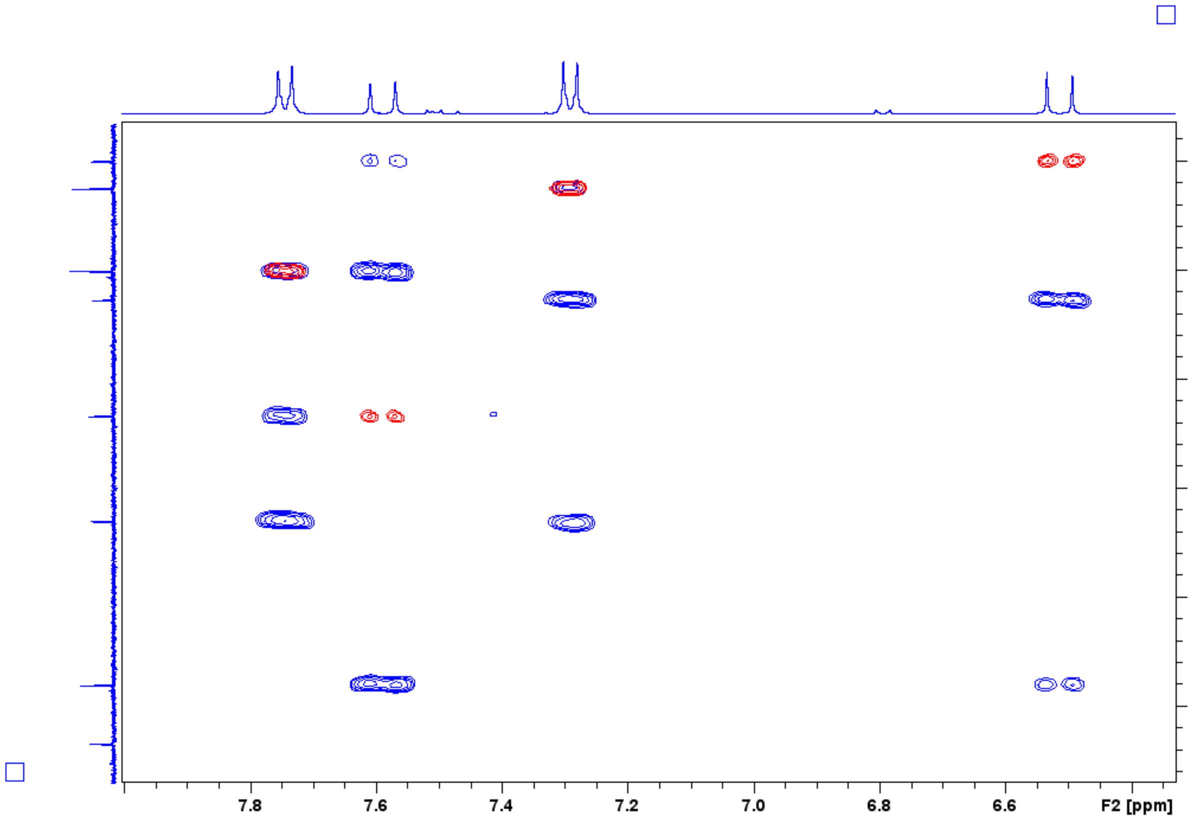

Figure S33. ^{1}H-^{13}C NMR experiments HSQC (red) and HMBC (blue) of ligand **3k** in DMSO-d6.

NMR spectra of compound **3l**

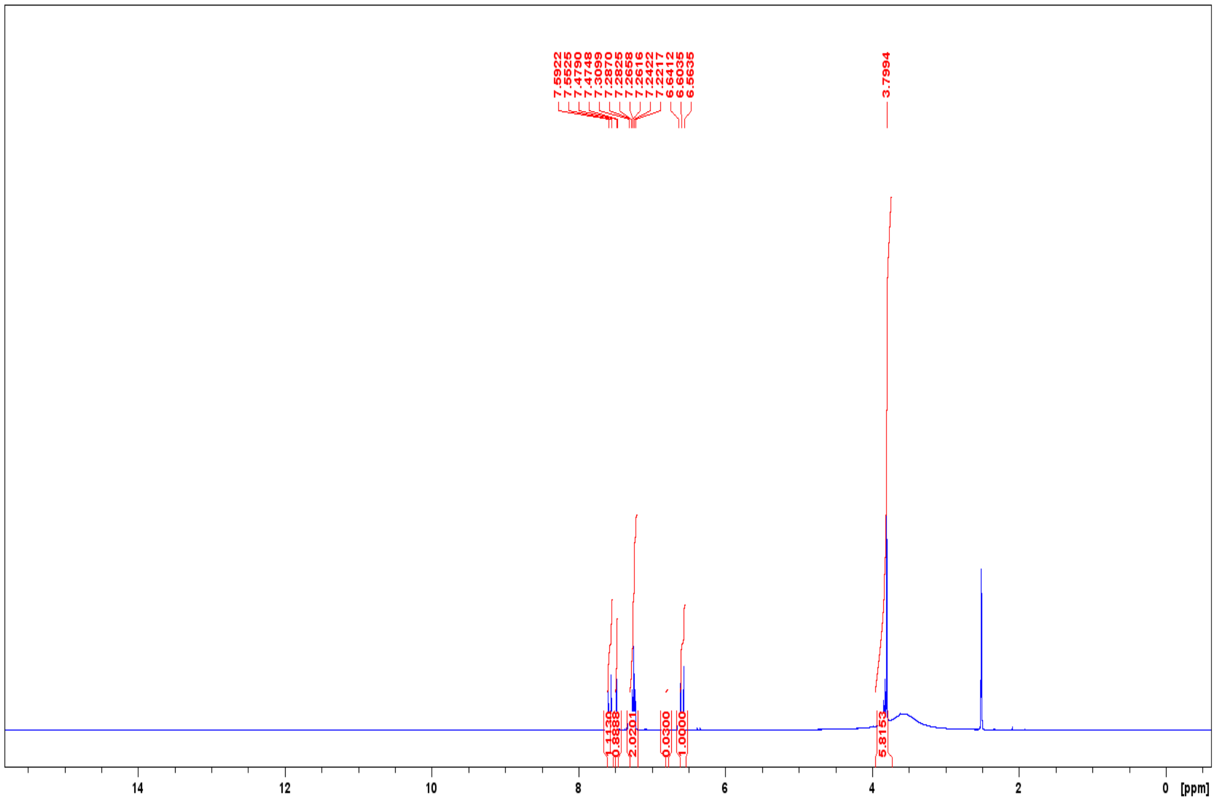

Figure S34. ^{1}H NMR spectrum of ligand **3l** in DMSO-d6.

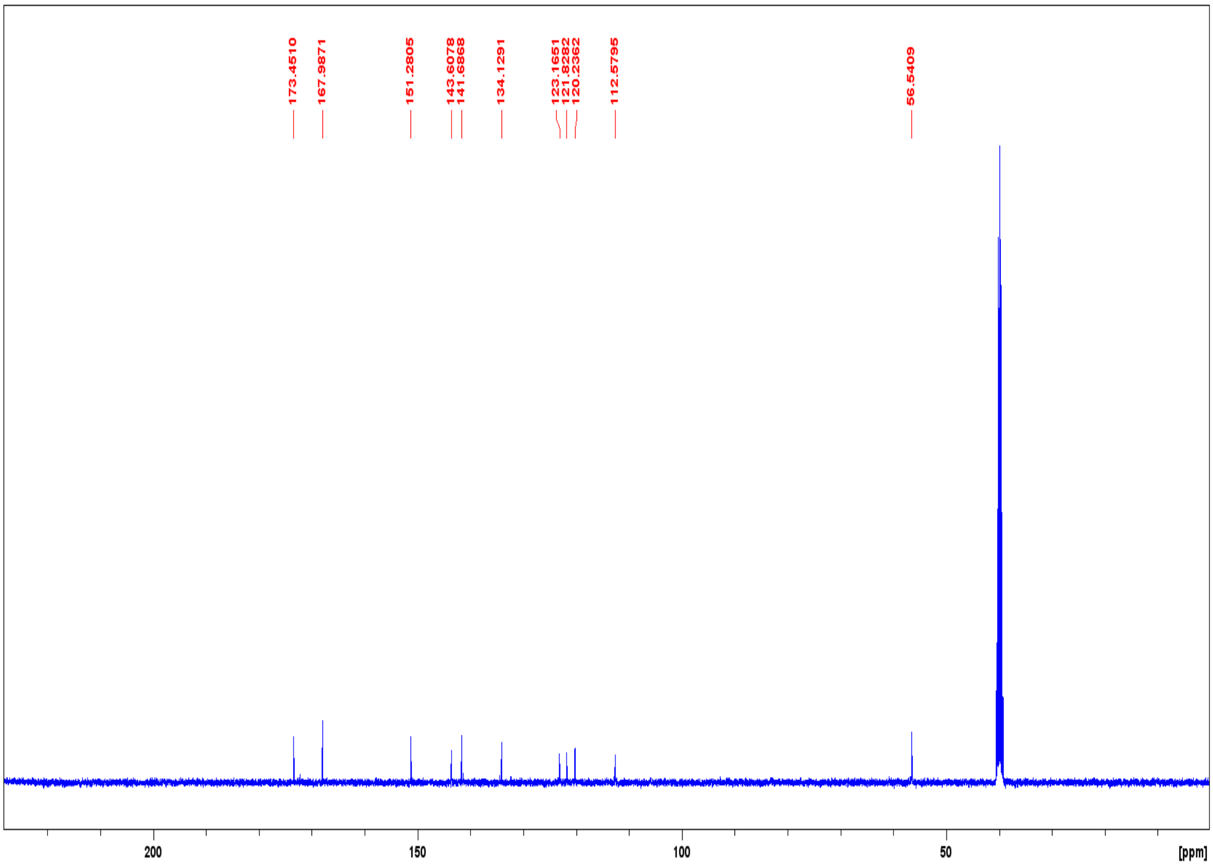

Figure S35. ^{13}C NMR spectrum of ligand **3l** in DMSO-d6.

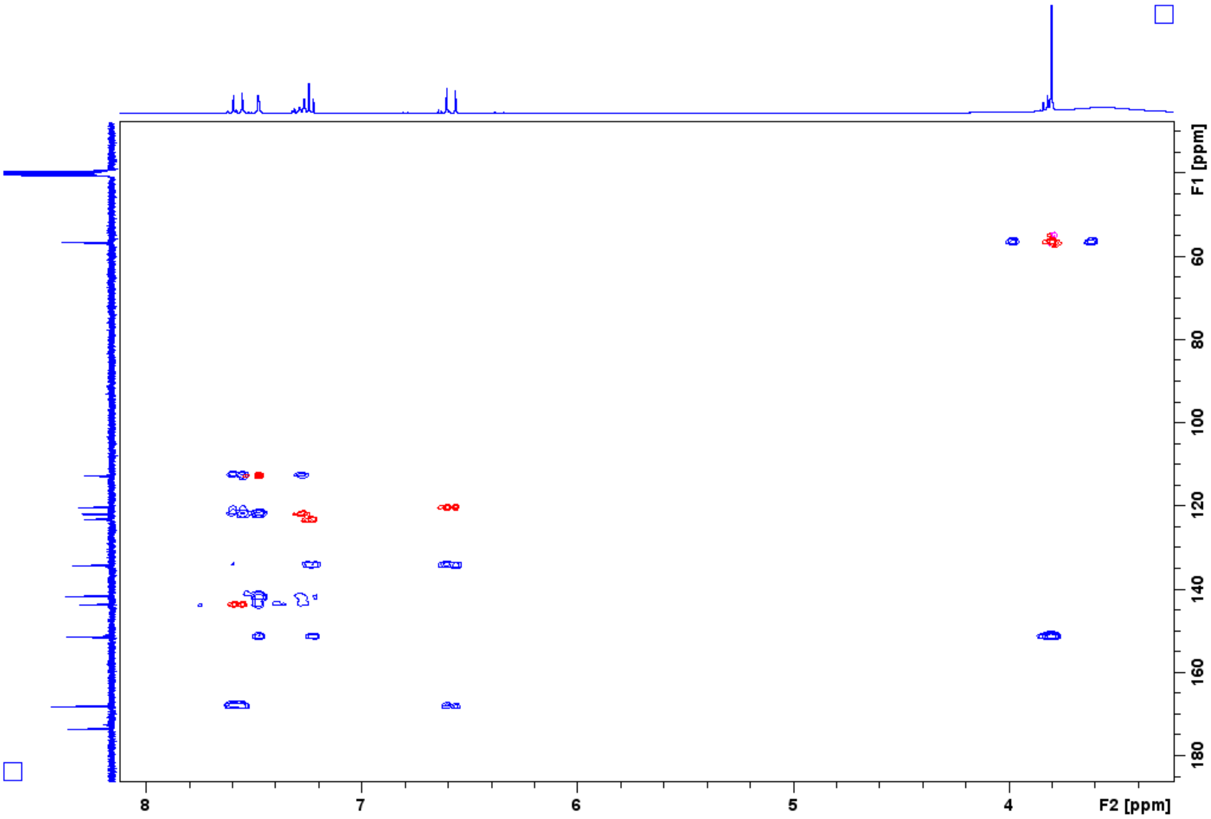

Figure S36. ^{1}H-^{13}C NMR experiments HSQC (red) and HMBC (blue) of ligand **3l** in DMSO-d6.

<u>NMR spectra of compound **3m**</u>

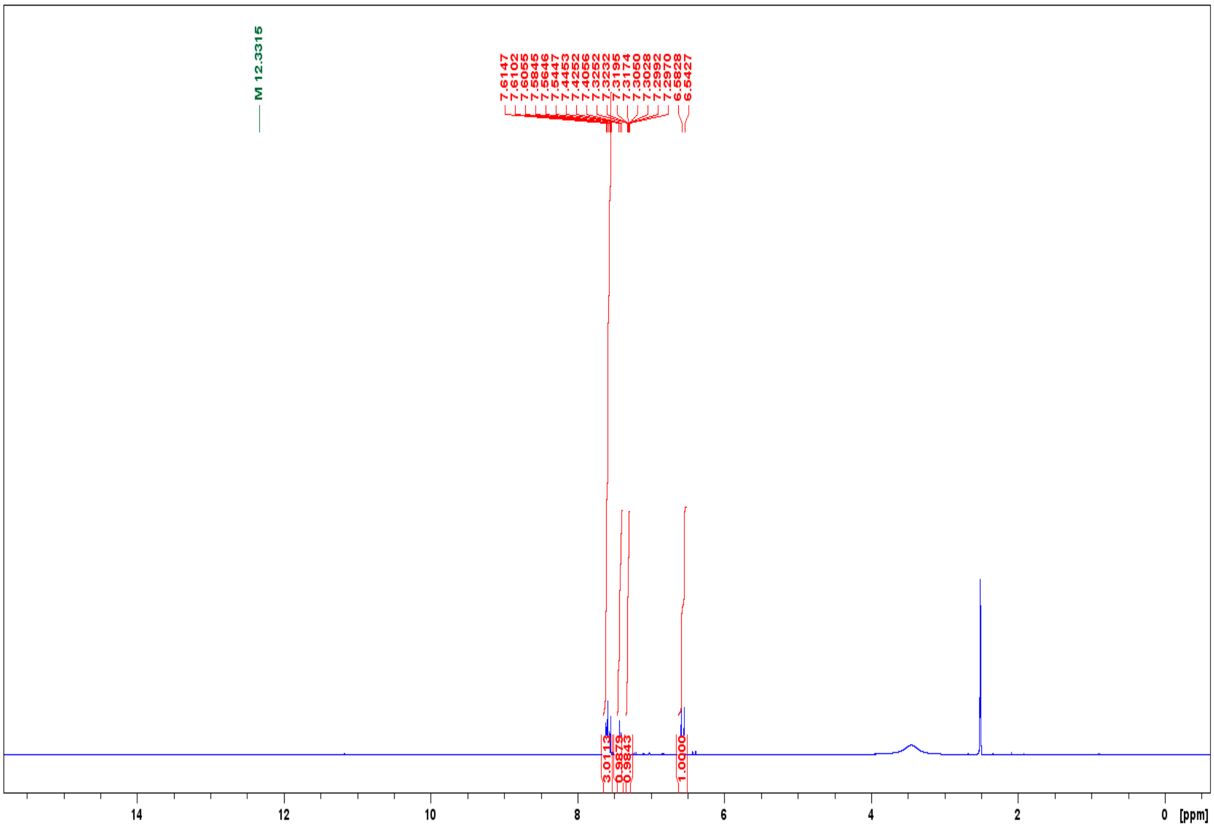

Figure S37. ^{1}H NMR spectrum of ligand **3m** in DMSO-d6.

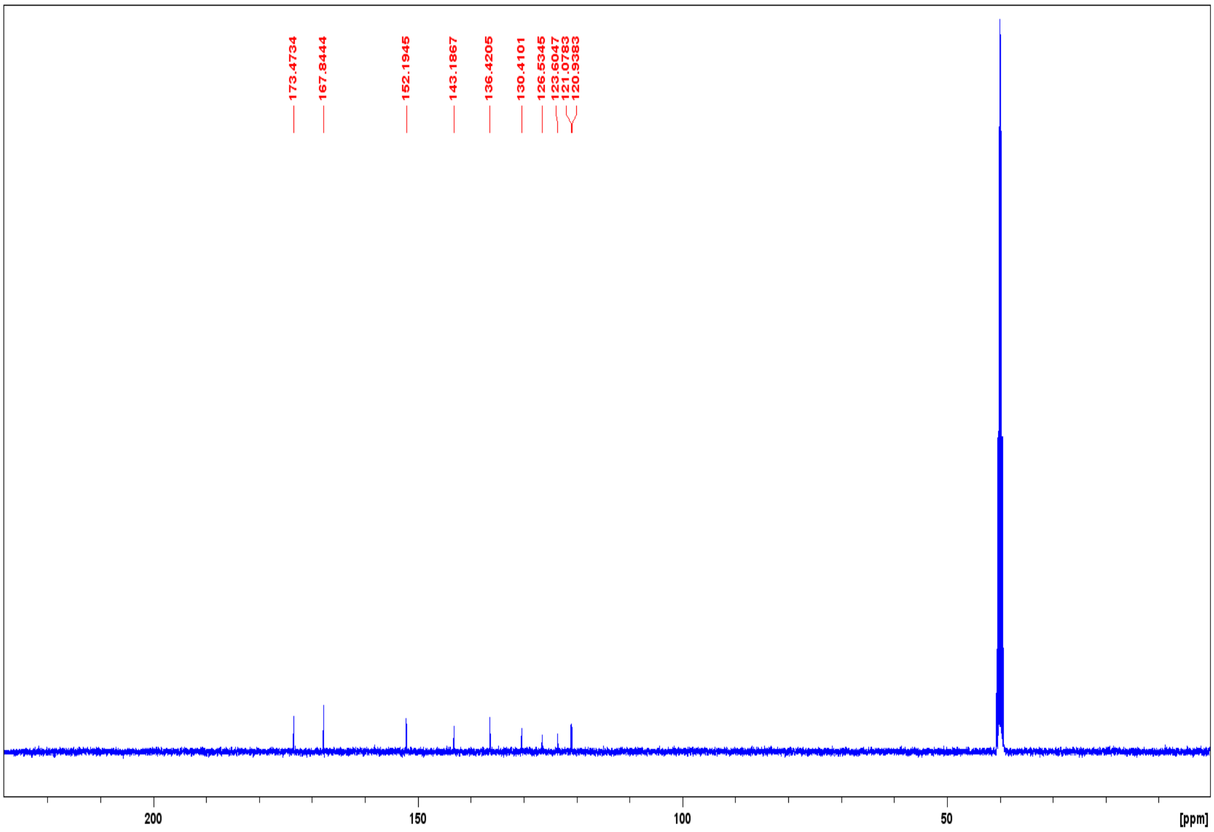

Figure S38. ^{13}C NMR spectrum of ligand **3m** in DMSO-d6.

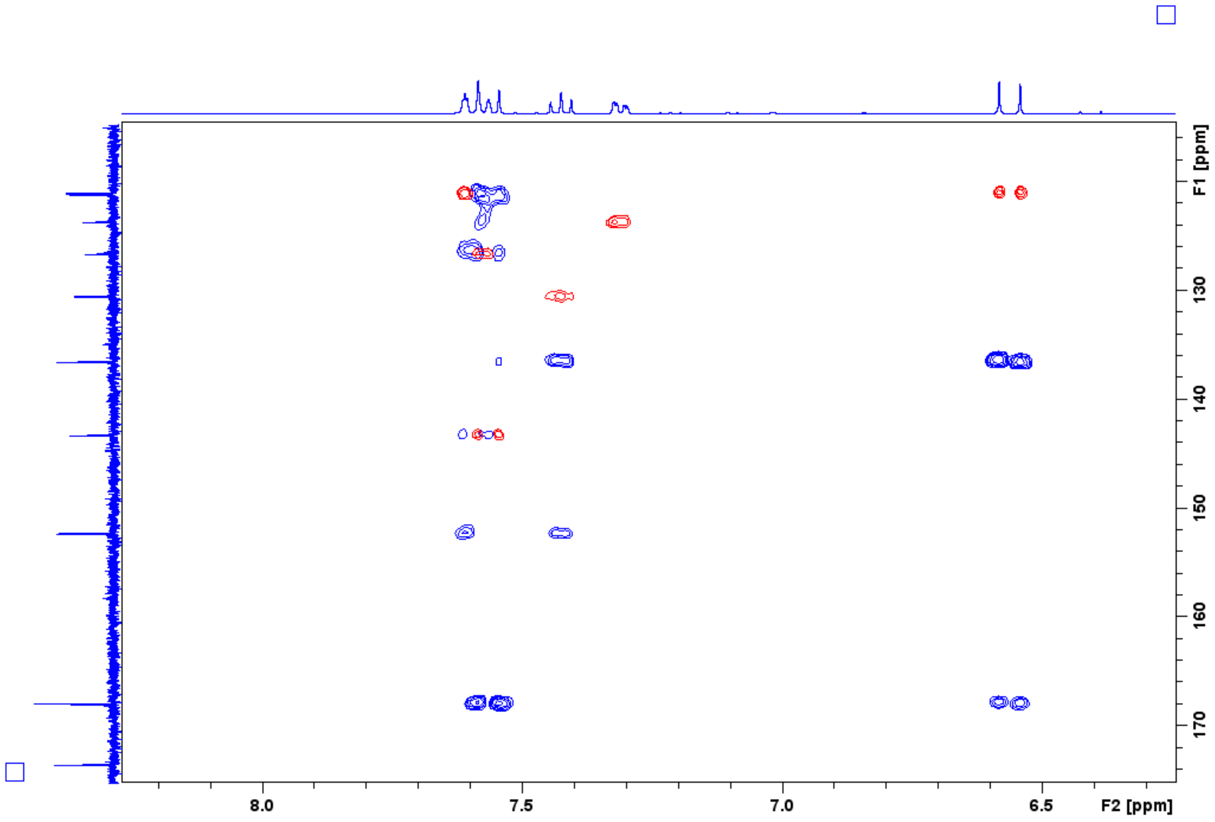

Figure S39. ^{1}H-^{13}C NMR experiments HSQC (red) and HMBC (blue) of ligand **3m** in DMSO-d6.

<u>NMR spectra of compound **3n**</u>

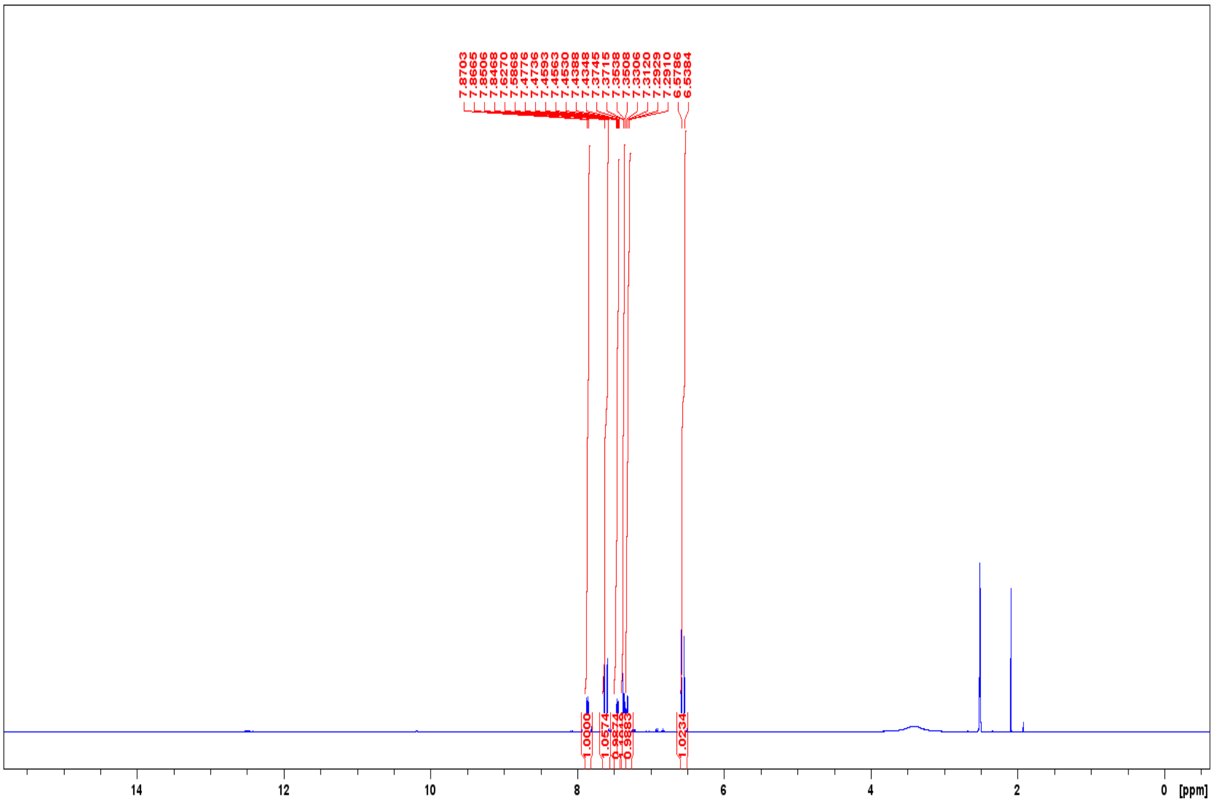

Figure S40. ^{1}H NMR spectrum of ligand **3n** in DMSO-d6.

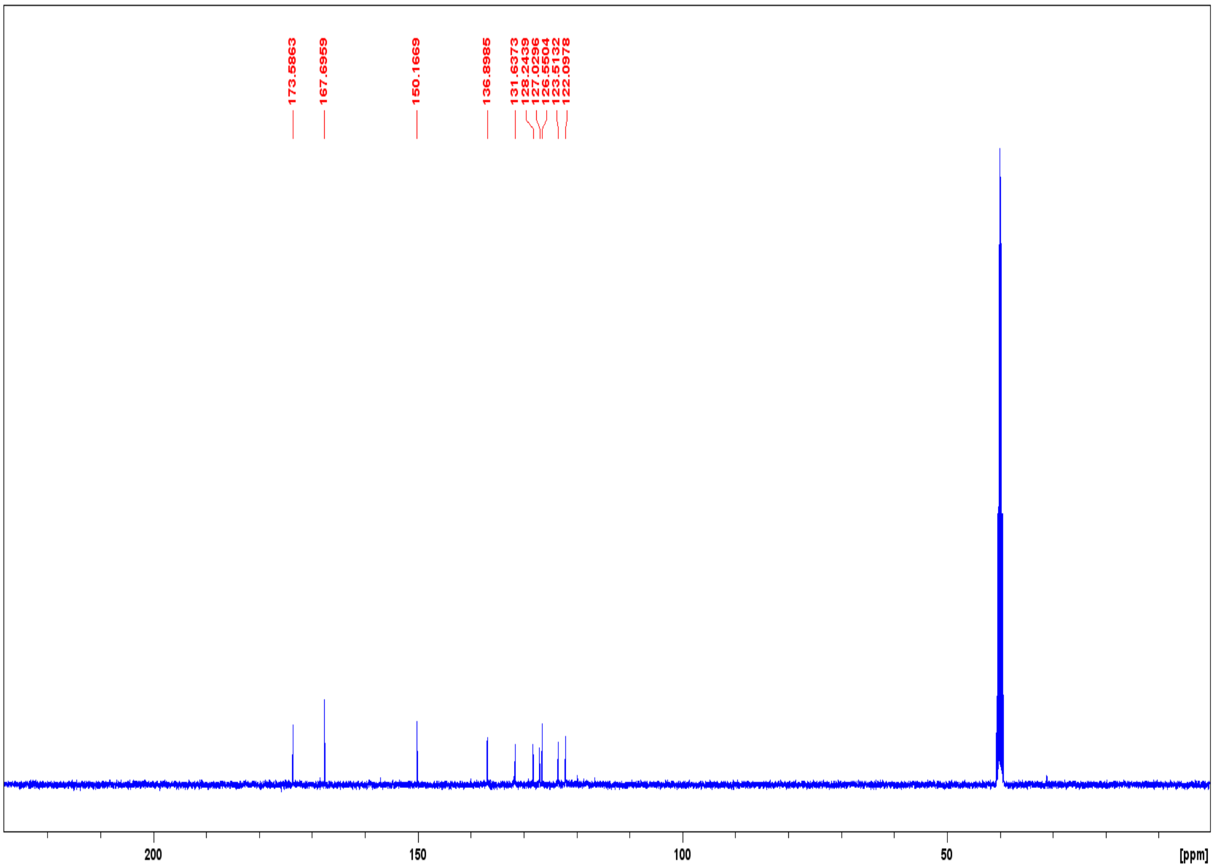

Figure S41. ^{13}C NMR spectrum of ligand **3n** in DMSO-d6.

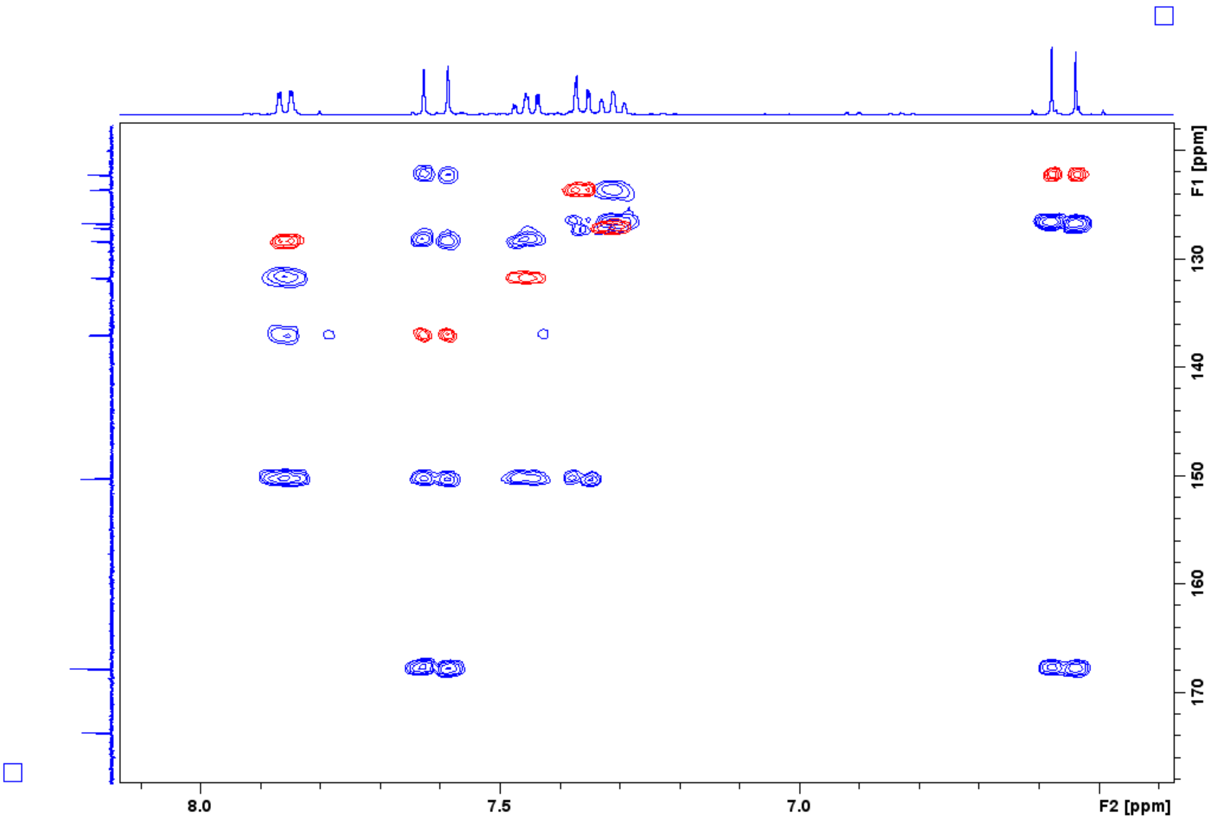

Figure S42. ^{1}H-^{13}C NMR experiments HSQC (red) and HMBC (blue) of ligand **3n** in DMSO-d6.

NMR spectra of compound **3o**

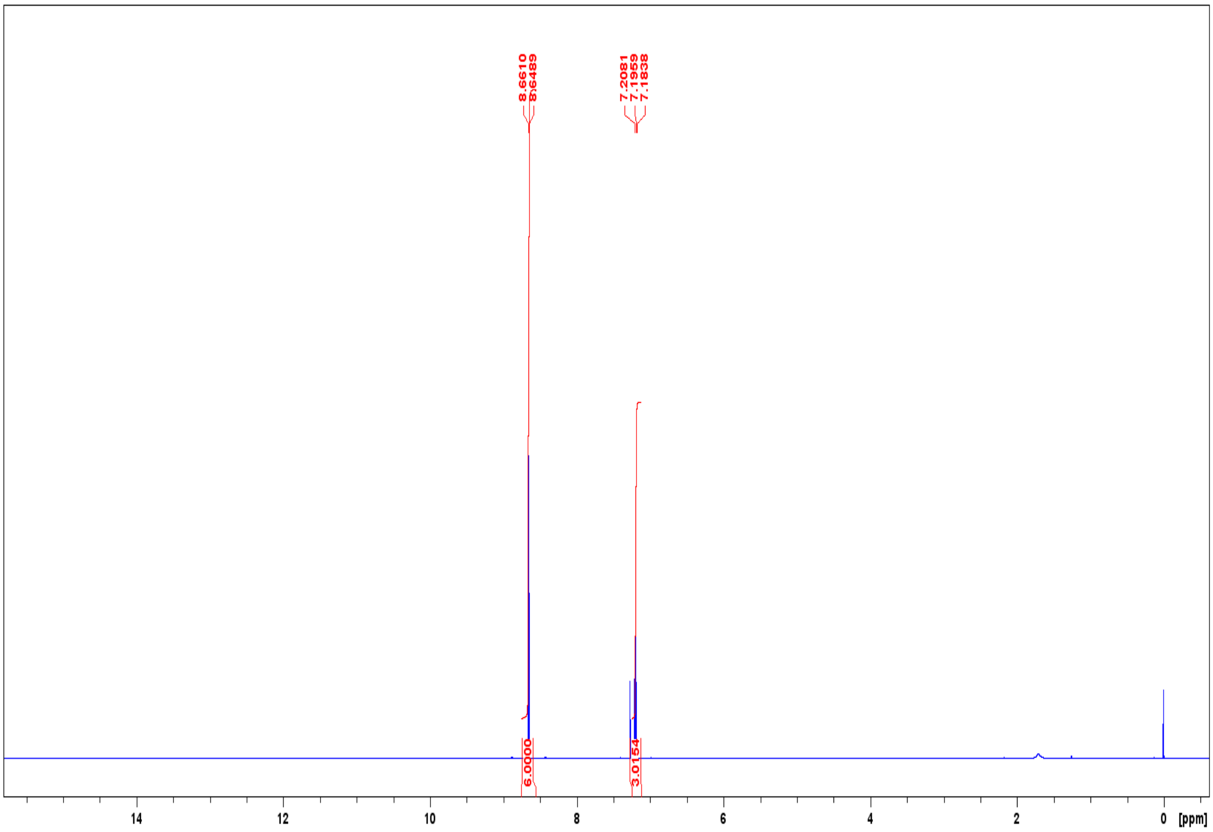

Figure S43. ^{1}H NMR spectrum of ligand **3o** in $CDCl_3$.

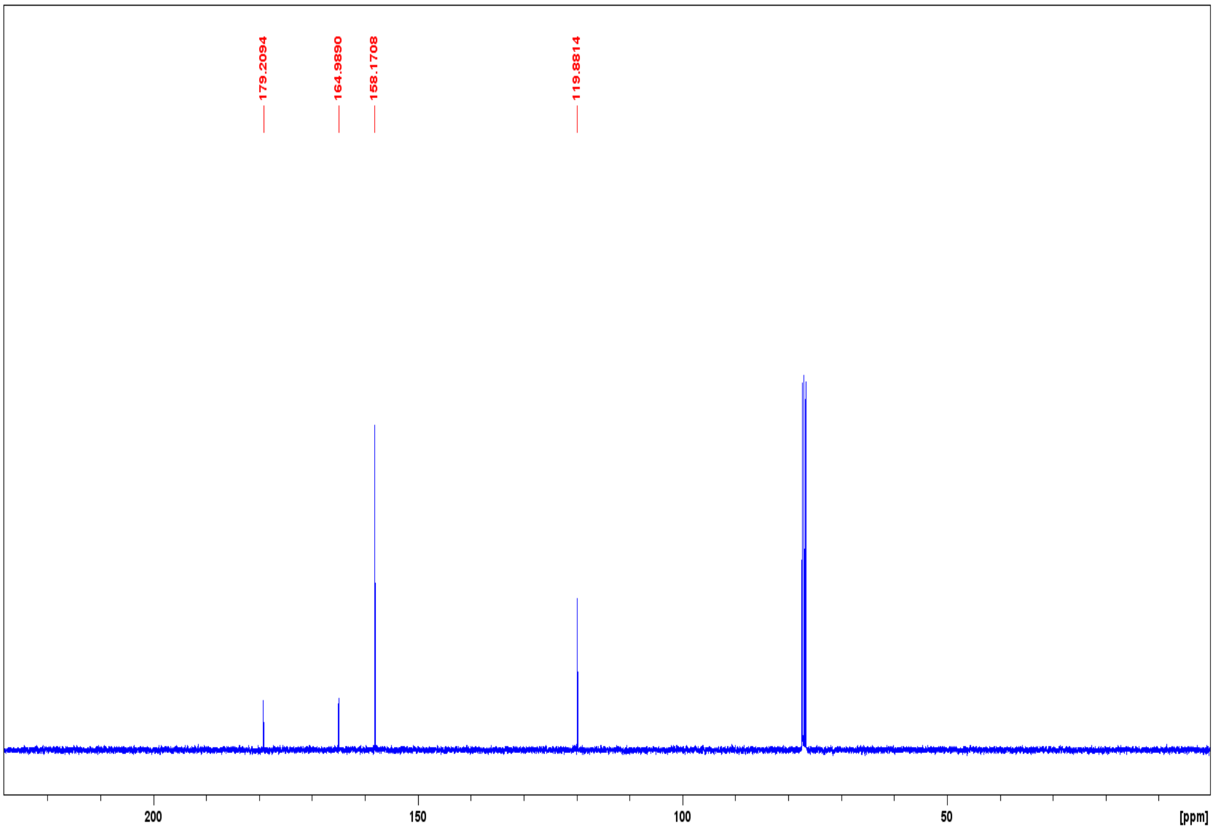

Figure S44. ^{13}C NMR spectrum of ligand **3o** in $CDCl_3$.

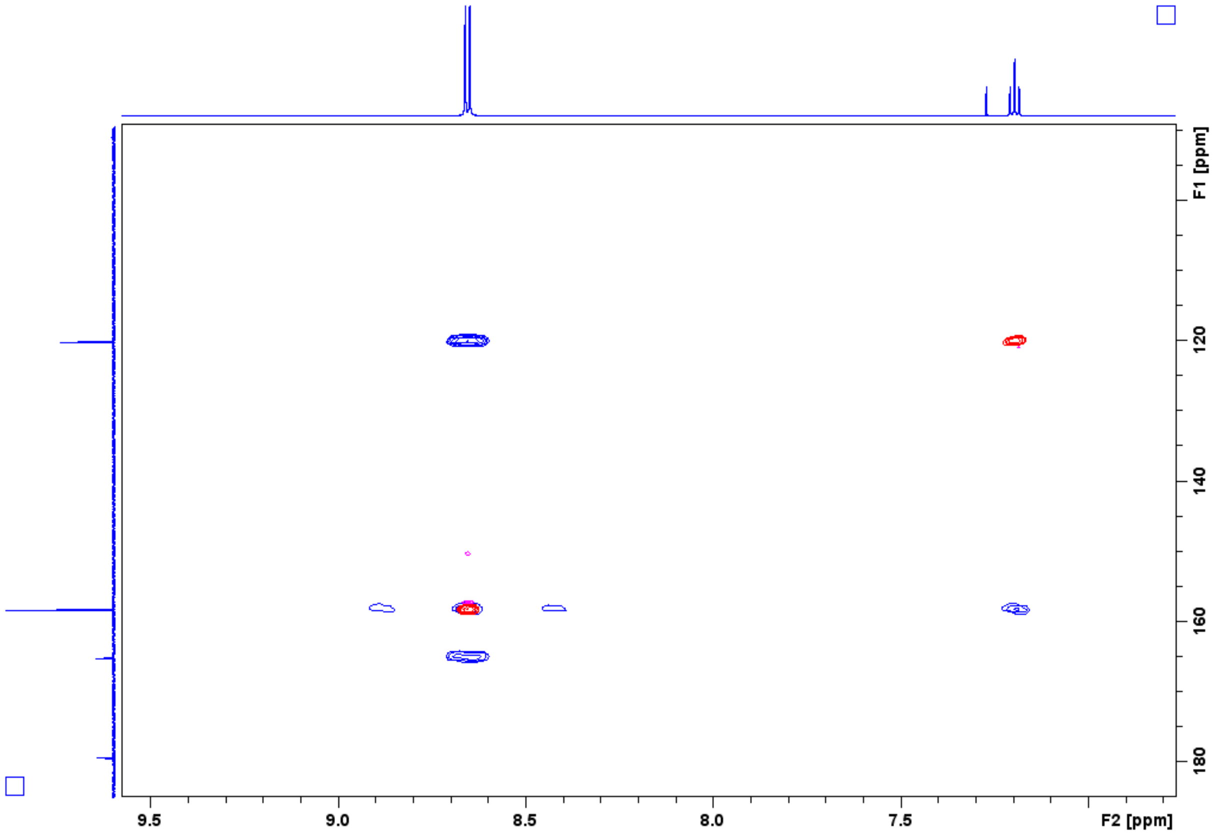

Figure S45. ^{1}H-^{13}C NMR experiments HSQC (red) and HMBC (blue) of ligand **3o** in $CDCl_3$.

<u>NMR spectra of compound **3p**</u>

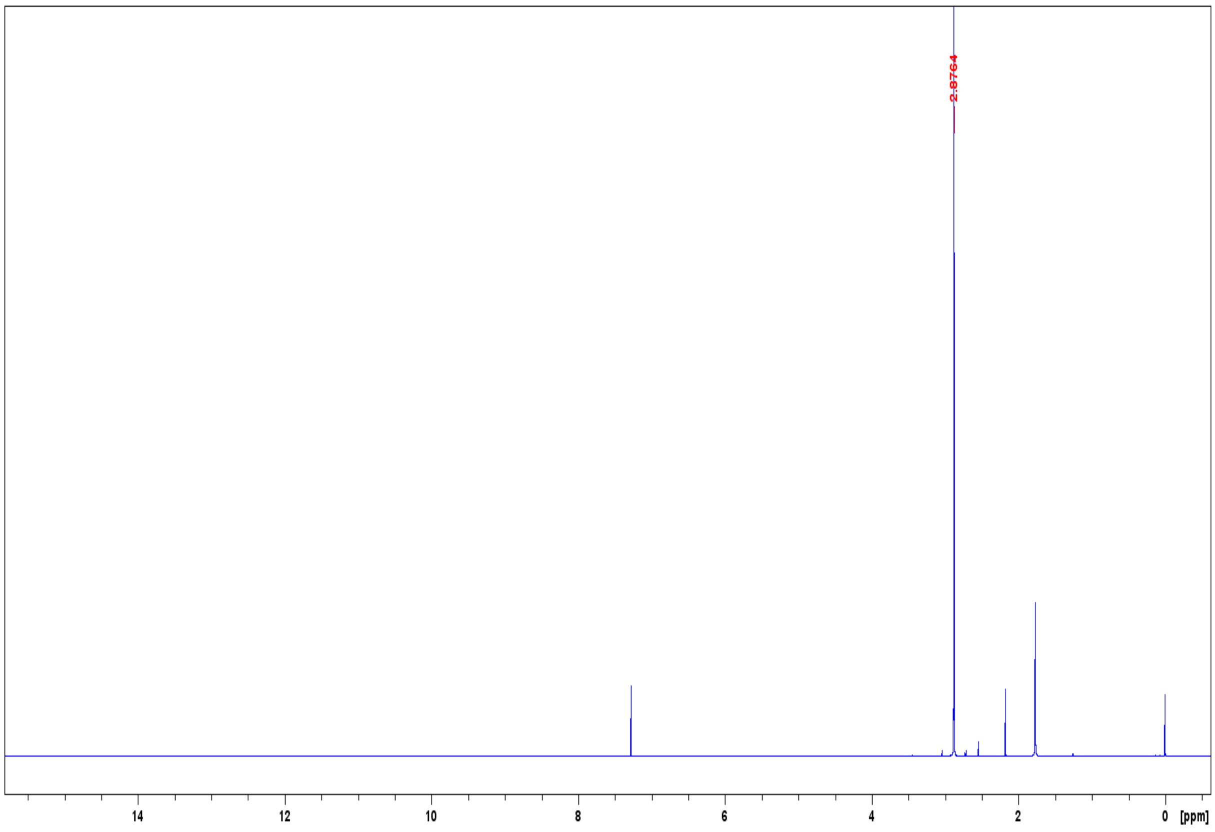

Figure S46. ^{1}H NMR spectrum of ligand **3p** in $CDCl_3$.

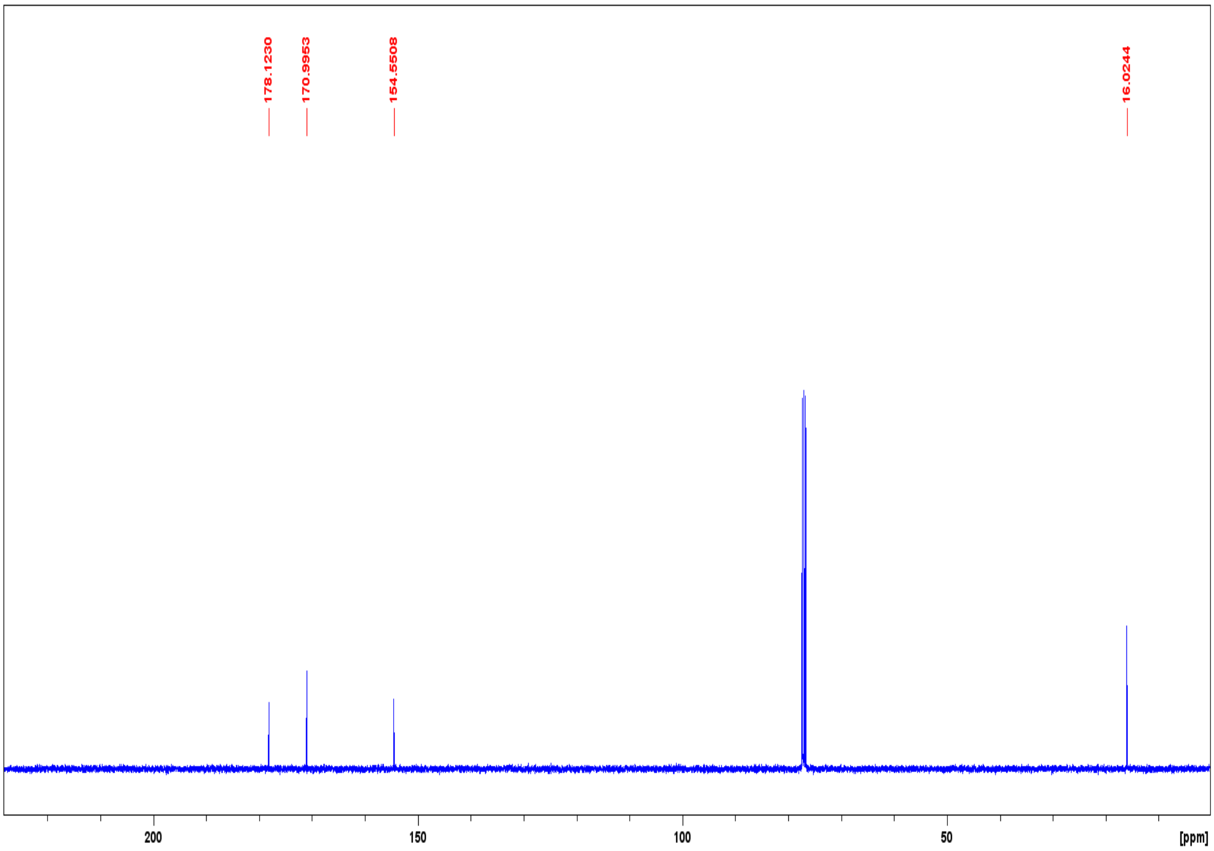

Figure S47. ^{13}C NMR spectrum of ligand **3p** in $CDCl_3$.

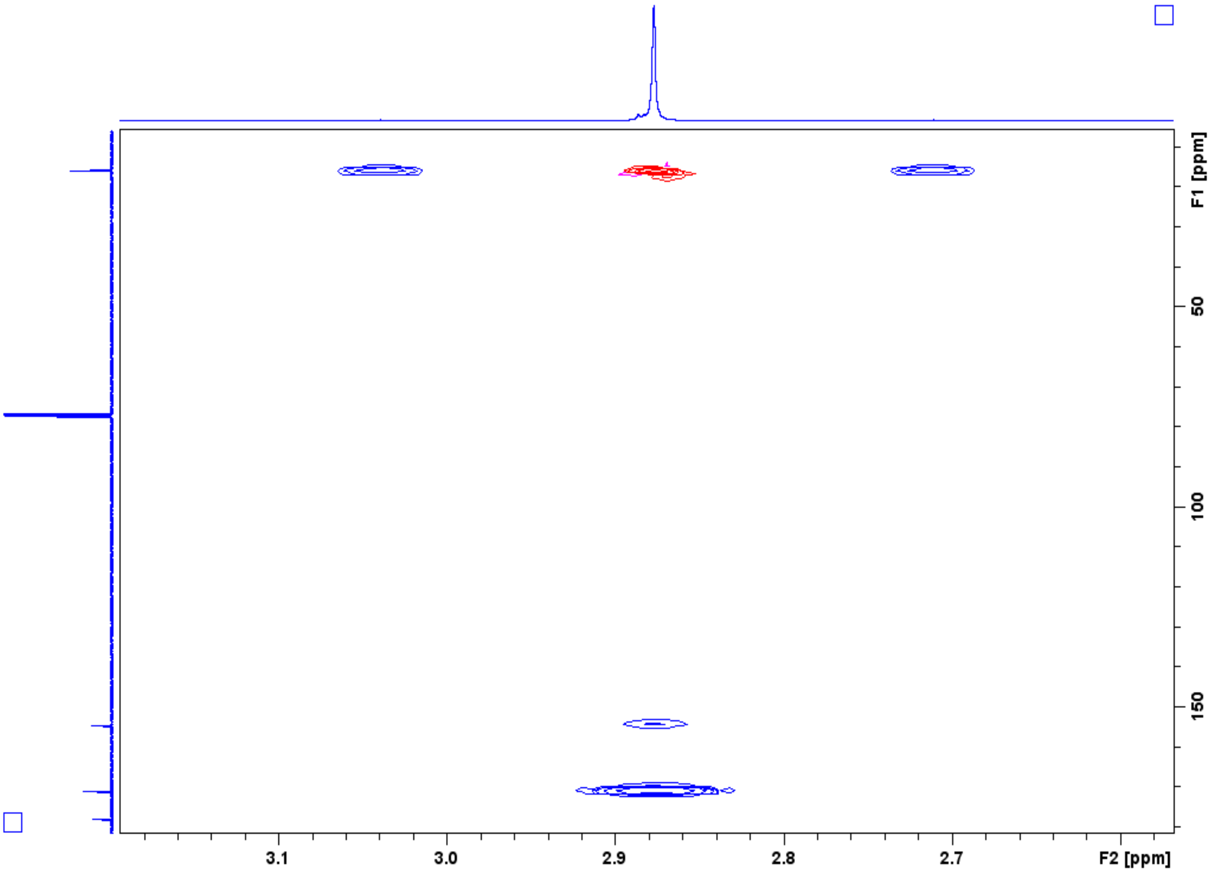

Figure S48. ^{1}H-^{13}C NMR experiments HSQC (red) and HMBC (blue) of ligand **3p** in $CDCl_3$.

NMR spectra of compound **3q**

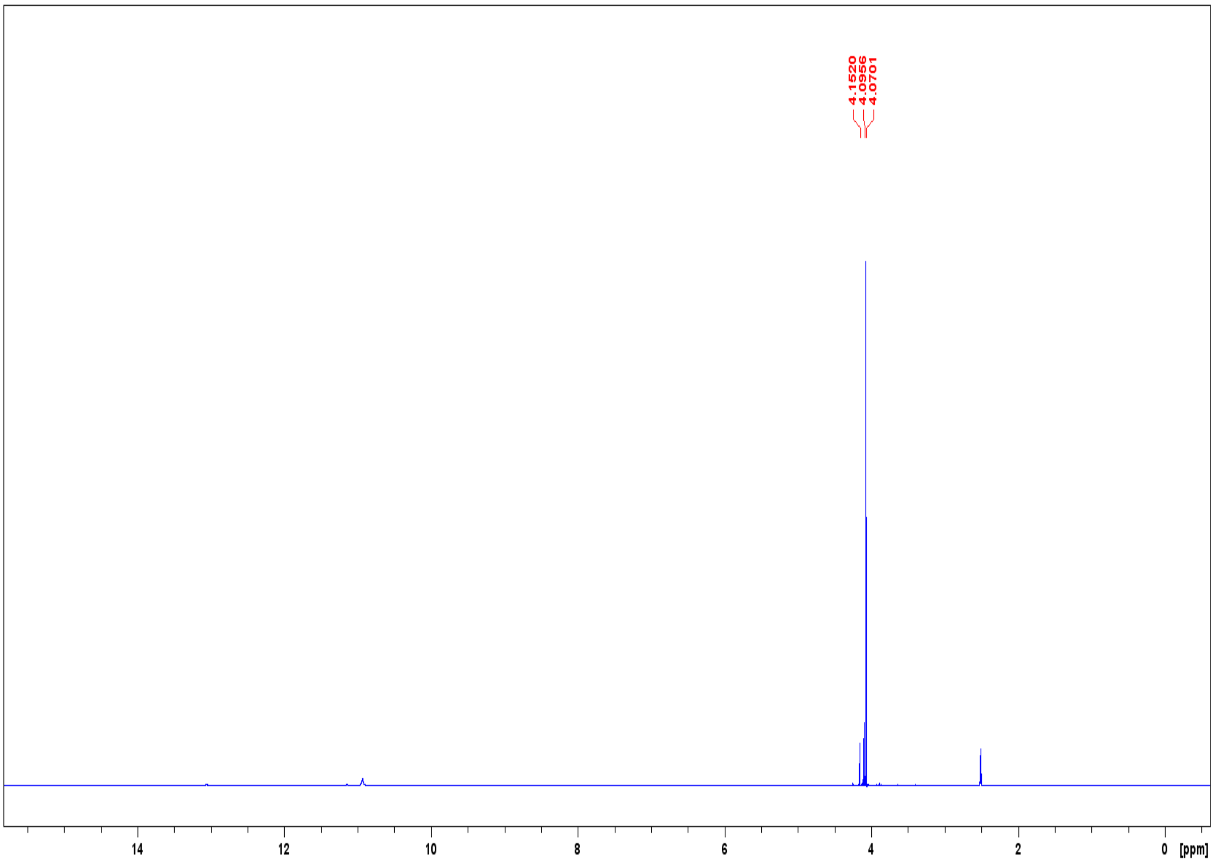

Figure S49. ^{1}H NMR spectrum of ligand **3q** in DMSO-d_6 at 353 K.

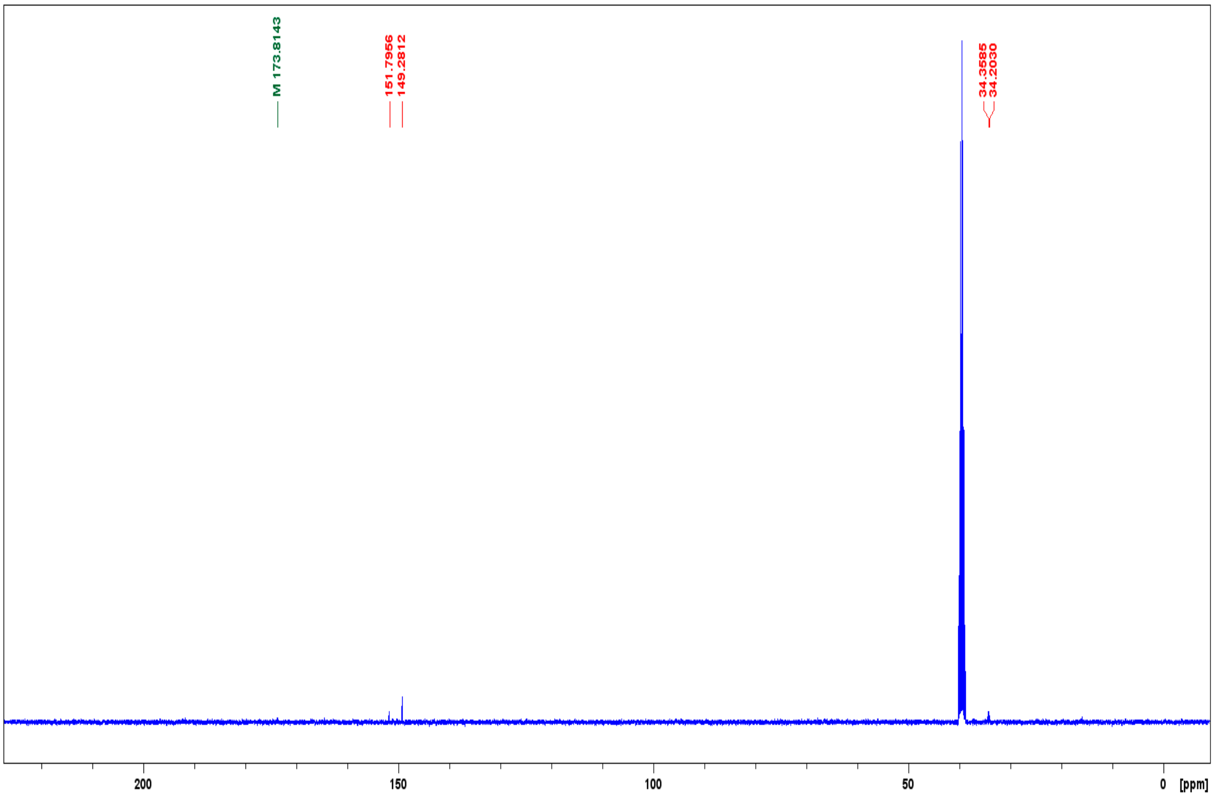

Figure S50. ^{13}C NMR spectrum of ligand **3q** in DMSO-d_6 at 353 K.

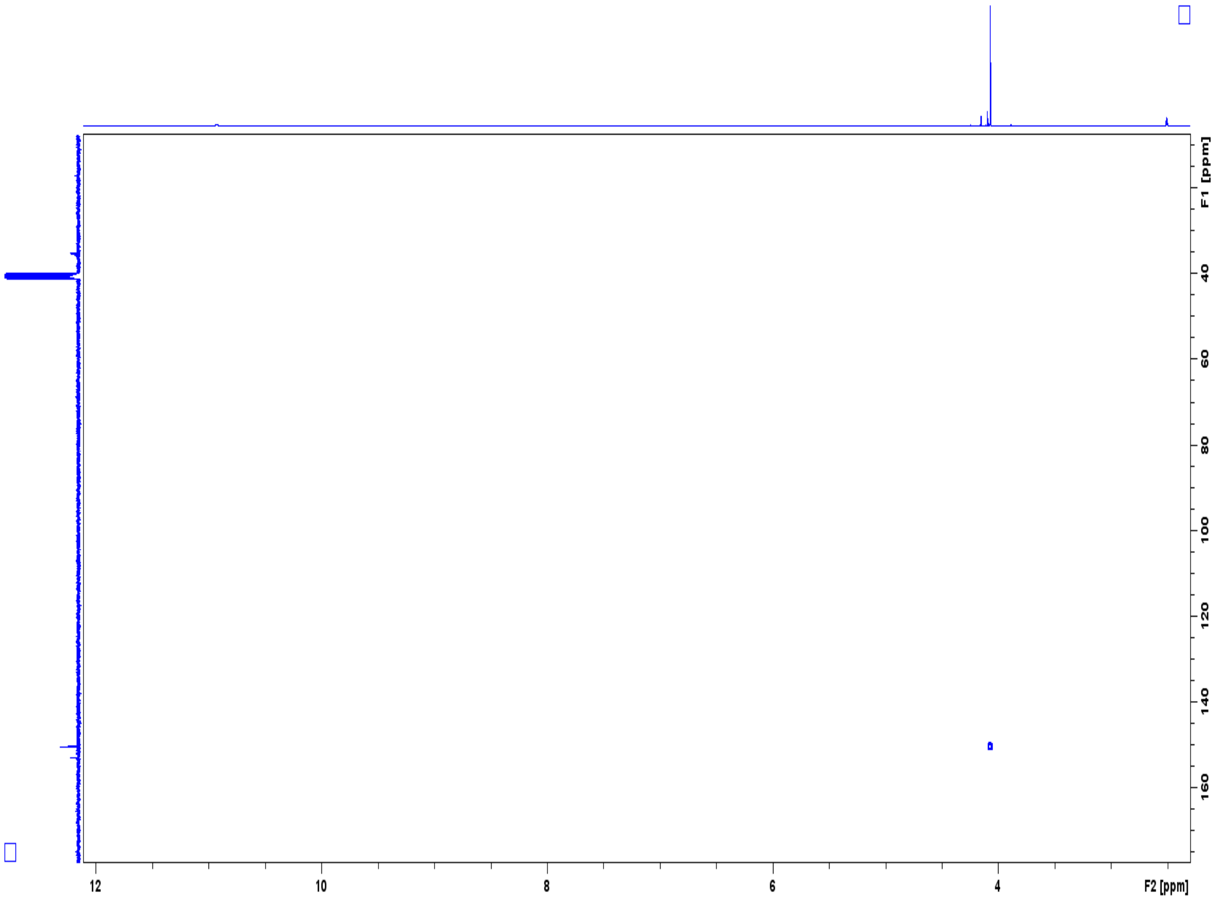

Figure S51. ^{1}H-^{13}C NMR HMBC experiment of ligand **3q** in DMSO-d_6 at 353 K.

<u>NMR spectra of compound **3r**</u>

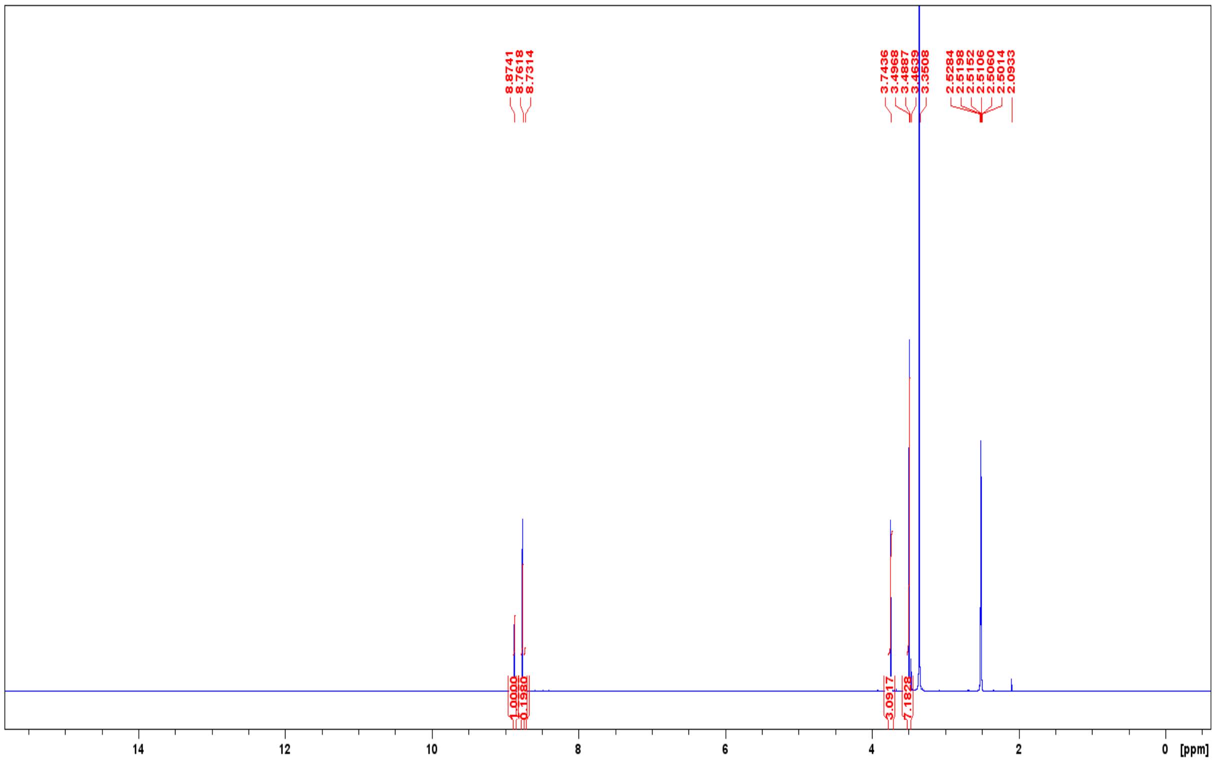

Figure S52. ^{1}H NMR spectrum of ligand **3r** in DMSO-d_6.

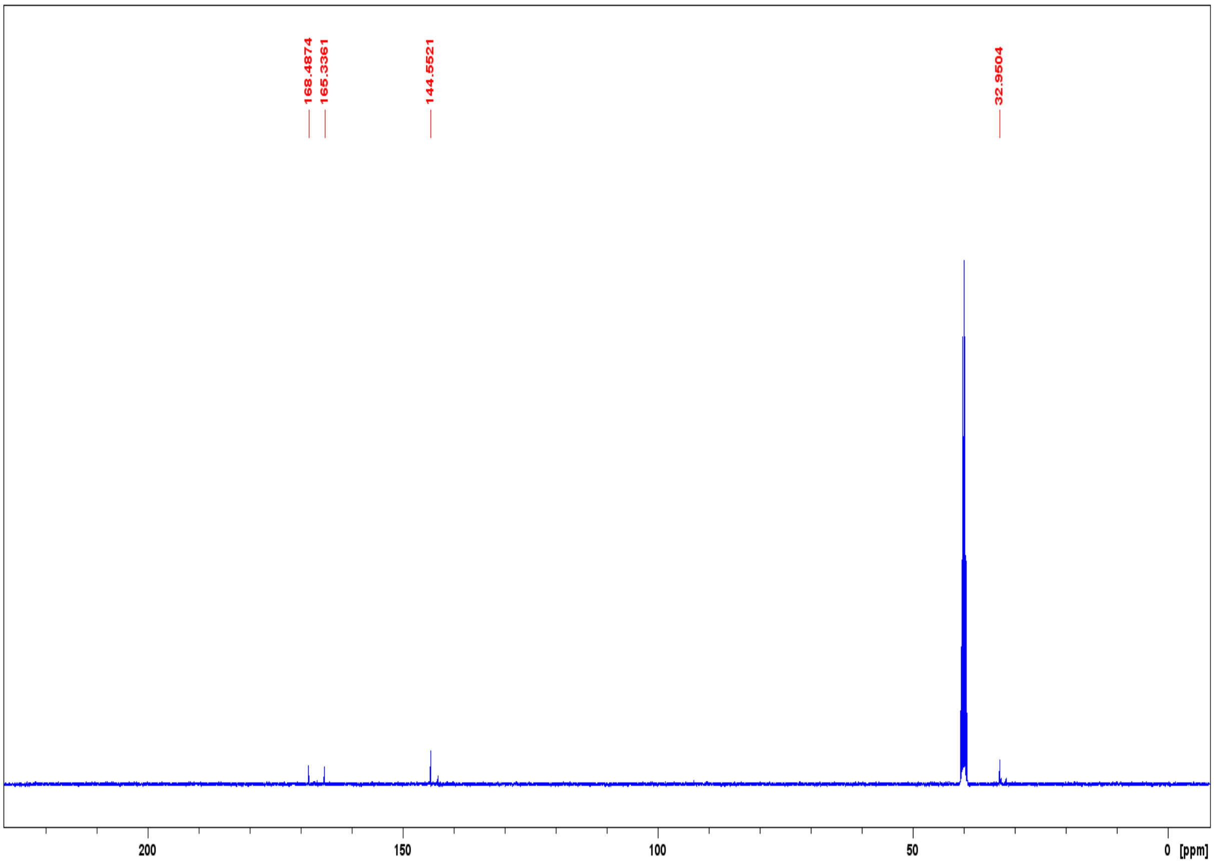

Figure S53. ^{13}C NMR spectrum of ligand **3r** in DMSO-d6.

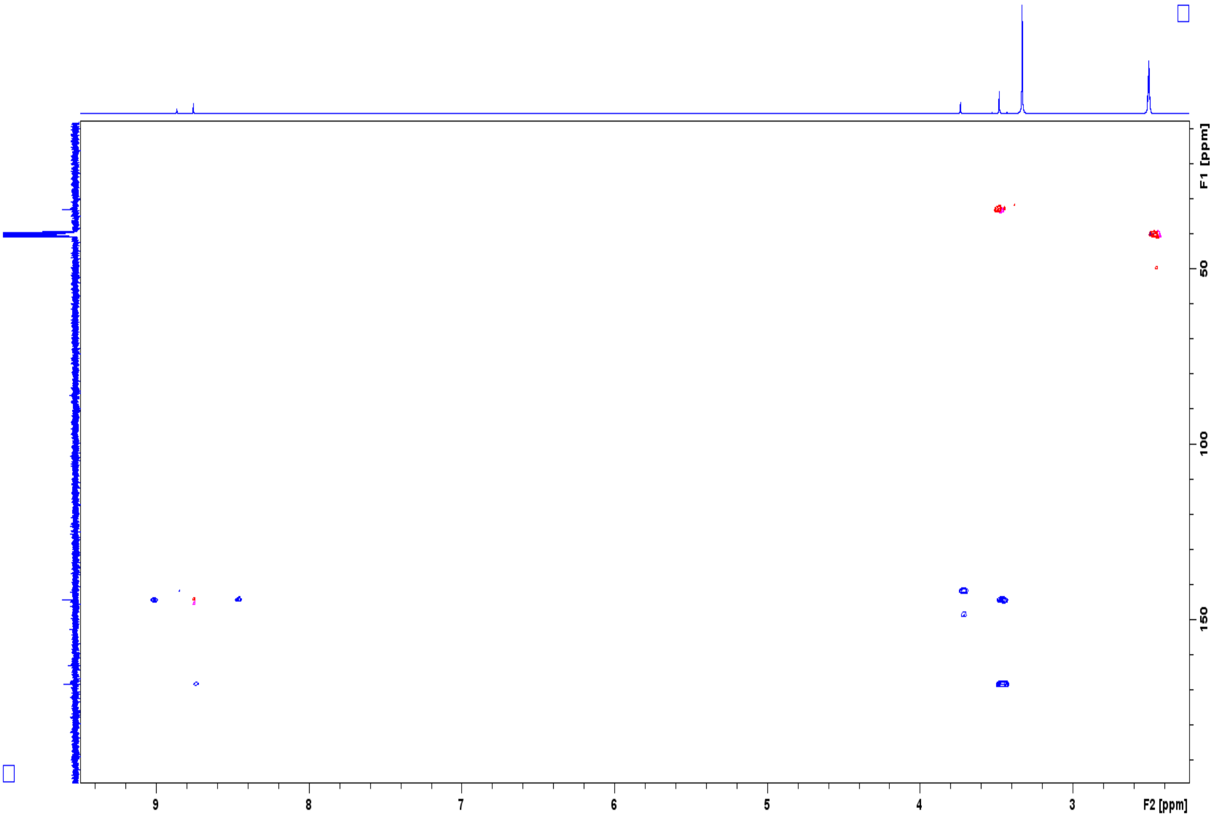

Figure S54. ^{1}H-^{13}C NMR experiments HSQC (red) and HMBC (blue) of ligand **3r** in DMSO-d6.

NMR spectra of compound **3s**

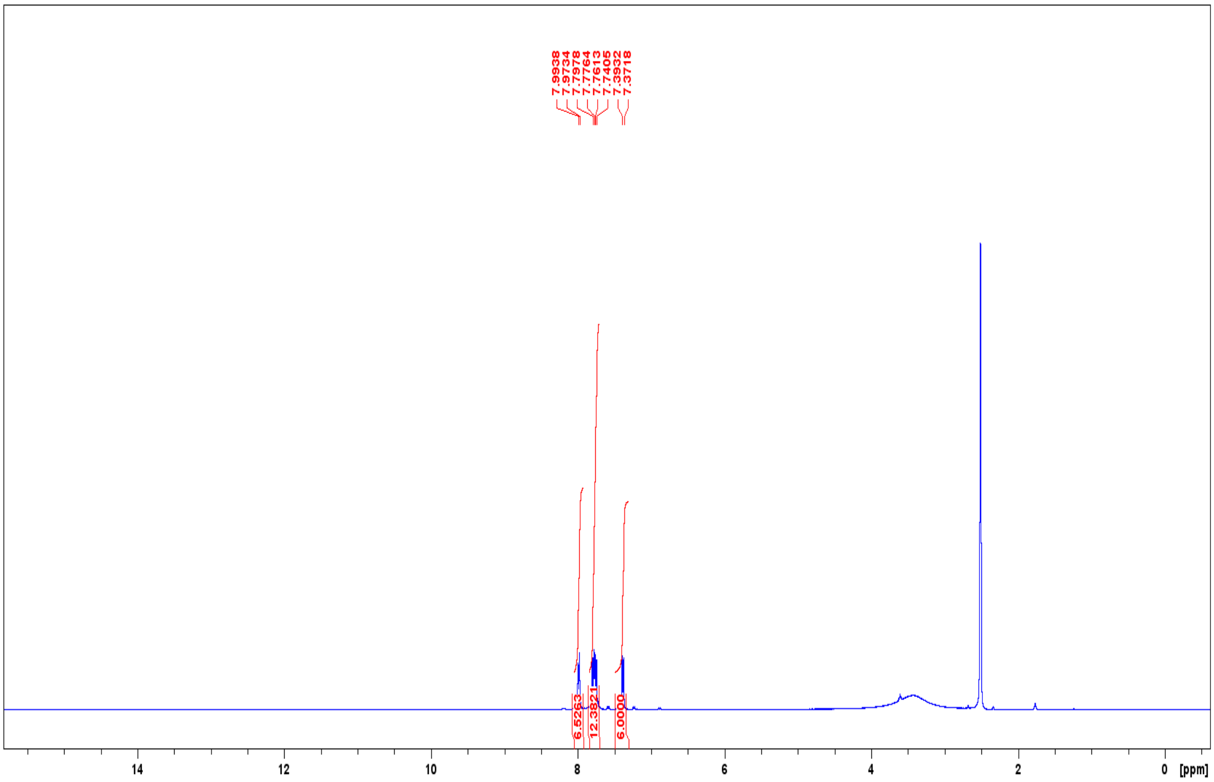

Figure S55. ^{1}H NMR spectrum of ligand **3s** in DMSO-d$_6$ at 353 K.

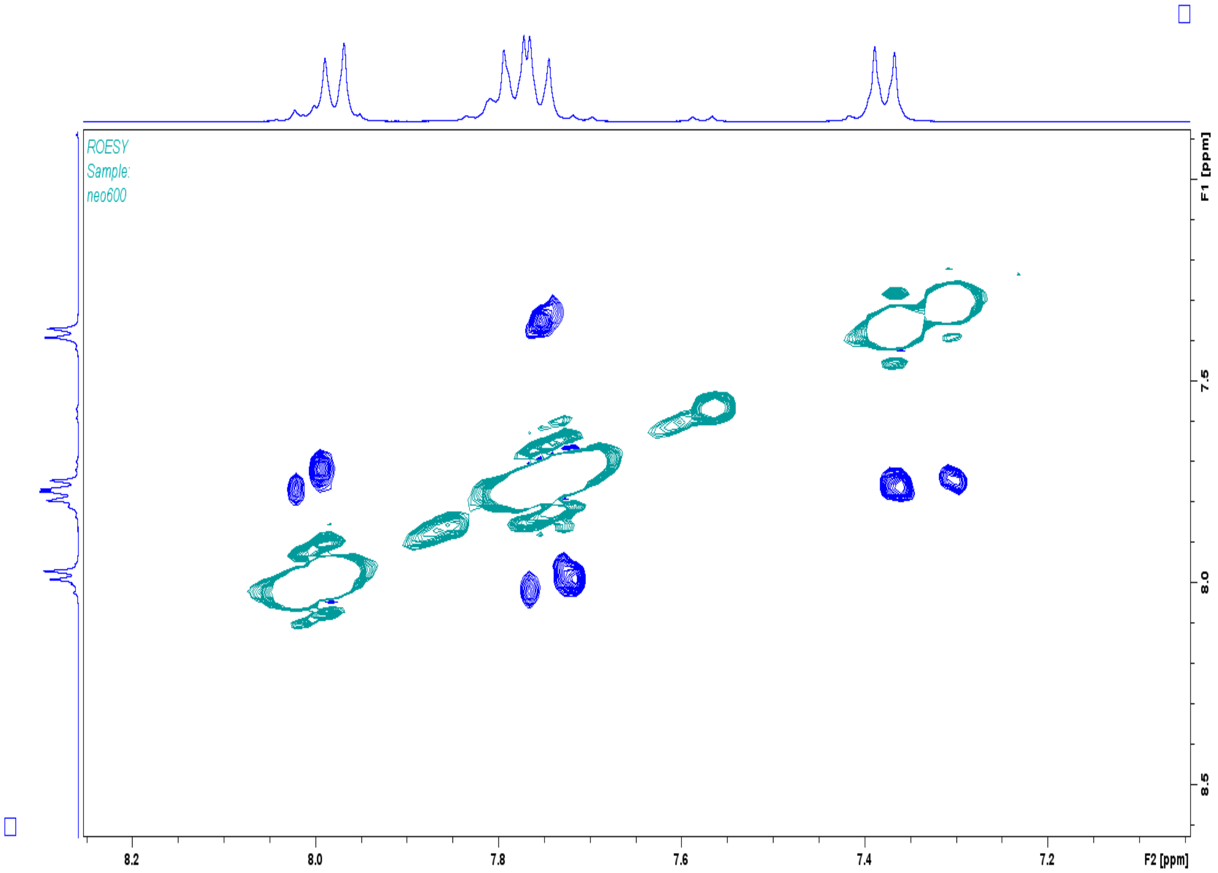

Figure S56. ^{1}H-^{1}H NMR experiment ROESY of ligand **3s** in DMSO-d$_6$ at 353 K.

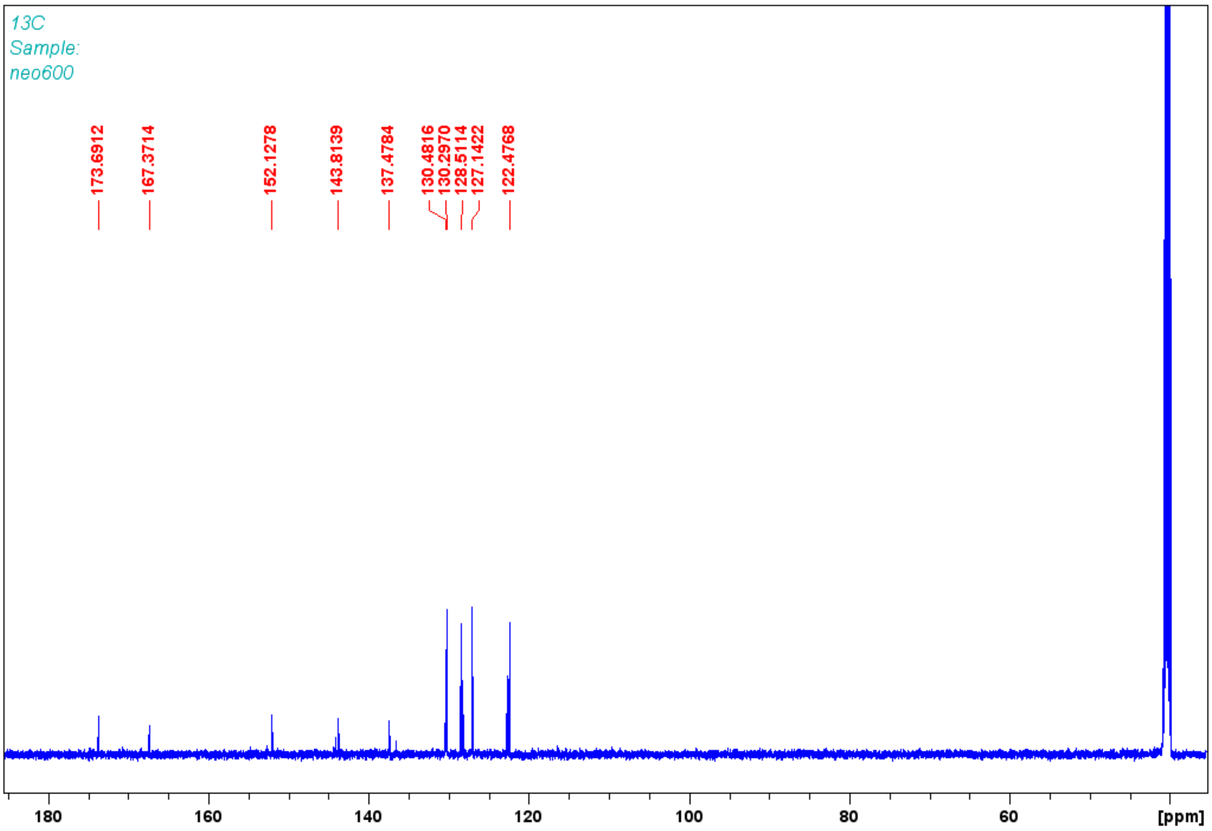

Figure S57. ^{13}C NMR spectrum of ligand **3s** in DMSO-d6 at 353 K.

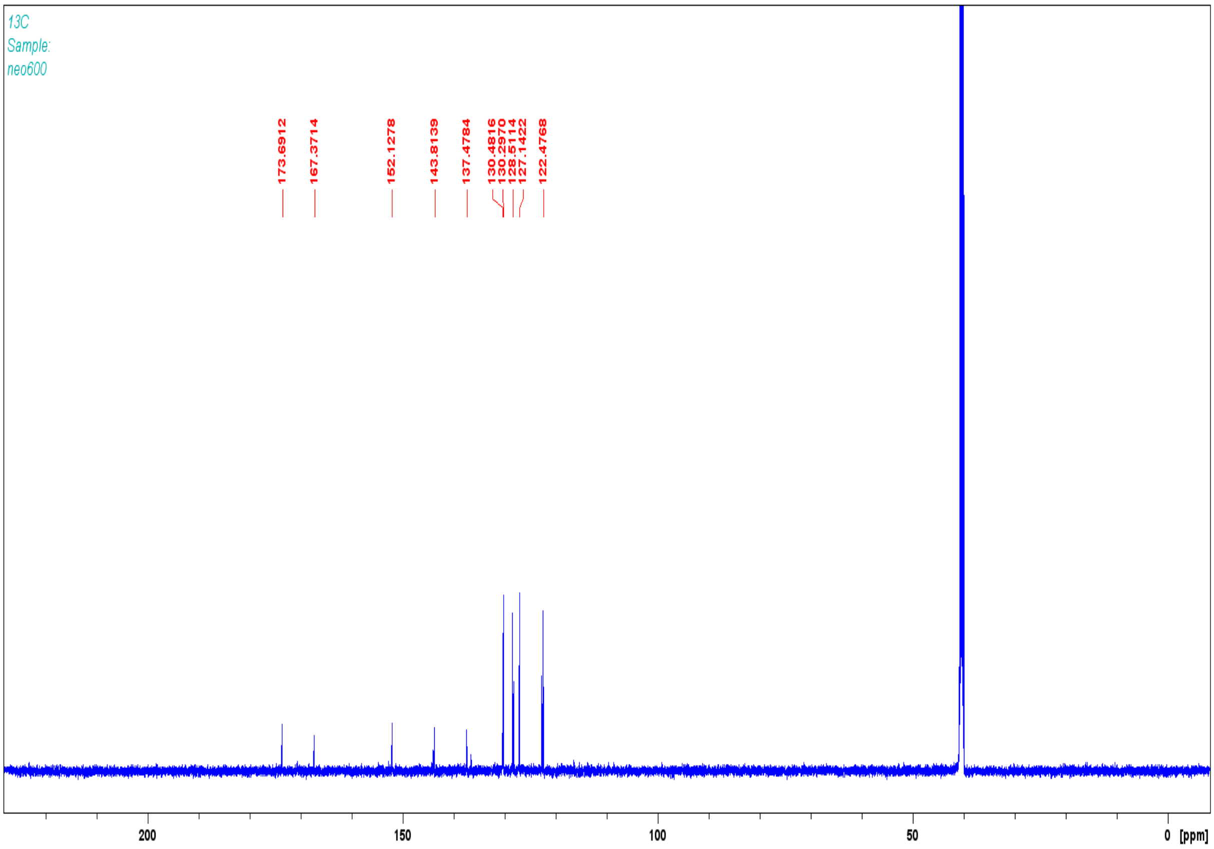

Figure S58. ^{1}H-^{13}C NMR experiments HSQC (red) and HMBC (blue) of ligand **3s** in DMSO-d6 at 353 K.

NMR spectra of compound **3t**

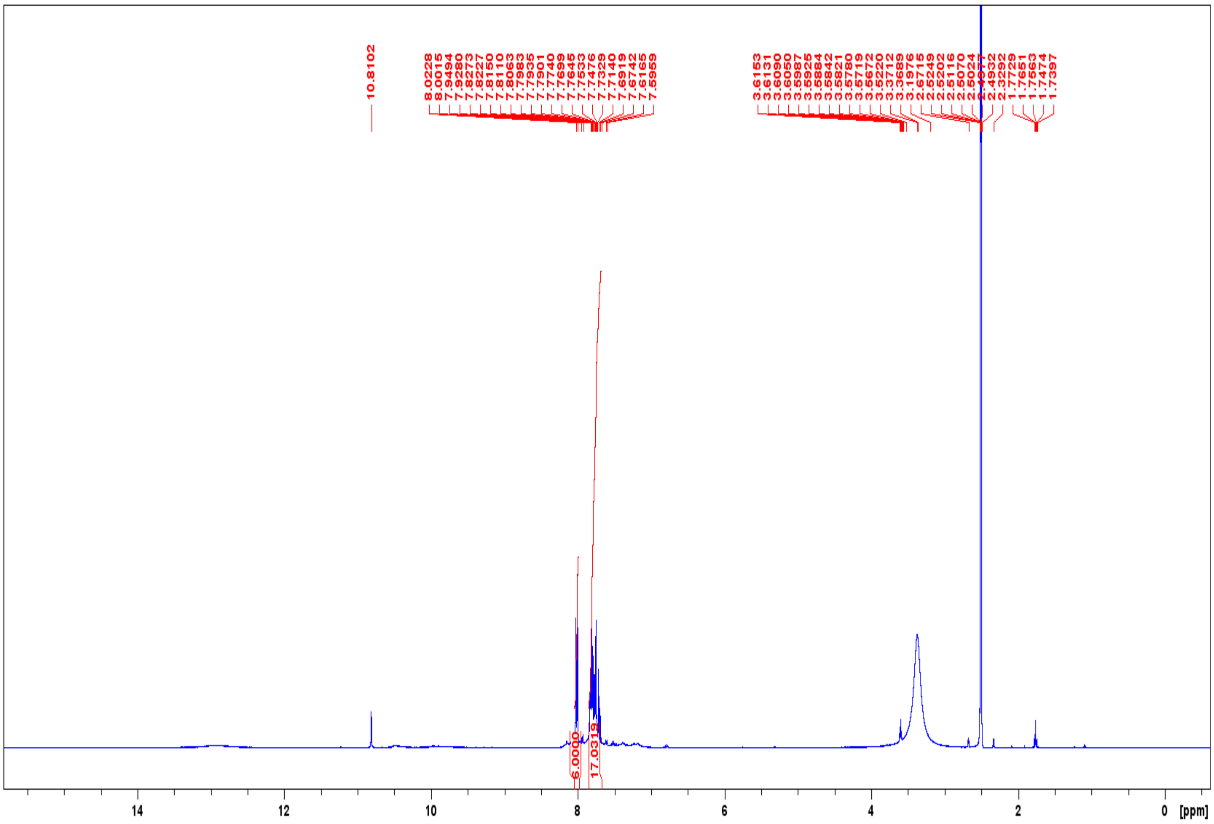

Figure S59. ^{1}H NMR spectrum of ligand **3t** in DMSO-d6 at 353 K.

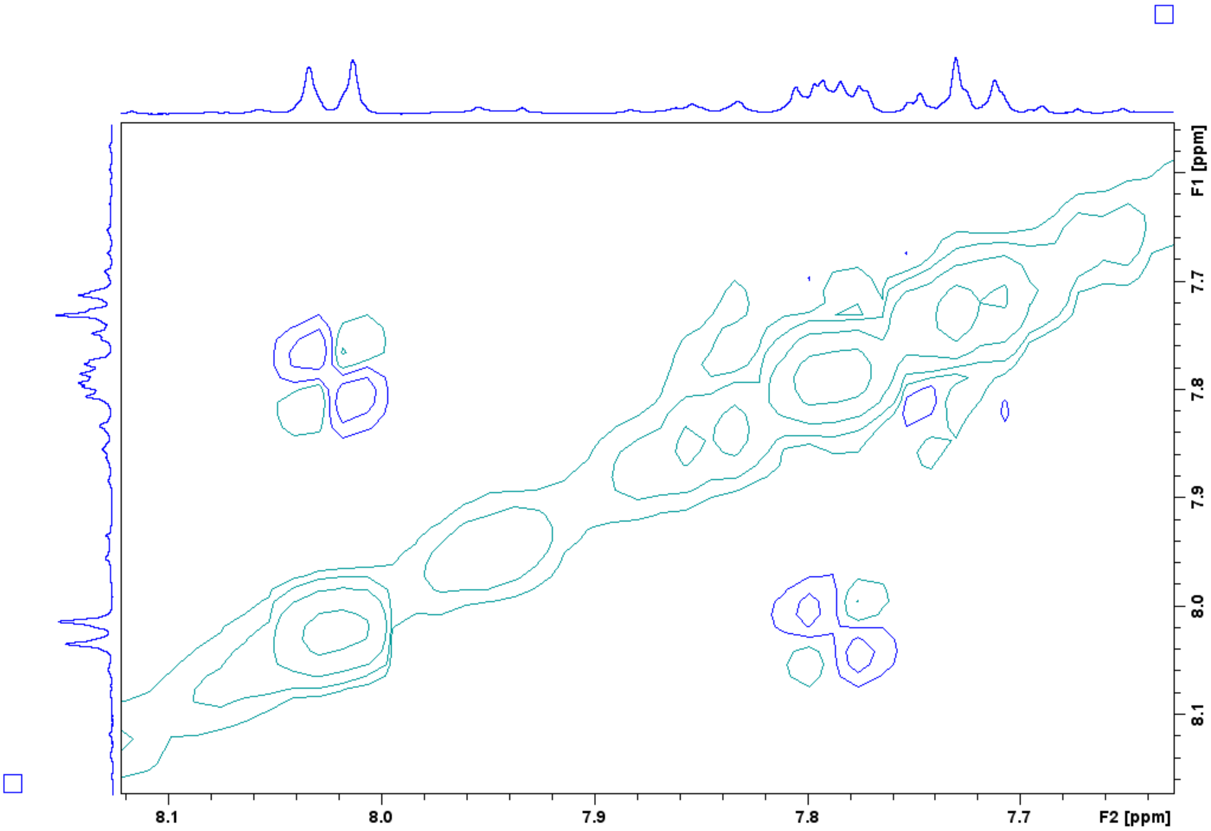

Figure S60. ^{1}H-^{1}H NMR experiment ROESY of ligand **3s** in DMSO-d6 at 353 K.

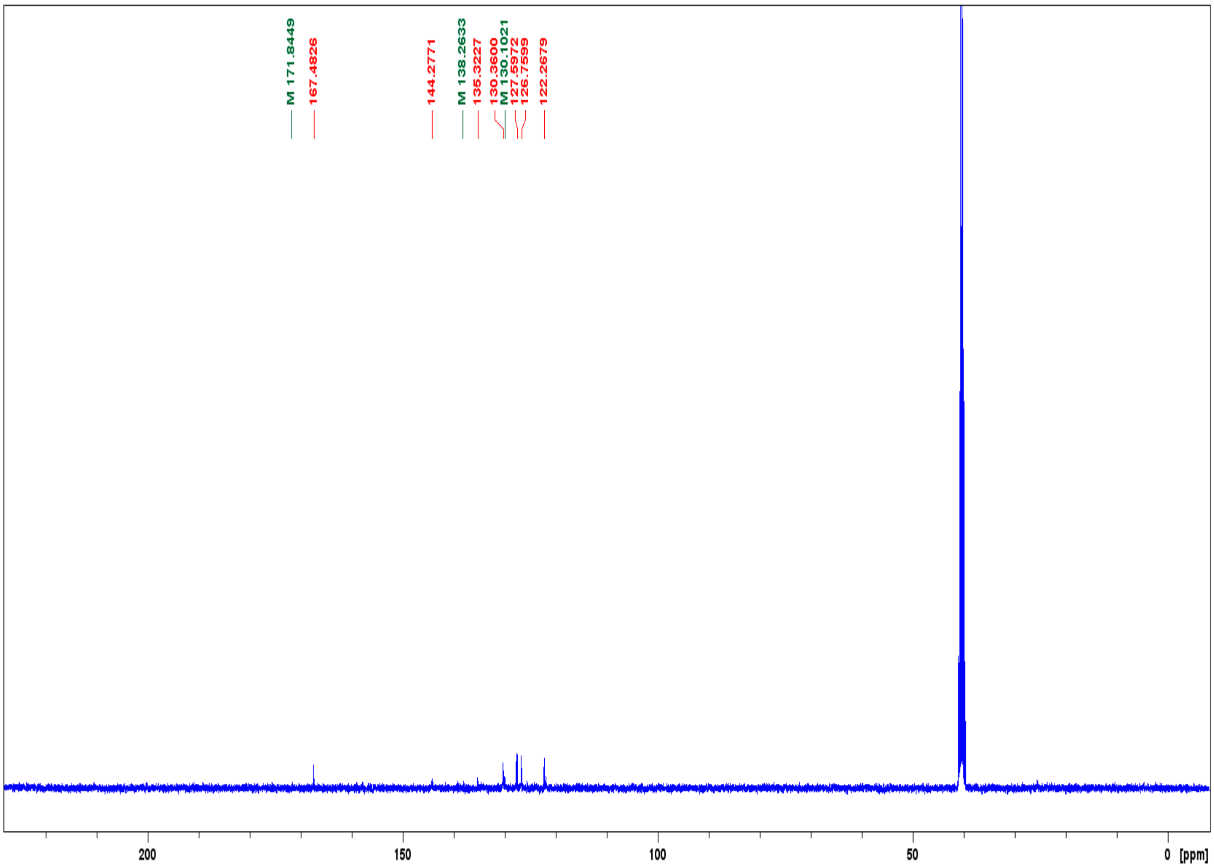

Figure S61. ^{13}C NMR spectrum of ligand **3t** in DMSO-d6 at 353 K.

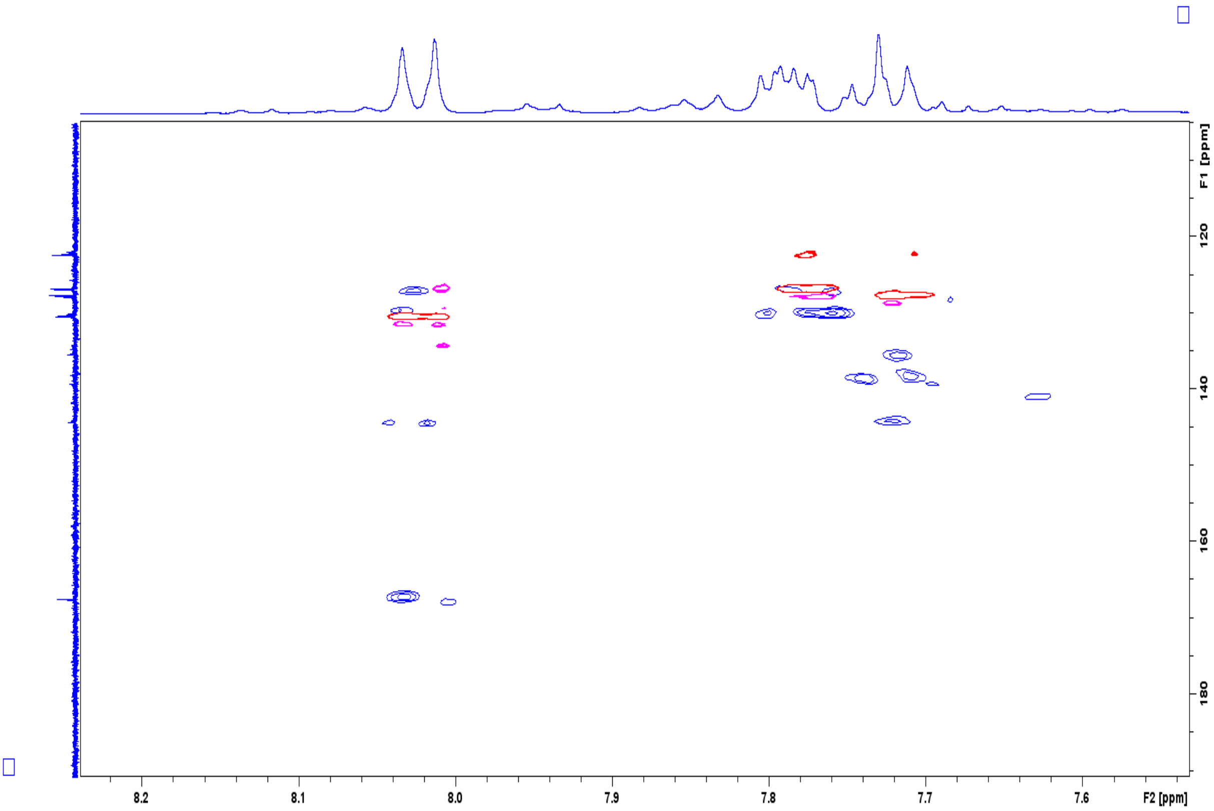

Figure S62. ^{1}H-^{13}C NMR experiments HSQC (red) and HMBC (blue) of ligand **3t** in DMSO-d6 at 353 K.

NMR spectra of ester **4**

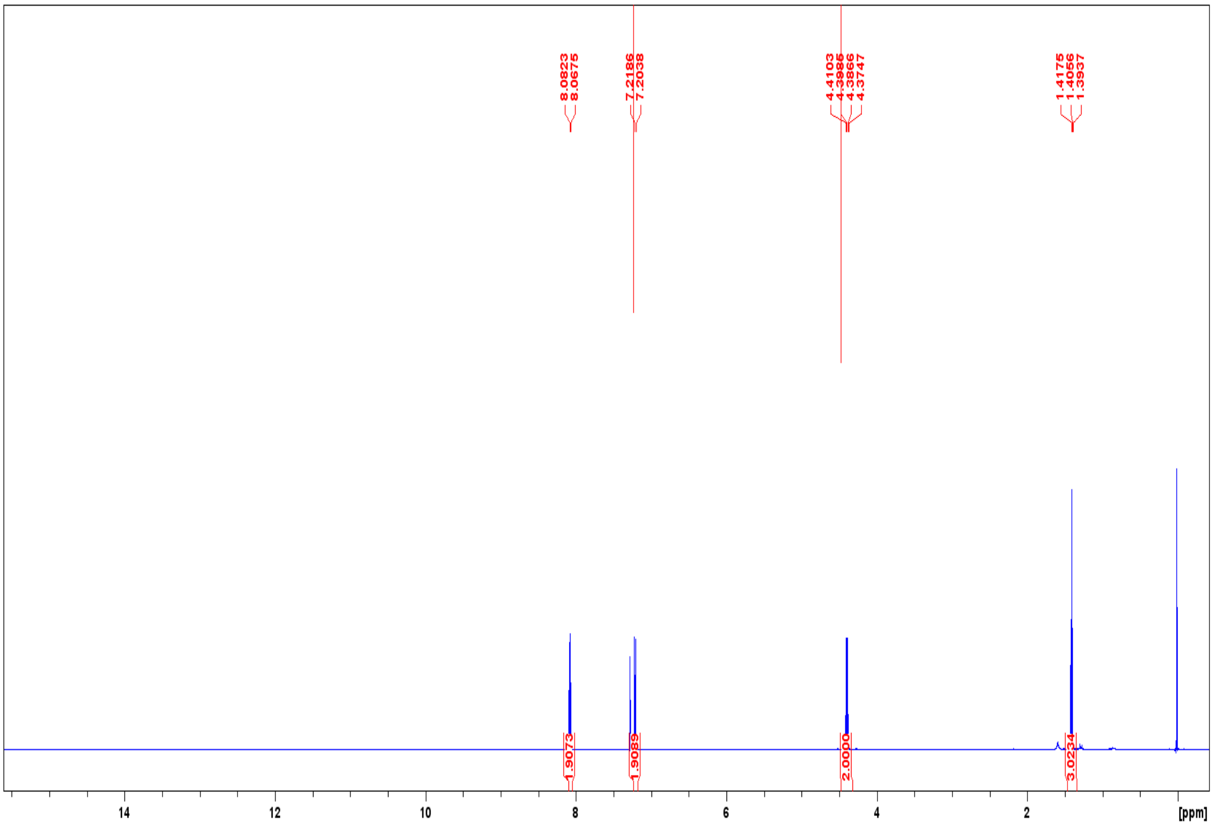

Figure S63. ^{1}H NMR spectrum of ester **4** in $CDCl_3$.

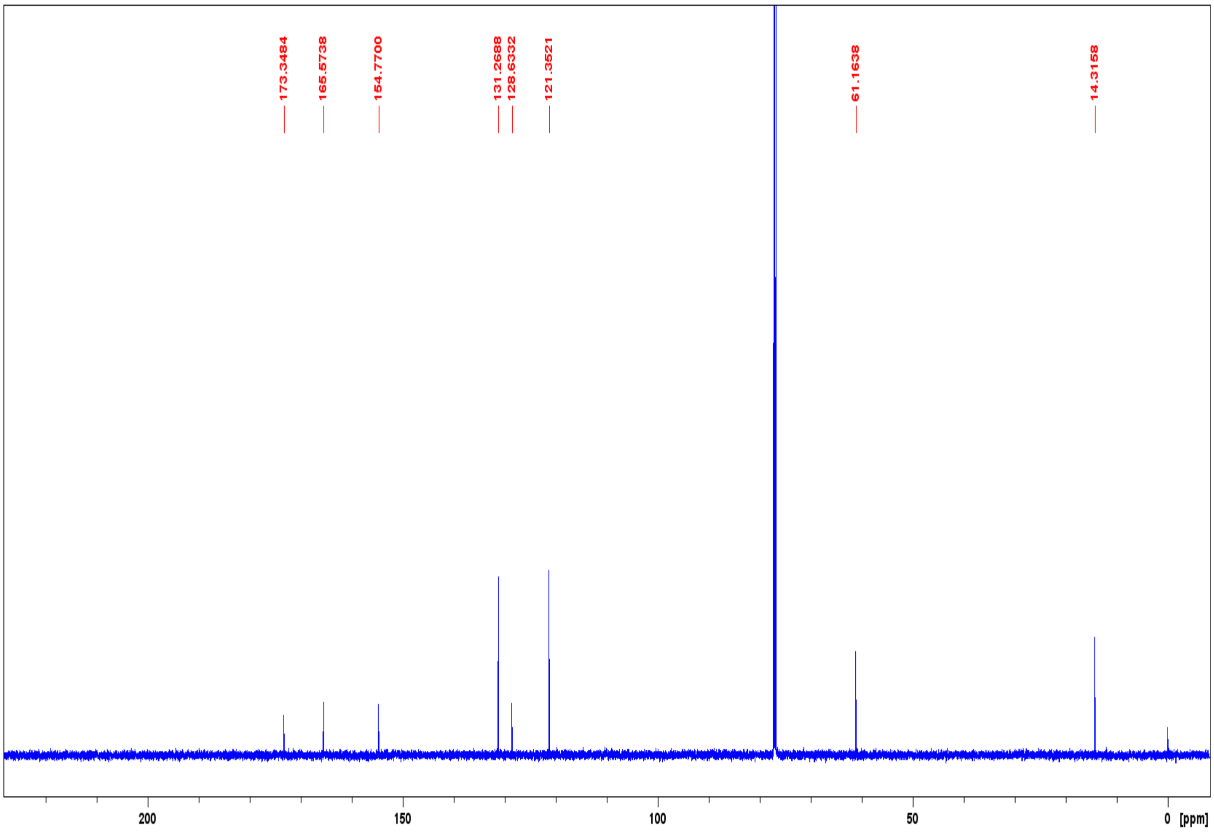

Figure S64. ^{13}C NMR spectrum of ester **4** in $CDCl_3$.

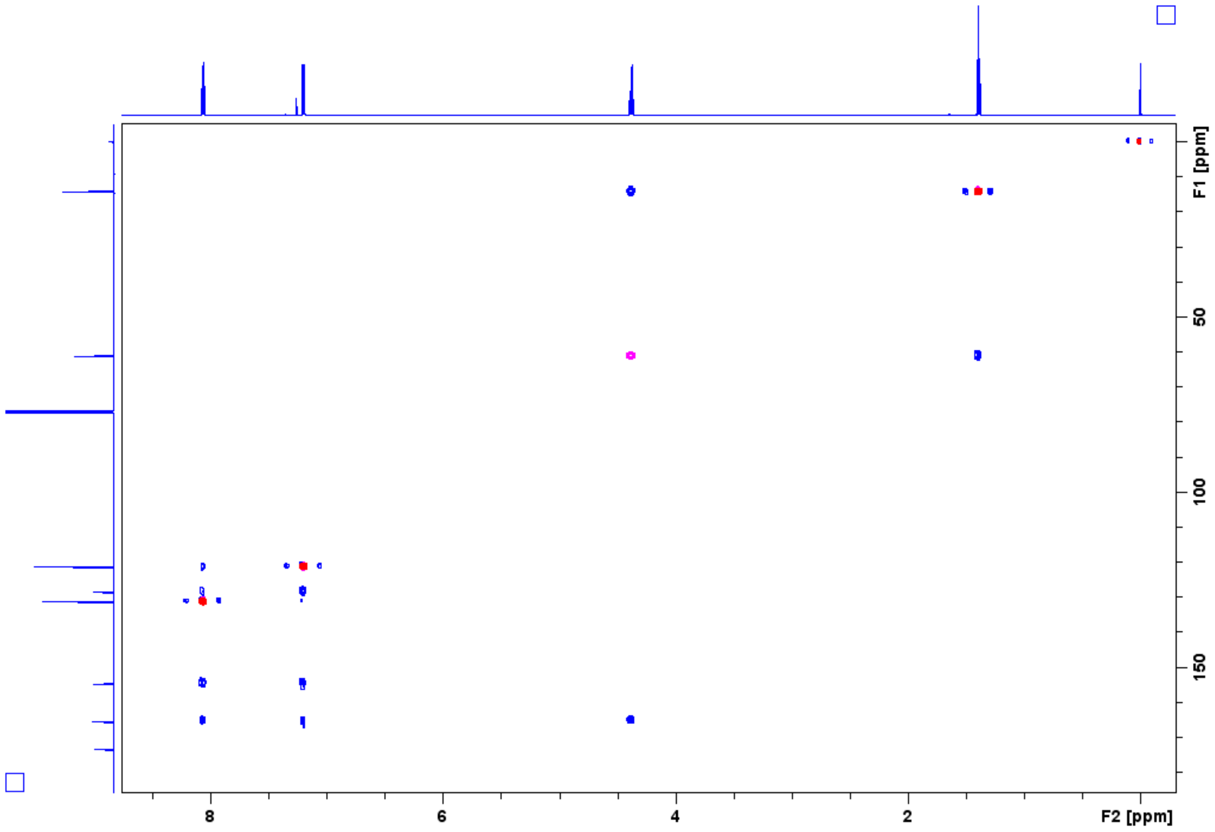

Figure S65. ^{1}H-^{13}C NMR experiments HSQC (red) and HMBC (blue) of ester **4** in $CDCl_3$.

<u>NMR spectra of 4-hydroxybenzoic acid</u>

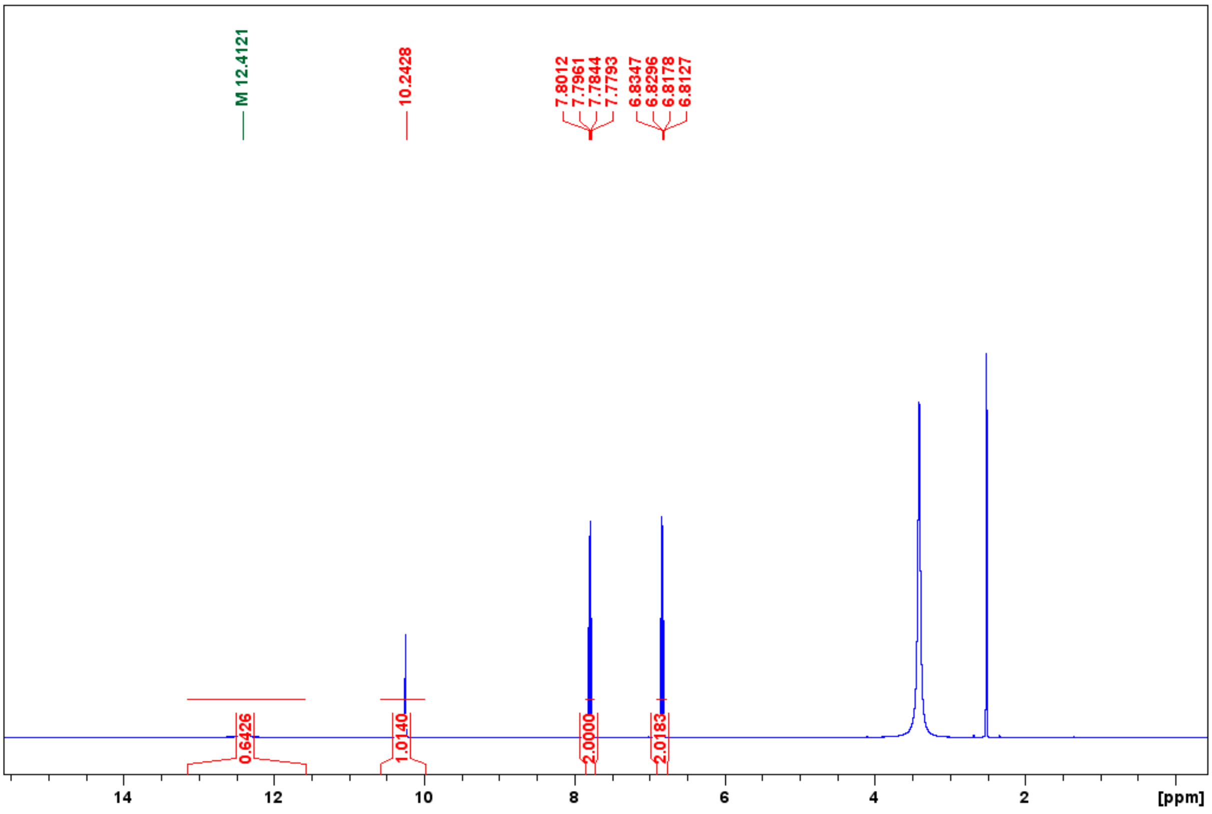

Figure S66. ^{1}H NMR spectrum of 4-hydroxybenzoic acid in DMSO-d_6.

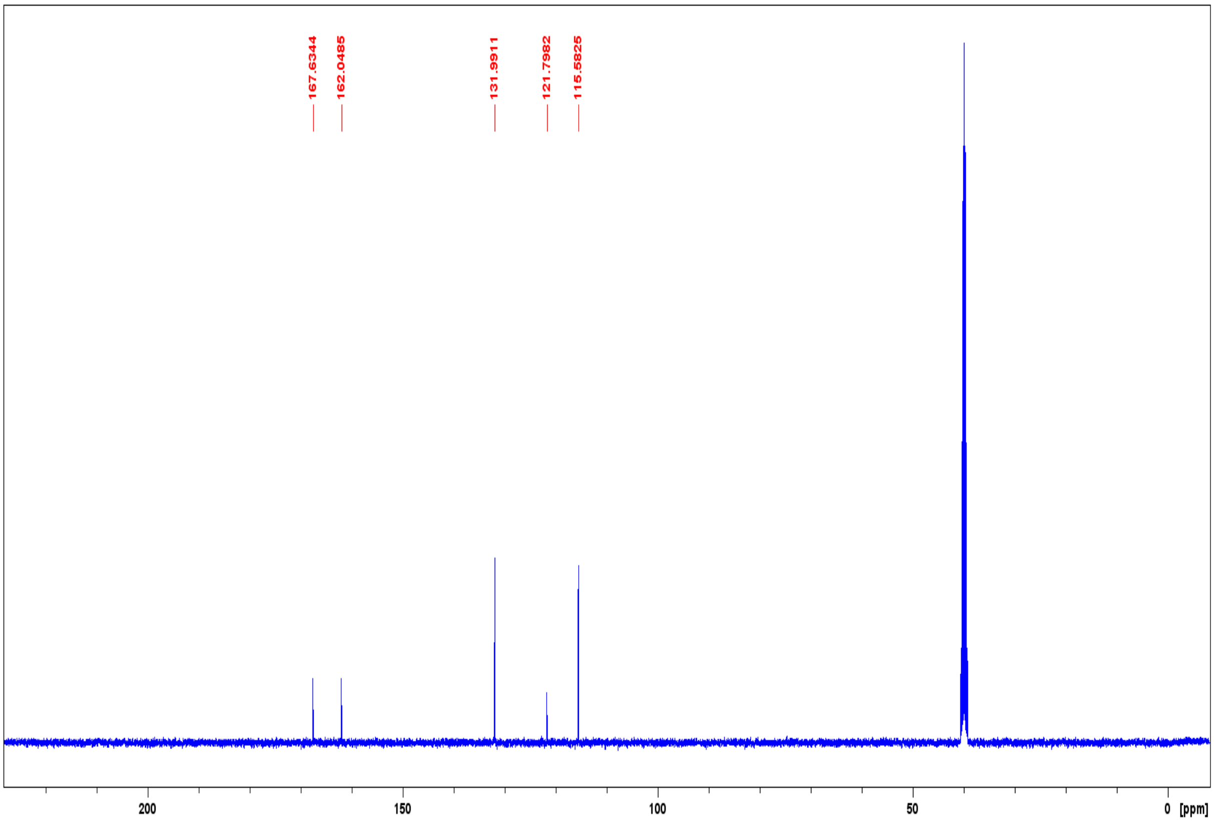

Figure S67. ^{13}C NMR spectrum of 4-hydroxybenzoic acid DMSO-d6.

<u>HRMS spectra of ligand **3a**</u>

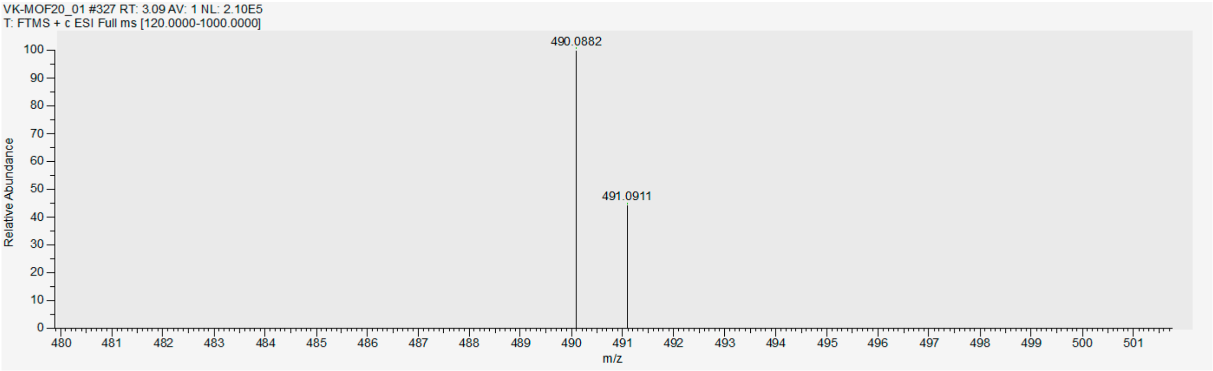

Figure S68. HRMS (HESI$^+$) spectrum of ligand **3a**.

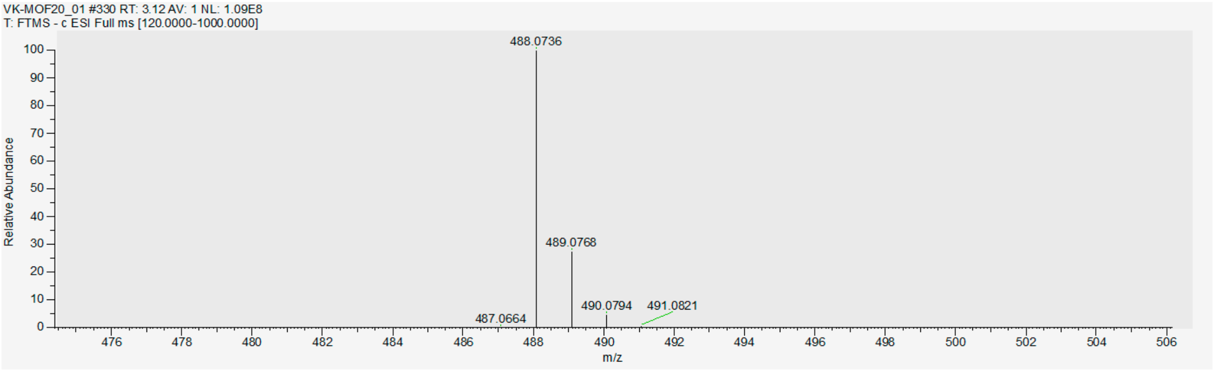

Figure S69. HRMS (HESI$^-$) spectrum of ligand **3a**.

<u>HRMS spectra of ligand **3b**</u>

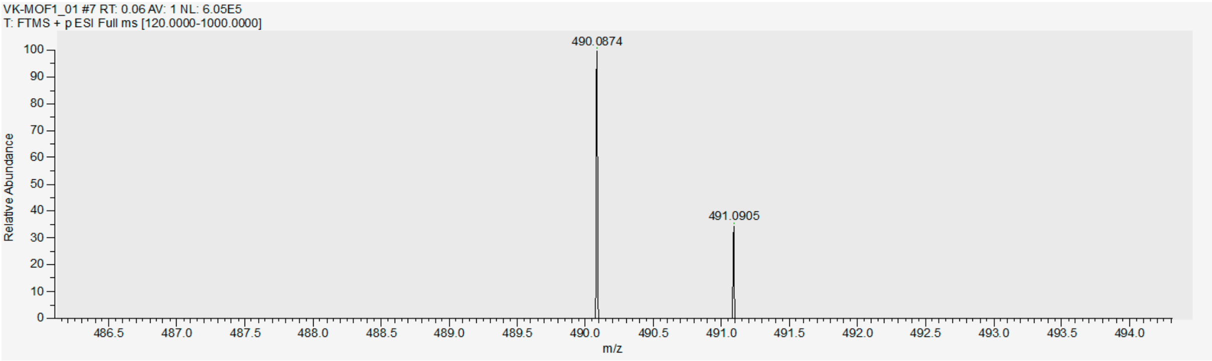

Figure S70. HRMS (HESI$^+$) spectrum of ligand **3b**.

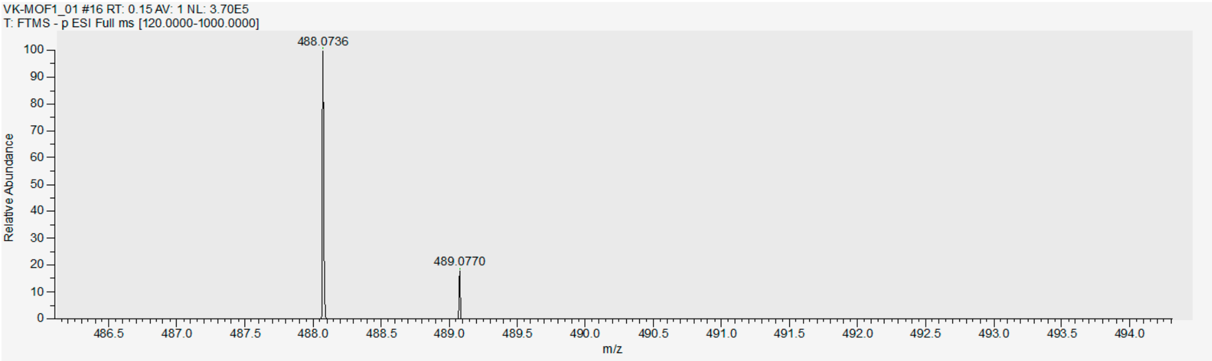

Figure S71. HRMS (HESI$^-$) spectrum of ligand **3b**.

<u>HRMS spectra of ligand **3c**</u>

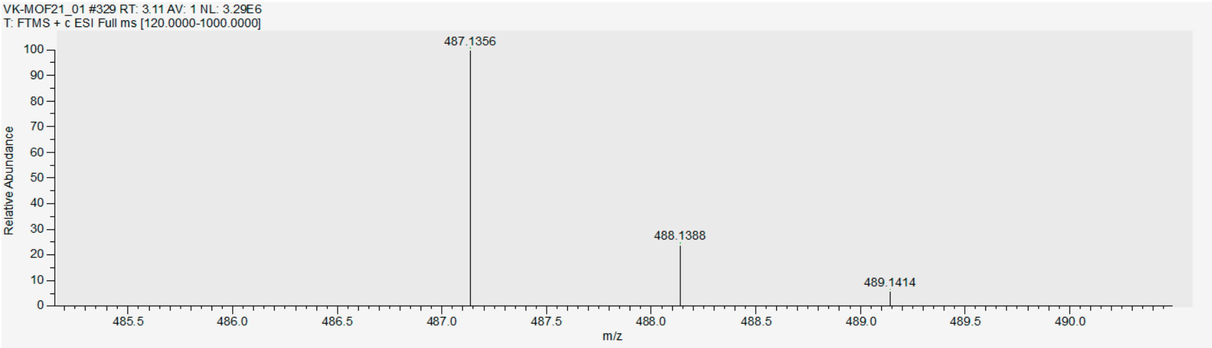

Figure S72. HRMS (HESI$^+$) spectrum of ligand **3c**.

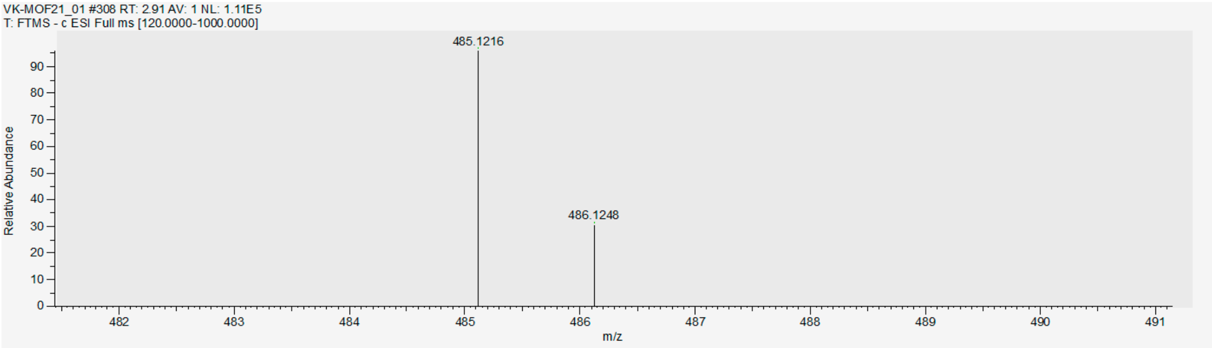

Figure S73. HRMS (HESI$^-$) spectrum of ligand **3c**.

HRMS spectra of ligand **3d**

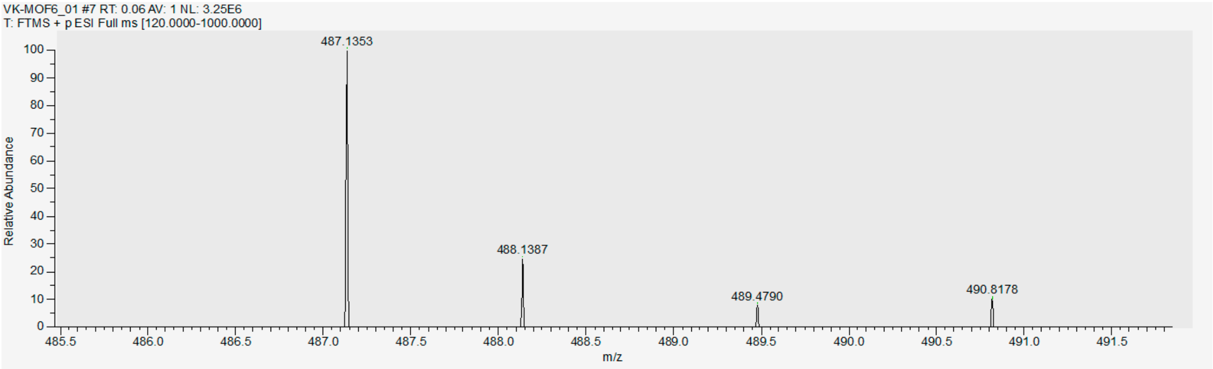

Figure S74. HRMS (HESI$^+$) spectrum of ligand **3d**.

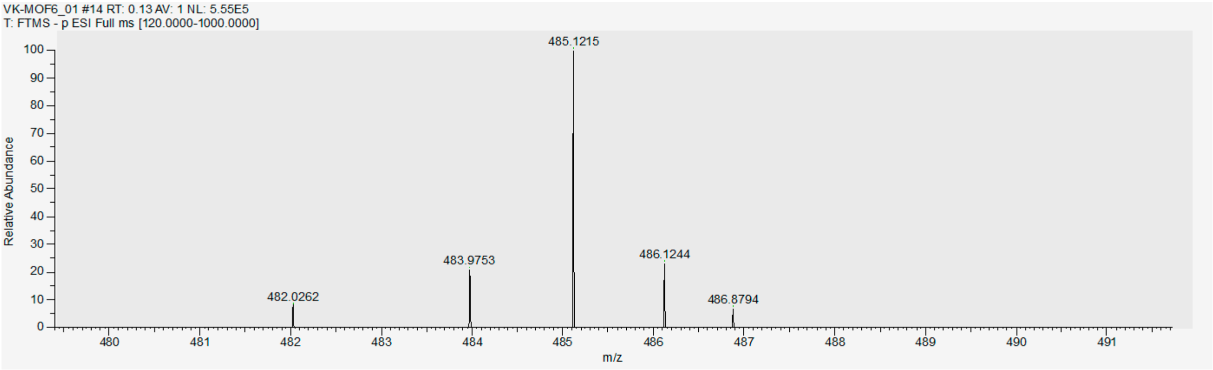

Figure S75. HRMS (HESI$^-$) spectrum of ligand **3d**.

HRMS spectra of ligand **3e**

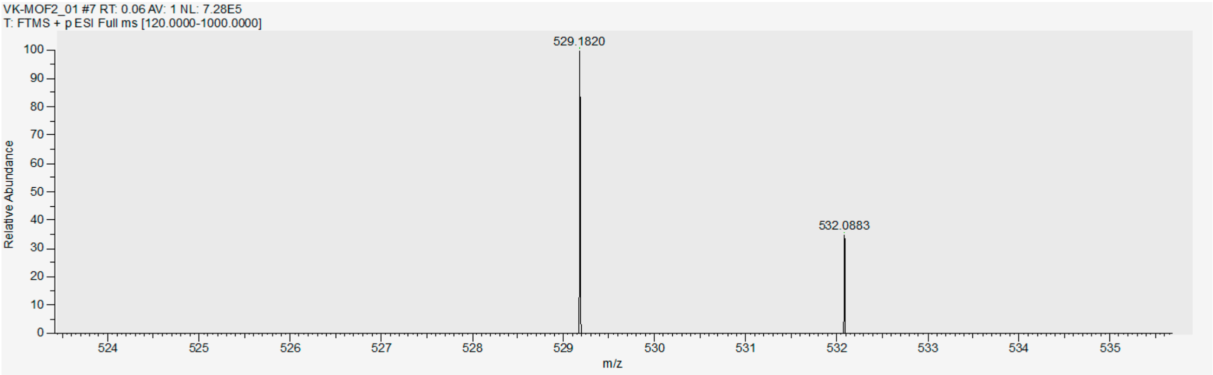

Figure S76. HRMS (HESI$^+$) spectrum of ligand **3e**.

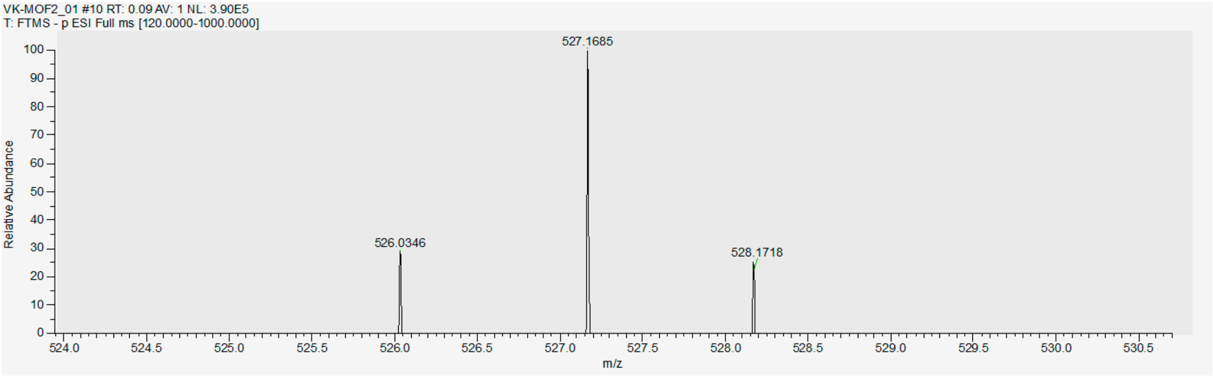

Figure S77. HRMS (HESI$^-$) spectrum of ligand **3e**.

HRMS spectra of ligand **3f**

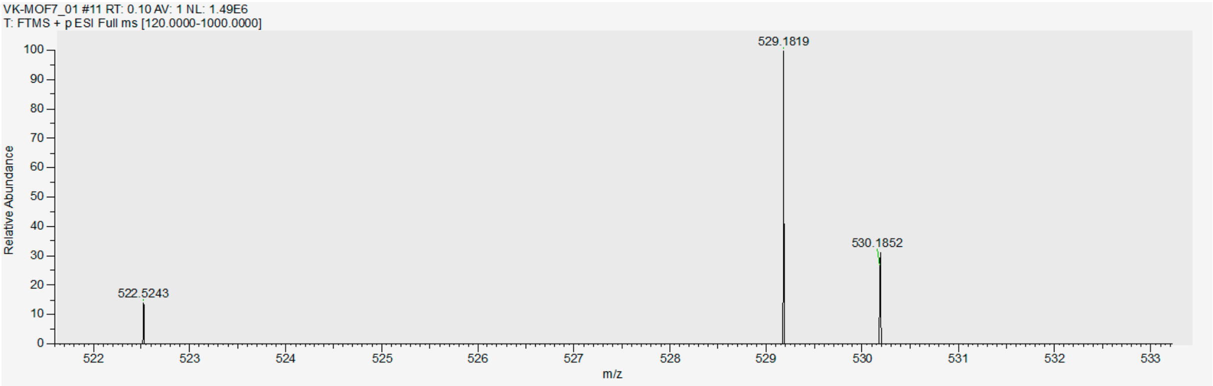

Figure S78. HRMS (HESI$^+$) spectrum of ligand **3f**.

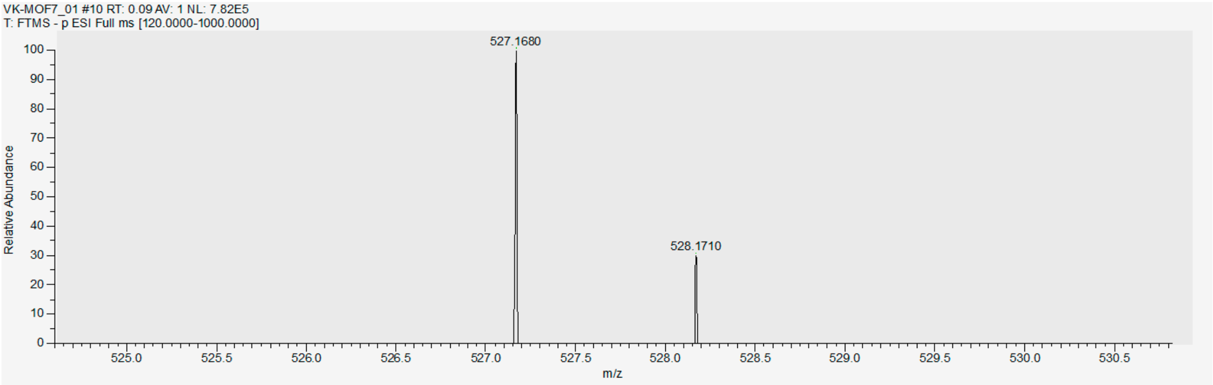

Figure S79. HRMS (HESI$^-$) spectrum of ligand **3f**.

HRMS spectra of ligand **3g**

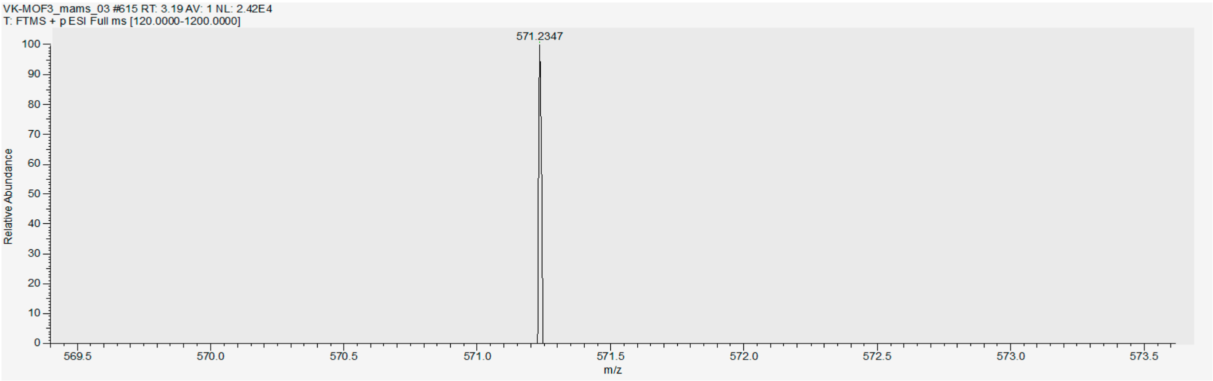

Figure S80. HRMS (HESI$^+$) spectrum of ligand **3g**.

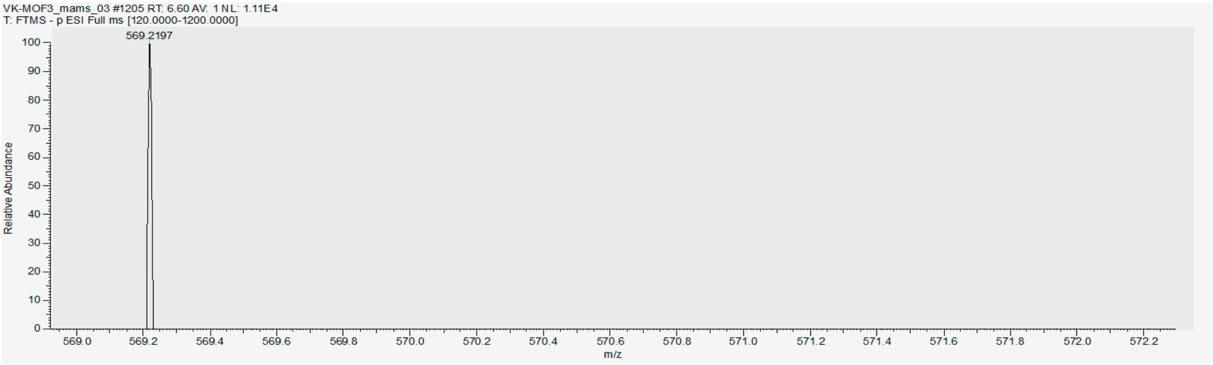

Figure S81. HRMS (HESI$^-$) spectrum of ligand **3g**.

HRMS spectra of ligand **3h**

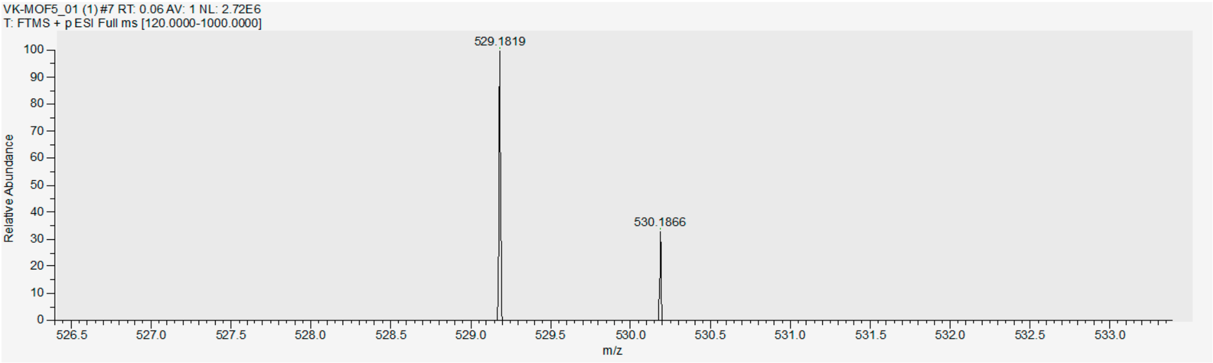

Figure S82. HRMS (HESI$^+$) spectrum of ligand **3h**.

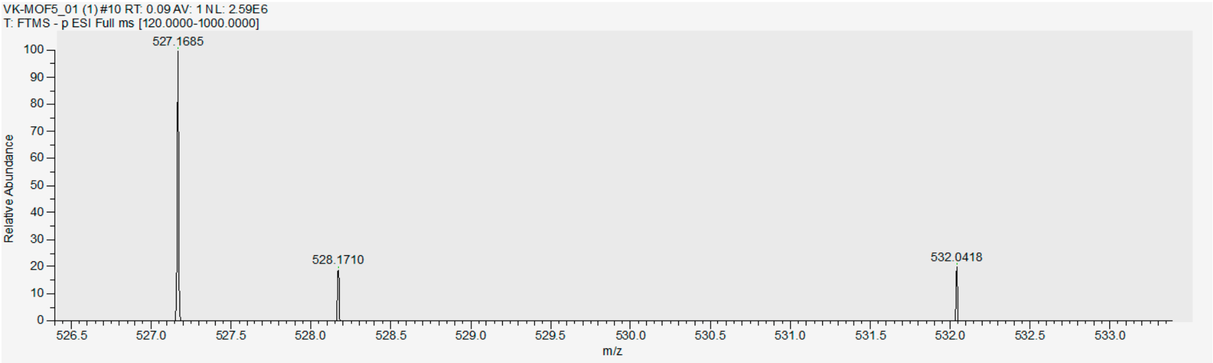

Figure S83. HRMS (HESI$^-$) spectrum of ligand **3h**.

HRMS spectra of ligand **3i**

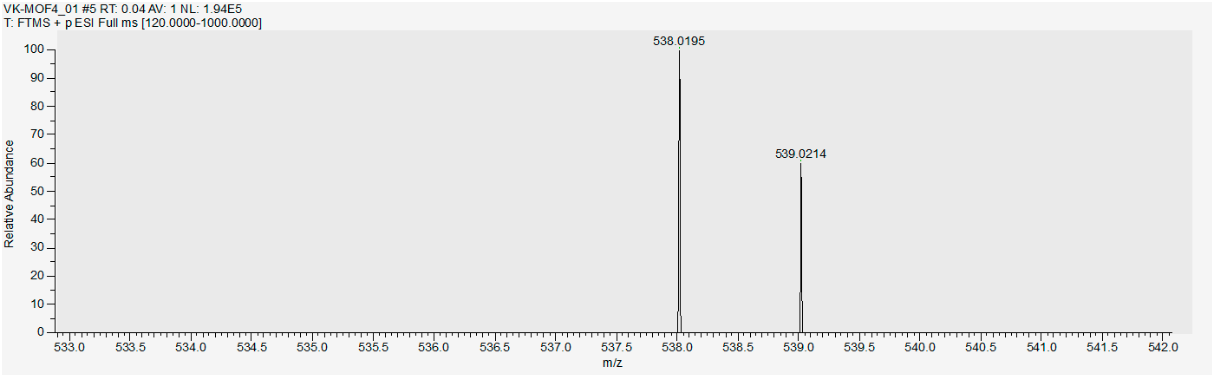

Figure S84. HRMS (HESI$^+$) spectrum of ligand **3i**.

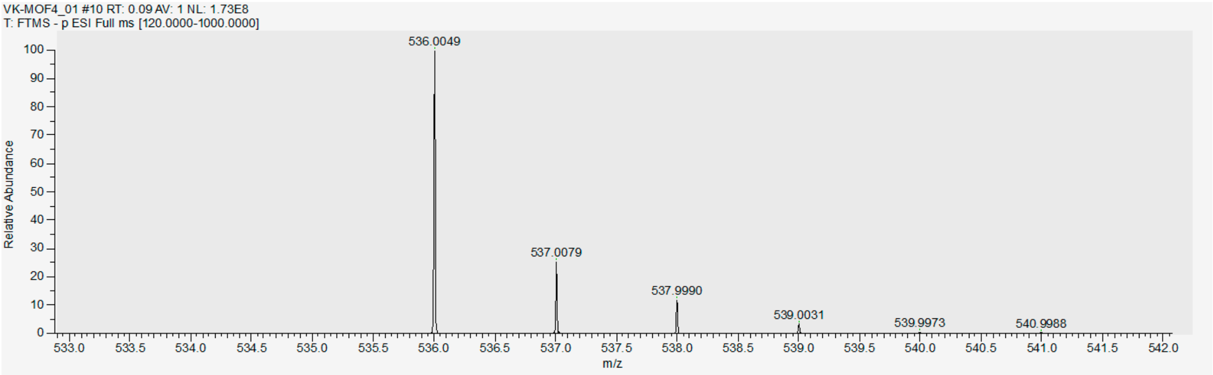

Figure S85. HRMS (HESI$^-$) spectrum of ligand **3i**.

HRMS spectra of ligand **3j**

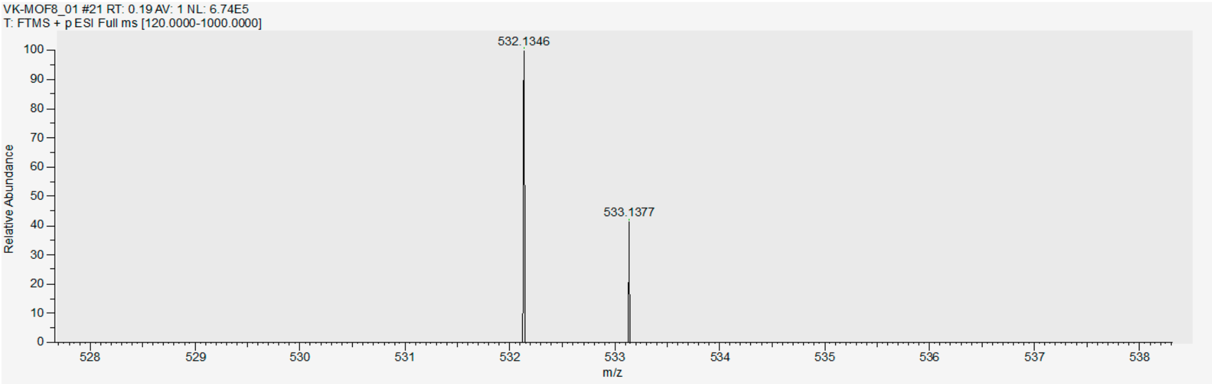

Figure S86. HRMS (HESI^{+}) spectrum of ligand **3j**.

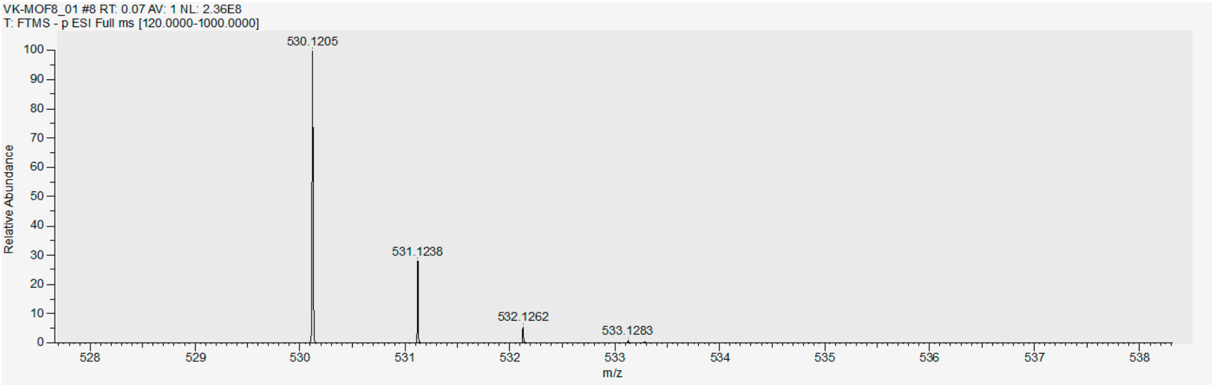

Figure S87. HRMS (HESI^{-}) spectrum of ligand **3j**.

HRMS spectra of ligand **3k**

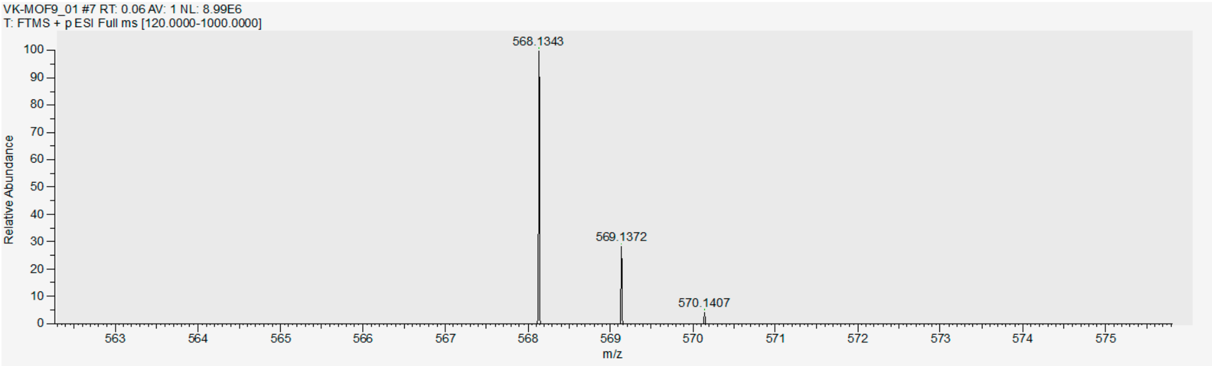

Figure S88. HRMS (HESI^{+}) spectrum of ligand **3k**.

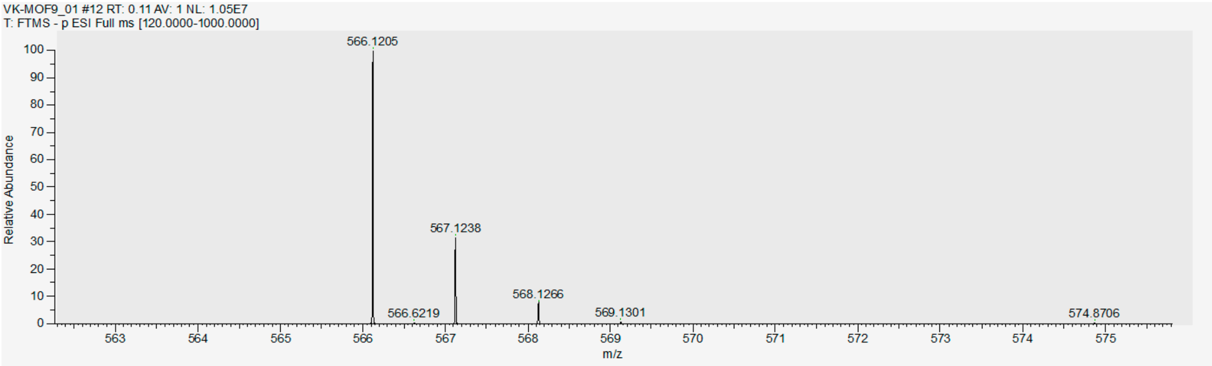

Figure S89. HRMS (HESI^{-}) spectrum of ligand **3k**.

HRMS spectra of ligand **3l**

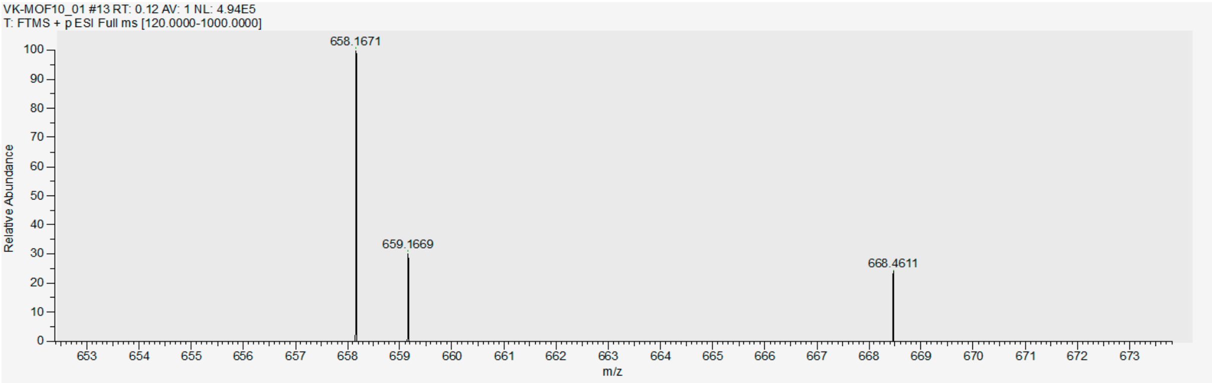

Figure S90. HRMS (HESI^{+}) spectrum of ligand **3l**.

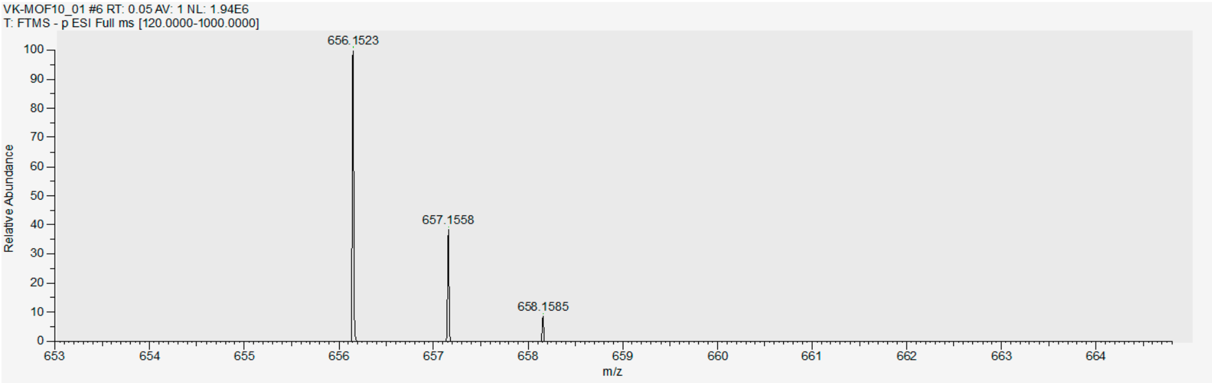

Figure S91. HRMS (HESI^{-}) spectrum of ligand **3l**.

HRMS spectra of ligand **3m**

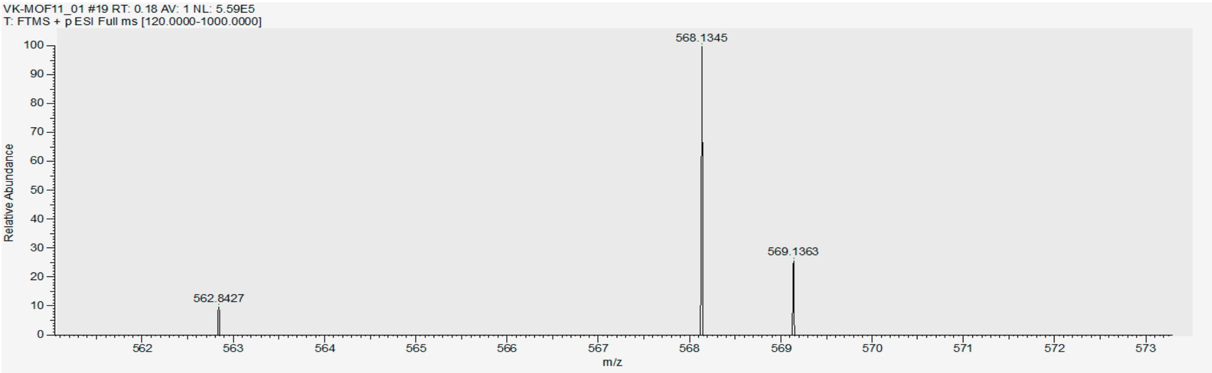

Figure S92. HRMS (HESI^{+}) spectrum of ligand **3m**.

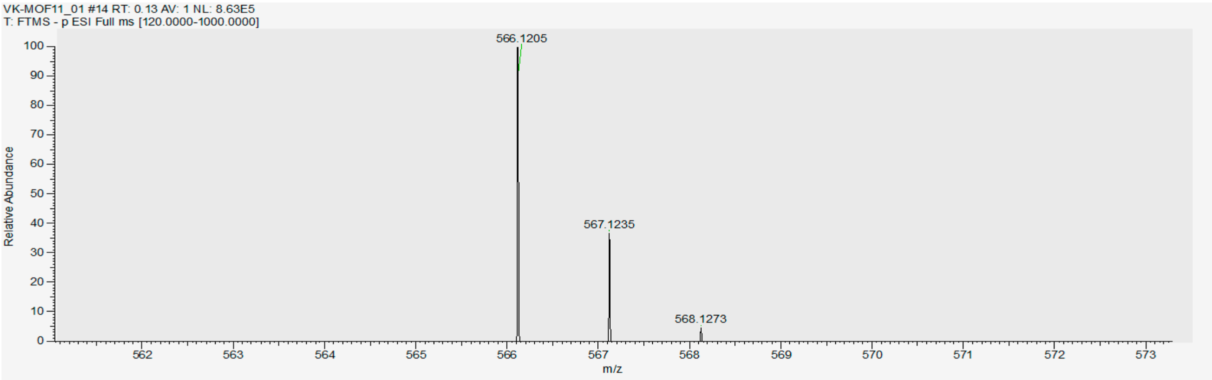

Figure S93. HRMS (HESI^{-}) spectrum of ligand **3m**.

HRMS spectra of ligand **3n**

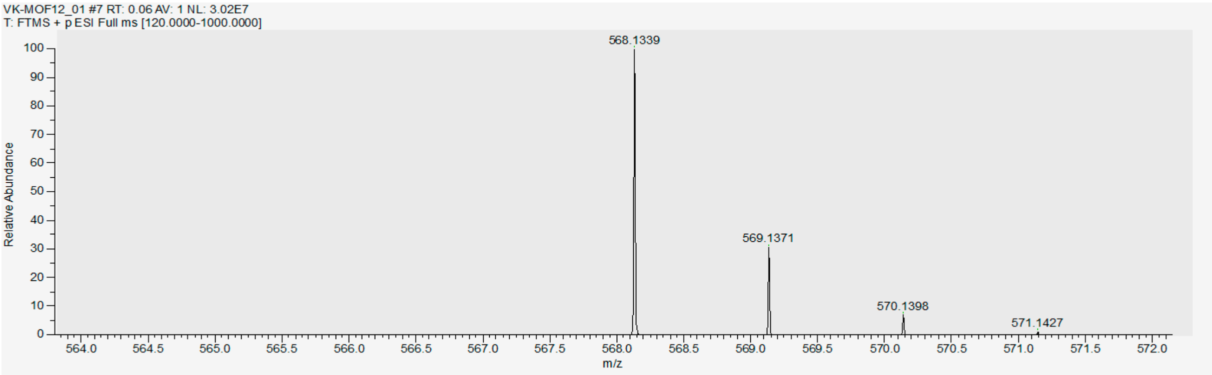

Figure S94. HRMS (HESI^{+}) spectrum of ligand **3n**.

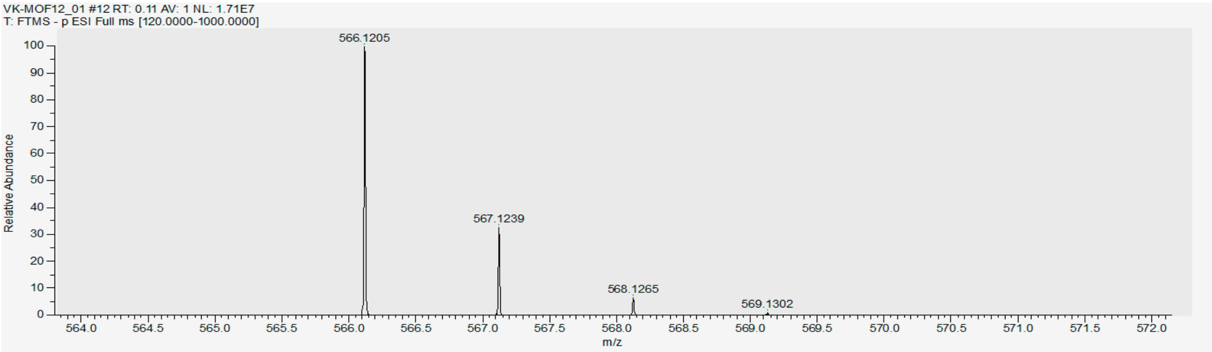

Figure S95. HRMS (HESI^{-}) spectrum of ligand **3n**.

HRMS spectra of ligand **3o**

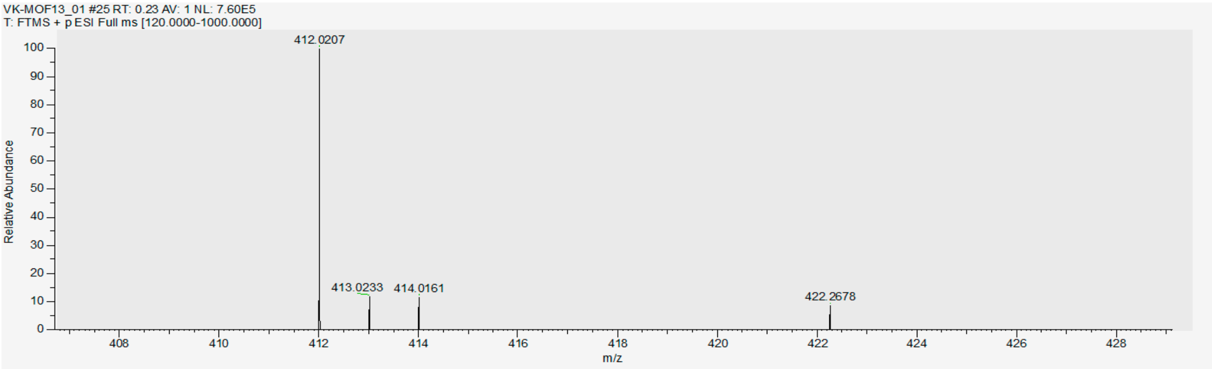

Figure S96. HRMS (HESI^{+}) spectrum of ligand **3o**.

HRMS spectra of ligand **3p**

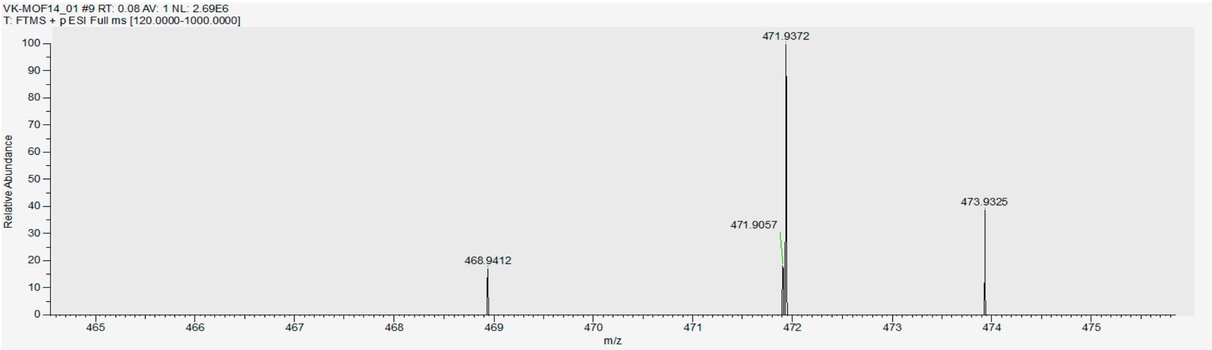

Figure S97. HRMS (HESI^{+}) spectrum of ligand **3p**.

HRMS spectra of ligand **3q**

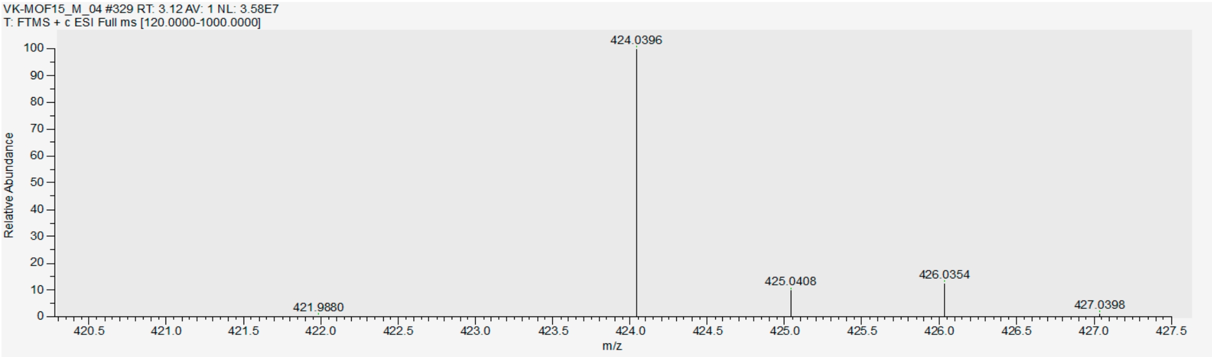

Figure S98. HRMS (HESI^{+}) spectrum of ligand **3q**.

HRMS spectra of ligand **3r**

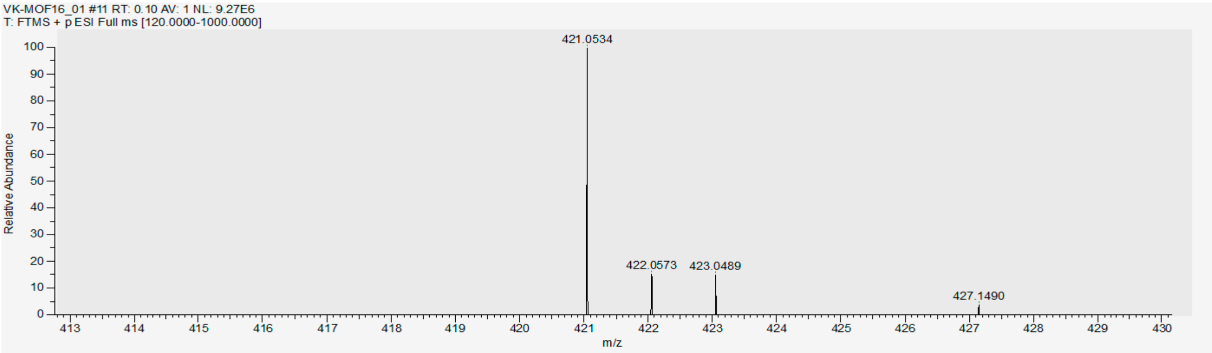

Figure S99. HRMS (HESI^{+}) spectrum of ligand **3r**.

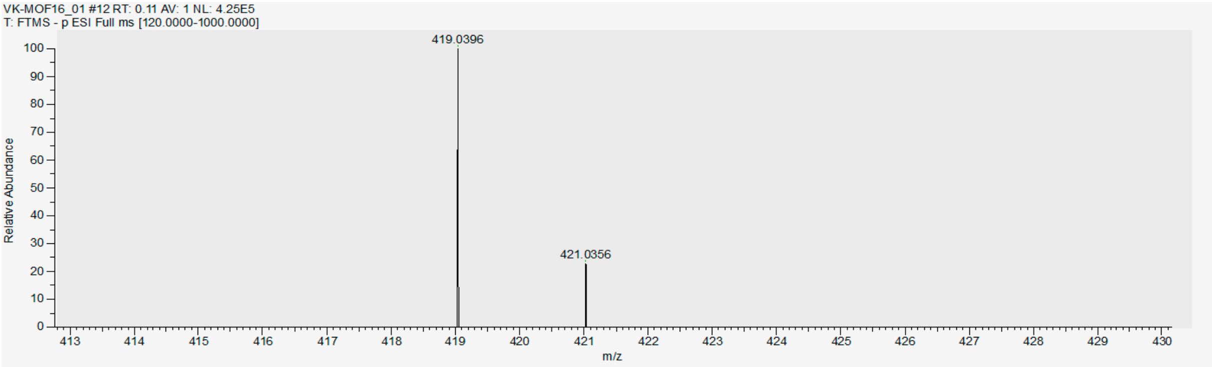

Figure S100. HRMS (HESI^{-}) spectrum of ligand **3r**.

HRMS spectra of ligand **3s**

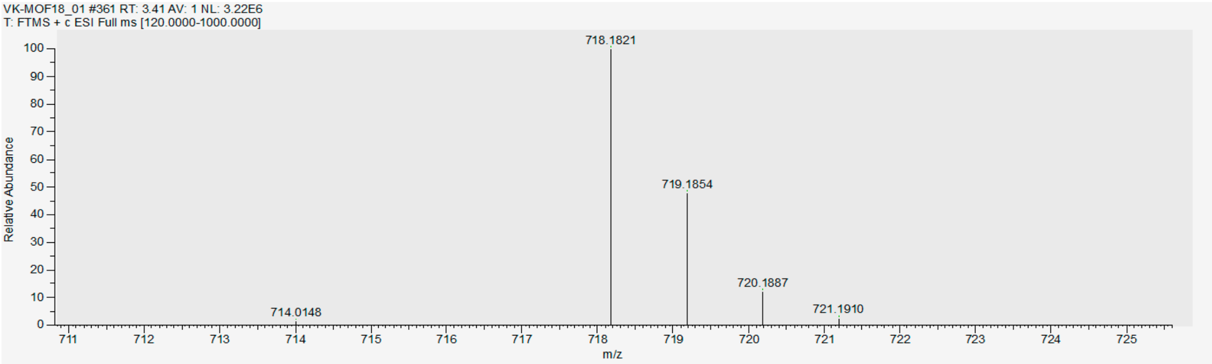

Figure S101. HRMS (HESI^{+}) spectrum of ligand **3s**.

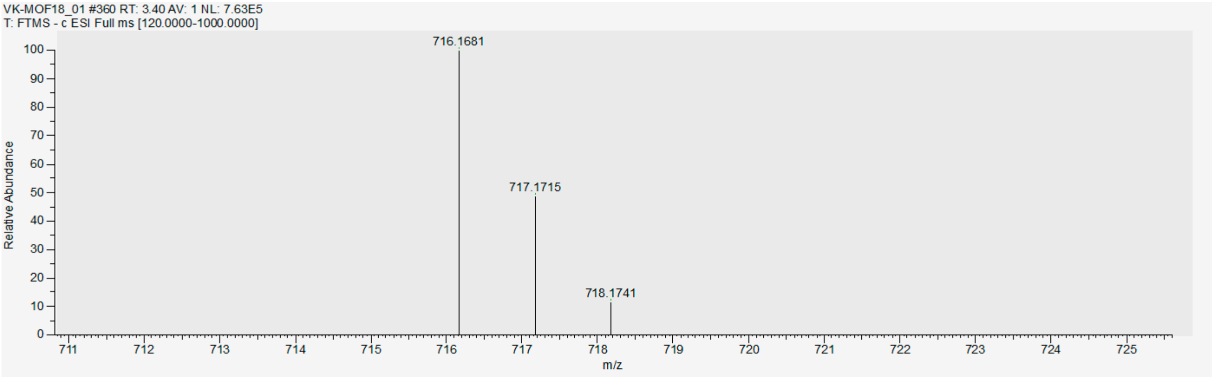

Figure S102. HRMS (HESI$^-$) spectrum of ligand **3s**.

<u>HRMS spectra of ligand **3t**</u>

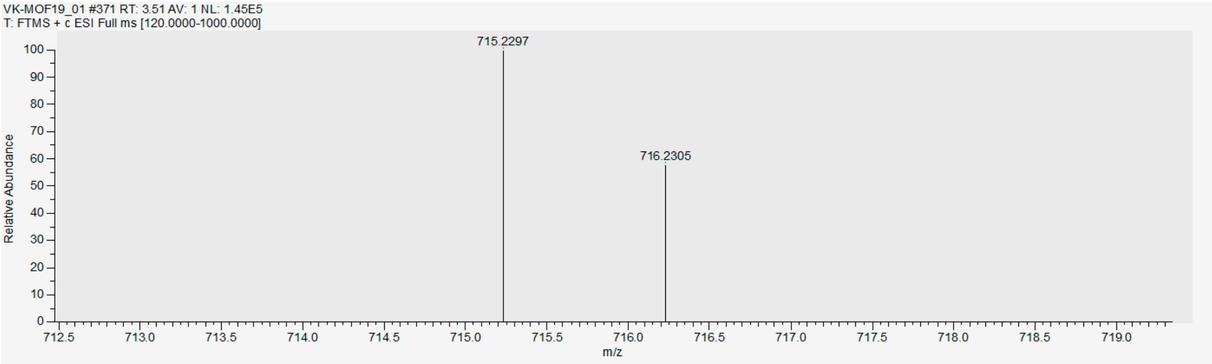

Figure S103. HRMS (HESI$^+$) spectrum of ligand **3t**.

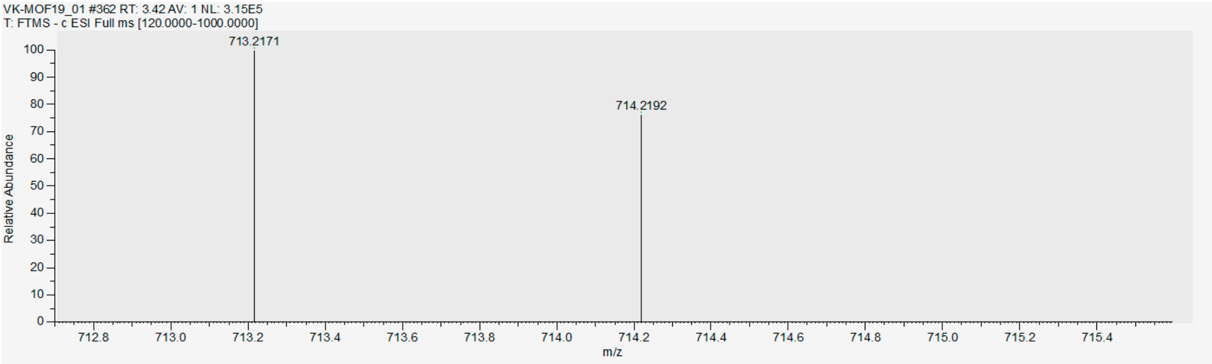

Figure S104. HRMS (HESI$^-$) spectrum of ligand **3t**.

<u>HRMS spectra of ester **4**</u>

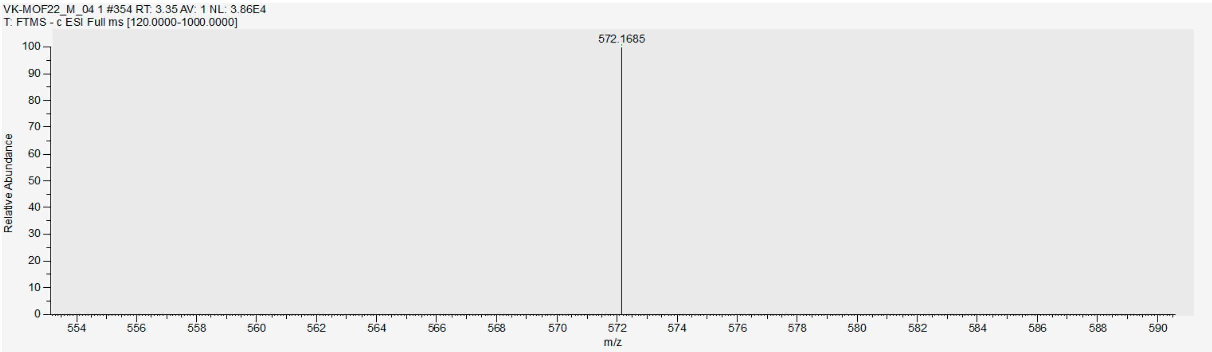

Figure S105. HRMS (HESI$^-$) spectrum of ester **4**.
